# MicroRNAs and their putative targets in *Brassica napus* seed maturation

**DOI:** 10.1186/1471-2164-14-140

**Published:** 2013-02-28

**Authors:** Daiqing Huang, Chushin Koh, J Allan Feurtado, Edward WT Tsang, Adrian J Cutler

**Affiliations:** 1 Plant Biotechnology Institute, National Research Council of Canada, 110 Gymnasium Place, Saskatoon S7N 0W9, Canada

**Keywords:** Seed development, Embryo, Next generation sequencing

## Abstract

**Background:**

MicroRNAs (miRNAs) are 20–21 nucleotide RNA molecules that suppress the transcription of target genes and may also inhibit translation. Despite the thousands of miRNAs identified and validated in numerous plant species, only small numbers have been identified from the oilseed crop plant *Brassica napus* (canola) – especially in seeds.

**Results:**

Using next-generation sequencing technologies, we performed a comprehensive analysis of miRNAs during seed maturation at 9 time points from 10 days after flowering (DAF) to 50 DAF using whole seeds and included separate analyses of radicle, hypocotyl, cotyledon, embryo, endosperm and seed coat tissues at 4 selected time points. We identified more than 500 conserved miRNA or variant unique sequences with >300 sequence reads and also found 10 novel miRNAs. Only 27 of the conserved miRNA sequences had been previously identified in *B. napus* (miRBase Release 18). More than 180 MIRNA loci were identified/annotated using the *B. rapa* genome as a surrogate for the *B.napus* A genome. Numerous miRNAs were expressed in a stage- or tissue-specific manner suggesting that they have specific functions related to the fine tuning of transcript abundance during seed development. miRNA targets in *B. napus* were predicted and their expression patterns profiled using microarray analyses. Global correlation analysis of the expression patterns of miRNAs and their targets revealed complex miRNA-target gene regulatory networks during seed development. The miR156 family was the most abundant and the majority of the family members were primarily expressed in the embryo.

**Conclusions:**

Large numbers of miRNAs with diverse expression patterns, multiple-targeting and co-targeting of many miRNAs, and complex relationships between expression of miRNAs and targets were identified in this study. Several key miRNA-target expression patterns were identified and new roles of miRNAs in regulating seed development are suggested. miR156, miR159, miR172, miR167, miR158 and miR166 are the major contributors to the network controlling seed development and maturation through their pivotal roles in plant development. miR156 may regulate the developmental transition to germination.

## Background

Non-coding small RNAs (sRNAs) are important regulators of gene expression in eukaryotes and influence almost all aspects of plant biology [1-3]. There are two major classes of endogenous sRNAs: microRNAs (miRNAs) and short-interfering RNAs (siRNAs), typically of 21 to 24 nucleotides in length.

miRNAs are the best understood class of sRNAs. They differ from other types of sRNAs in their biogenesis. In plants, miRNAs are generated from primary miRNA transcripts (pri-miRNAs) that are generally transcribed by RNA polymerase II and fold back on themselves to form distinctive hairpin structures [1]. Primary transcripts are first processed (trimmed) by DICER-LIKE1 (DCL1) to miRNA precursors (pre-miRNAs) in the nucleus, which are exported to the cytoplasm and then further excised by DCL1 to generate ~21nt mature miRNAs [4]. Plant miRNAs suppress gene expression mainly by directing cleavage of their highly complementary target transcripts. Recent studies suggest that translational repression may be also common in plants [5,6].

The number of newly discovered miRNAs is growing rapidly [7-9]. At the time of writing, 18,226 mature miRNAs have been discovered and deposited in the public miRNA database miRBase (Release 18.0, http://microrna.sanger.ac.uk/sequences/index.shtml). These miRNAs include 4,014 miRNAs from 50 flowering plant species. The majority were obtained from model species with sequenced genomes such as *Medicago truncatula* (635), *Oryza sativa* (581), *Glycine max* (362), *Populus trichocarpa* (234), *Arabidopsis thaliana* (291), *Physcomitrella patens* (229) and *Arabidopsis lyrata* (201).

*Brassica napus* (canola) is the third largest oilseed crop in the world, providing approximately 13% of the world's supply of vegetable oil [10]. Several recent studies have contributed to the identification of miRNAs from *B. napus*. Xie et al. [11] used computational methods to predict 21 potential *B. napus* miRNAs. Wang et al. [12] cloned and identified 11 conserved miRNA families and examined their expression patterns in five double haploid *B. napus* lines. Buhtz et al. [13] identified 32 miRNAs from 18 different families and a set of unknown sRNAs from *B. napus* phloem sap. At the time of writing*,* 44 miRNAs (27 unique sequences) corresponding to 17 families from *B. napus* have been deposited in the miRBase database (Release18). During revision of this paper, more miRNAs were identified from the early siliques of two *B. napus* cultivars differing in oil content [14], from *B. napus* seedlings exposed to heavy metals [15] and from pooled *B. napus* tissues [16]. To date, 53 unique miRNA sequences representing 90 miRNAs from 34 families have been deposited in the miRBase (Release 19). Considering the estimated size of the *B. napus* genome (1,132Mbp) [17] and the small numbers of identified miRNAs so far, it can be assumed that many more *B. napus* miRNAs remain to be discovered, especially in seed. To understand the regulatory roles of miRNAs in *B. napus*, it is important to expand the collection of miRNAs and profile their temporal and spatial expression.

Seed maturation covers the period from the end of embryo development to the mature dry seed and occurs from about 15 to 45 days after flowering (DAF). It can be partitioned into two over-lapping phases, seed filling followed by seed desiccation. Processes occurring in maturation affect seed size, oil production, protein content and antinutritional accumulation, as well as seedling vigor following imbibition of the dry seed for subsequent plant growth. Recent gene expression studies using microarrays have shown that there are vast gene expression changes during seed filling and desiccation in *B. napus*. Distinct expression patterns related to carbohydrate metabolism, lipid biosynthesis, and storage protein accumulation have been documented [18-20]. However, expression and regulatory functions of miRNAs in *Brassica* seed development, especially during seed maturation, are unknown.

The high throughput and sensitivity of next generation sequencing (NGS) technologies make them powerful tools for both discovery of novel miRNAs and genome-wide profiling of conserved miRNAs. To discover and characterize the miRNAs during *Brassica* seed development, NGS technologies were used for miRNA discovery and profiling during seed maturation in *B. napus*. Comprehensive analyses of miRNAs and their targets identified many new miRNAs and delivered new insights into their role in seed development and maturation.

## Results

### Sequencing statistics

For maximum sequencing depth, we used two technologies: Sequencing by Oligonucleotide Ligation and Detection (SOLiD, developed by Applied Biosystems) and Sequencing by Synthesis (SBS, developed by Illumina). Small RNA libraries were prepared from 9 seed developmental stages (10, 15, 20, 25, 30, 35, 40, 45 and 50 DAF) plus floral buds and these libraries were sequenced using SOLiD. To investigate the temporal and spatial expression of miRNAs, we further dissected seeds into embryo (Em), endosperm (Endo) and seed coat (SC) tissues at 4 selected seed stages (15, 25, 35 and 45 DAF); in addition, the embryo was separated into radicle (R), hypocotyl (H) and cotyledon (C) at 25 and 45 DAF. These dissected samples were used to prepare 17 additional small RNA libraries that were sequenced using SBS. The sequencing results from both SOLiD and SBS are summarized in Table 1. In total, we obtained 88 M reads from 10 SOLiD libraries (9 stages of whole seeds and flower buds) and 295 M reads from 17 SBS libraries (dissected seed compartments). After removing low quality reads and adaptors from the raw sequences, we obtained 234 M SBS and 74 M SOLiD clean sequence reads, among which ~175 M SBS and ~17 M SOLiD sequences were of 19–25 nt in length.

**Table 1:** Sequencing statistics

**Sequencing platform**	**Library**	**Total reads**	Clipped*	Cleaned**
**SOLiD dataset**	flower buds	12,801,066	11,282,118	11,282,091
	10d	13,654,854	11,230,801	11,230,779
	15d	8,183,585	6,823,454	6,823,440
	20d	7,454,670	6,194,847	6,194,827
	25d	9,243,505	8,271,307	8,271,295
	30d	7,130,691	6,074,794	6,074,781
	35d	6,436,710	5,370,745	5,370,732
	40d	6,944,646	5,789,097	5,789,076
	45d	7,705,418	6,954,348	6,954,334
	50d	8,749,906	6,829,547	6,829,528
	Subtotal	88,305,051	74,821,058	74,820,883
**Illumina dataset**	15DEm	14,034,094	10,378,052	10,371,178
	15DEndo	24,739,789	19,000,135	18,916,066
	15DSC	17,442,131	14,601,621	14,594,202
	25 DC	15,168,491	10,832,772	10,831,009
	25DEm	11,925,734	10,937,990	10,936,745
	25DEndo	15,610,323	13,157,907	13,155,132
	25DH	15,885,961	13,183,381	13,180,455
	25DR	15,496,019	13,958,721	13,955,227
	25DSC	12,782,271	11,308,036	11,306,170
	35DEm	16,913,533	15,482,754	15,476,635
	35DEndo	17,644,795	10,178,579	10,173,946
	35DSC	16,381,235	11,488,796	11,483,795
	45 DC	15,997,842	13,743,381	13,633,211
	45DEm	17,405,343	13,556,990	13,543,113
	45DH	25,222,060	20,437,149	20,369,083
	45DR	24,660,758	22,099,699	22,092,547
	45DSC	17,770,899	10,608,884	10,603,168
	Subtotal	295,081,278	234,954,847	234,621,682
**Total**		383,386,329	309,775,905	309,442,565

Notes:

1) *Discarded (clipped) Illumina Adaptor: ATCTCGTATGCCGTCTTCTGCTTG (length < 19nt, adaptor & Ns only reads).

2) *Clipped Solid Adaptor: CGCCTTGGCCGTACAGCAG (length < 19nt, adaptor & Ns only reads).

3) **Cleaned single nucleotide repeats (poly As, Cs, Ts, Gs).

### Size distribution of small RNAs

**Figure 1:**
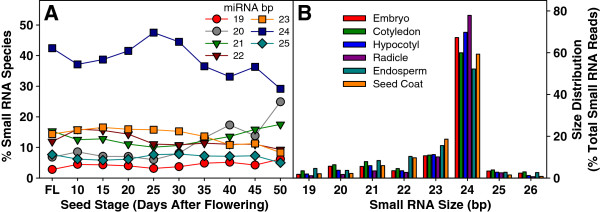
**Size distribution of small RNA (sRNA). A**. Changes in sRNA size abundance (bp) in flower buds and seeds at 9 developmental stages. **B**. Changes in abundance of sRNA species in various seed tissues at 25DAF.

As expected, *B. napus* has a complex small RNA population (Figure 1A). The most abundant length of small RNAs in the whole seed was 24nt followed by 23nt and 21nt at all development stages (Figure 1B). This is consistent with previous reports from other species [21]. The 24nt population increased in early seed development until 25DAF and then declined from 25DAF to mature seeds. The 24nt small RNAs were the most abundant in all tissue types and were extremely high in the radicle of the embryo, reaching 76% of the total small RNA (19nt – 25nt) reads. The proportion of 24 nt small RNAs was lower in endosperm - about 50%. The 24nt RNAs mainly consist of siRNAs that are associated with the silencing of repeat sequences and transposons [21]. Higher levels of 24nt RNAs in the developing and mature embryo (especially in the radicle) compared to other tissue types suggest that repression of repeats and transposons in the embryo is important during seed development.

### Identification of conserved miRNAs

Many miRNAs are evolutionarily conserved in the plant kingdom [22,23]. To identify conserved miRNAs in *B. napus*, all processed (clean) sequences from the 27 small RNA libraries were pooled and searched for the presence of known miRNAs listed in miRBase (Release 18). 279 unique sequences showed perfect matches to known miRNAs from 49 miRNA families (with a minimum of 2 reads) (Additional file 1: Table S1). 222 of them are a perfect match to miRNA mature sequences and 57 of them to miRNA star (*) sequences (* sequences are complementary, opposite strand sequences to miRNAs that do not direct cleavage). At the time of writing, 46 miRNA entries (27 unique mature sequences) from 17 miRNA families (minimum of 2 reads) in *B. napus* have been deposited in miRBase (Release 18). In this study, we detected 26 out of these 27 mature miRNA sequences, covering 44 of 46 entries and all of the 17 miRNA families. In addition, we found 253 miRNA orthologs that have never been reported in *B. napus*. This has substantially expanded the collection of miRNAs in *B. napus*. From the 279 conserved miRNA sequences, 73, 109, 51, 51, 44, and 56 were exact matches to mature miRNA sequences from *A. thaliana* (ath), *A. lyrata* (aly), *O. sativa* (osa), *P. trichocarpa* (ptc), *M. trunculata* (mtr) and *G. max* (gma) respectively. Among the 109 sequences that matched to *A. lyrata*, 42 were miRNA* sequences. The high proportion of miRNA* sequence matches was due to the fact that most miRNA* sequences in miRBase are from *A. lyrata*. Usually the miRNA sequences are more abundant than their star sequences. The large coverage of miRNAs in seeds indicates that many miRNAs are involved in seed development.

**Figure 2:**
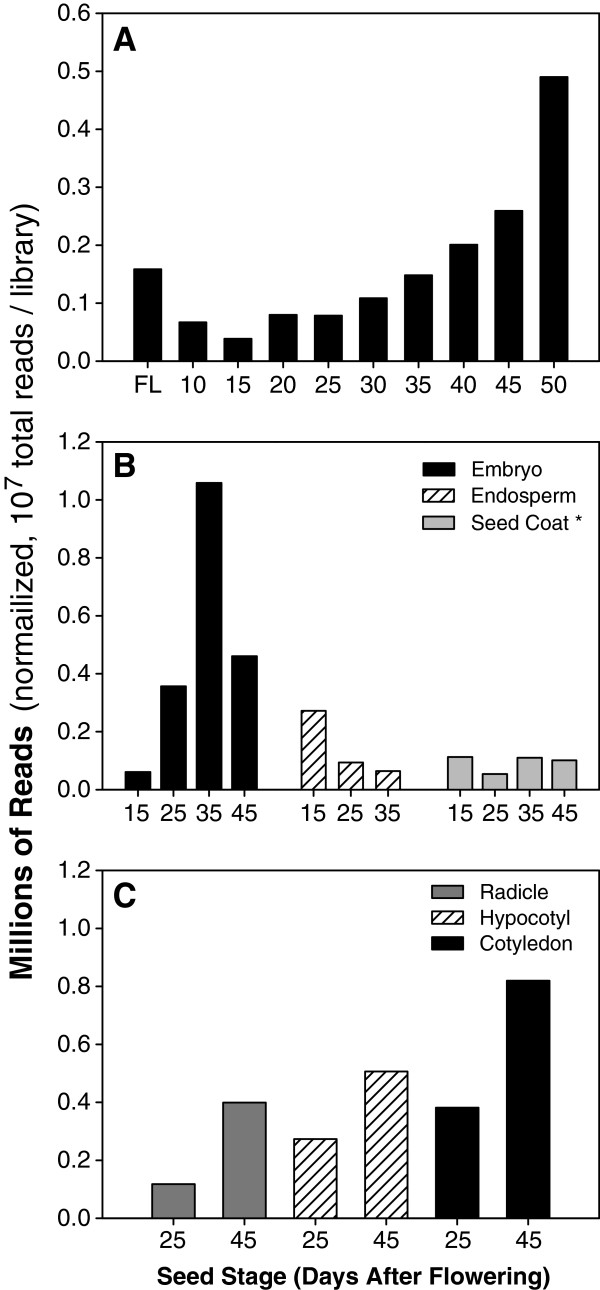
**Total sequencing reads in each library (normalized to 10 M). A**. Total reads in flower buds and at 9 seed developmental stages. **B**. Total reads in embryo, endosperm and seed coat at 4 seed developmental stages. **C**. Total reads in radicle, hypocotyl and cotyledon of embryo at 25DAF and 45DAF.

The total number of reads from conserved miRNAs in whole seeds (SOLiD dataset) decreased from flower buds to 15DAF seeds, but then increased substantially during seed development, especially in seed maturation (Figure 2A). The total numbers of conserved reads from dissected seed parts at 4 stages are shown in Figure 2B. miRNAs were more abundant in endosperm than in embryo and seed coat at 15DAF. However, at later stages, miRNAs were substantially more abundant in embryo than in endosperm or seed coat. The total number of miRNA reads in embryo increased significantly until 35DAF and then decreased at 45DAF. Within the embryo, miRNAs are most abundant in the cotyledons, followed by hypocotyl and radicle (Figure 2C).

### Many additional mature miRNAs

In addition to exact matches to mature or mature star sequences annotated in miRBase (Release 18), there were numerous other abundant mature miRNA sequences. Most of these sequences differed from the annotated miRNAs by only a single nucleotide and could usually be mapped to the corresponding annotated precursors (identification of precursor sequences is described later). Allowing one mismatch with the annotated MIRNAs in miRBase (Release 18), we identified 11,478 miRNA-like sequences with at least 2 reads. Among them, 6,433 were exact matches and 5,045 differed from the annotated miRNA or miRNA* by one nucleotide (Additional file 2: Table S2). The read counts of these sequences varied from 2 to 379,019. While some of these sequences might be miRNA sequence variants produced from imprecise cleavage during miRNA biogenesis, the high abundance of many of these sequences suggests that they may be either independent mature miRNAs or, more likely, that they are the ‘true’ mature miRNAs in *B. napus*, rather than the annotated miRNAs from other species. For example, two sequence variants of miR158 (with 379,019 and 35,353 reads), are both more abundant than the sequence that is annotated as ath-miR158a (18,654 reads), indicating they both are very likely miR158 mature sequences. Furthermore, a variant of miR827 has 59,977 reads while the exact ath-miR827 sequence has only 1,001 reads, indicating the former sequence is more likely the functional miRNA in *B. napus* during seed maturation. Therefore we defined these sequence variants as conserved miRNAs and focused on the miRNA/variant sequences with >300 reads for further profiling analyses (Additional file 3: Table S3).

### Abundance of conserved miRNA families in seeds

**Figure 3:**
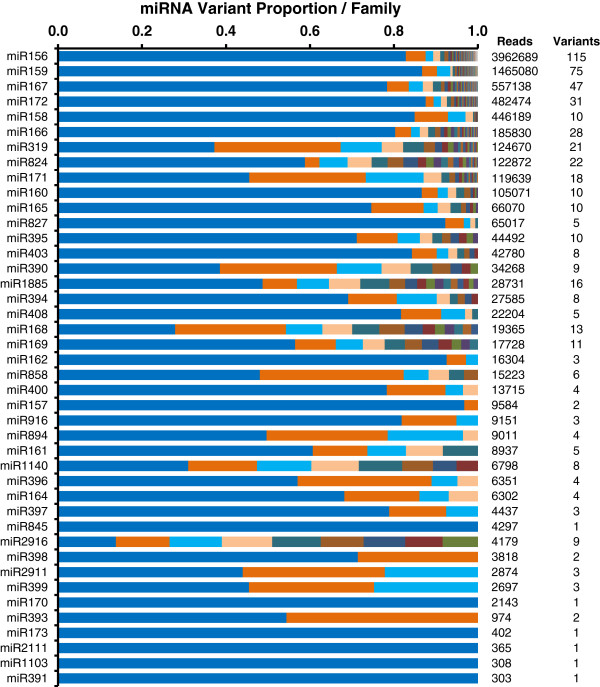
**Proportion of miRNA variants in each miRNA family.** 42 miRNA families with total reads more than 300 are shown in order of decreasing abundance (only variants with reads >300 are included in the analysis).

The abundance of miRNAs varied enormously between miRNA families, ranging from less than 10 to several million reads (Additional file 1: Table S1 and Additional file 2: Table S2). The most frequently sequenced miRNAs (total read counts >3000) are listed in Table 2. The total number of sequence reads from all miRNA variants (>300 reads) within each miRNA family were pooled for abundance analysis. Within each family, the proportion of the total reads accounted for by the major variants (>300 reads) are shown in Figure 3 as well as the total number of reads for each family and the number of variants. The miRNA156 family was the most abundant in seed followed by the miR159, miR172, miR167 and miR158 families. The 5 top miRNA families accounted for 49%, 18%, 7%, 6% and 5% of the total reads from all conserved seed miRNAs, respectively. The remaining miRNA families together account for approximately 15% of total conserved miRNA reads. The number of miRNA variants in each miRNA family was highly variable (Figure 3). For example, there were 115, 75, 47, 31 and 28 unique variant sequences (with reads >300) in the miR156, miR159, miR167, miR172 and miR166 families respectively, but only one miRNA sequence in the miR845, miR170, miR173 and miR391 families. Usually, one or two miRNA variant sequences predominated within each family (Figure 3). For example, ath-miR156a, ath-miR159a, ath-miR172c, ath-miR167a and bna-miR158b were the predominant variants in the miR156, miR159, miR172, miR167 and miR158 families. This suggests that, in most cases, the regulatory role of each family is mostly performed by the dominant miRNA variants. A conspicuous exception to this relationship appears to be miR2916 but later mapping analysis revealed this not to be a typical miRNA.

**Table 2:** **The most frequently sequenced miRNA/variants in *B. napus *seeds (>3000 total reads)**

**miRNA family**	**miRNA sequence**	**Given miRNA name**	**Identical sequence (Mature)**	**Homologous MIRNA (Precursor)**	**Total reads**
**miR156**	TGACAGAAGAGAGTGAGCAC	bna-miR156_v1	ath-miR156a	ath-MIR156a	3282238
	TGACAGAAGAGAGTAAGCAC	bna-miR156_v2		ath-MIR156a	187170
	TGACAGAAGAGAGTGAACAC	bna-miR156_v3		ath-MIR156c	71366
	TTGACAGAAGAAAGAGAGCAC	bna-miR156_v4	smo-miR156c	smo-MIR156c	66413
	TGACAGAAGAGAGTGAGCACA	bna-miR156_v5	bna-miR156a	bna-MIR156c	39023
	TGACAGAAGAGAGTCAGCAC	bna-miR156_v6		osa-MIR156c	38941
	TGACAGAAGAGAGGGAGCAC	bna-miR156_v7	ptc-miR156k	ptc-MIR156k	18907
	TGACAGAAGAGAGTGAGCAA	bna-miR156_v8		osa-MIR156e	15166
	TGACAGAAGAGAGTGAGCAT	bna-miR156_v9		ath-MIR156c	14021
	TGACAGAAGAGAGTTAGCAC	bna-miR156_v10		ssp-MIR156	13172
	TGACAGAAGAGAGTGAGAAC	bna-miR156_v11		ath-MIR156a	10071
	TGACAGAAGAGAGTGAGCACC	bna-miR156_v12		bdi-MIR156c	9628
	TTGACAGAAGAGAGTGAGCAC	bna-miR156_v13	gma-miR156k	gma-MIR156k	9212
	TGCCAGAAGAGAGTGAGCAC	bna-miR156_v14		gma-MIR156h	8191
	TGACAGAAGAGAGCGAGCAC	bna-miR156_v15	zma-miR156k	zma-MIR156k	7663
	CGACAGAAGAGAGTGAGCAC	bna-miR156_v16	ath-miR156g	ath-MIR156g	6529
	TGACAGAAGAAAGAGAGCAC	bna-miR156_v17	ath-miR156h	ath-MIR156h	5933
	TGACAGAAGAGAGGGAGCAA	bna-miR156_v18		vvi-MIR156a	5778
	TTGACAGAAGAGAGCGAGCAC	bna-miR156_v19		sbi-MIR156e	5582
	GCTCACTGCTTTATCTGTCAGA	bna-miR156_v20		aly-MIR156c	5492
	TGACAGAAGAGAGTGGGCAC	bna-miR156_v21		zma-MIR156i	5326
	GCTCACTGCTCTTTCTGTCAGA	bna-miR156_v22	aly-miR156a*	aly-MIR156a	5242
	TGACAGAAGAGAGTGAGCGC	bna-miR156_v23		osa-MIR156b	5198
	TGACAGAAGAGAGTGTGCAC	bna-miR156_v24		osa-MIR156i	4981
	TGACAGAAGAGAGTGAGCCC	bna-miR156_v25		ctr-MIR156	4782
	TTGACAGAAGAGACCGAGCAC	bna-miR156_v26		zma-MIR156k	3734
	TTGACAGAAGAAAGAAAGCAC	bna-miR156_v27		ath-MIR156h	3729
	TGACAGAAGAGAGTGAGCACT	bna-miR156_v28		vvi-MIR156e	3661
	TGACAGAAGAGAGTGCGCAC	bna-miR156_v29		ath-MIR156f	3555
	TGACATAAGAGAGTGAGCAC	bna-miR156_v30		ath-MIR156a	3082
	TGACAGAAGAGAGTGACCAC	bna-miR156_v31		zma-MIR156i	3043
**miR157**	TTGACAGAAGATAGAGAGCAC	bna-miR157_v1	ath-miR157a	ath-MIR157c	9274
**miR158**	TTTCCAAATGTAGACAAAGCA	bna-miR158_v1		ath-MIR158a	379019
	TTTCCAAATGTAGACAAAGC	bna-miR158_v2		aly-MIR158a	35353
	TCCCAAATGTAGACAAAGCA	bna-miR158_v3	ath-miR158a	ath-MIR158a	18654
	TTCCAAATGTAGACAAAGCA	bna-miR158_v4		ath-MIR158a	8078
**miR159**	TTTGGATTGAAGGGAGCTCTA	bna-miR159_v1	ath-miR159a	ath-MIR159a	1270354
	TTTGGATTGAAGGGAGCTCT	bna-miR159_v2		bra-MIR159a	51891
	TTTGGATTGAAGGGAACTCTA	bna-miR159_v3		csi-MIR159	46735
	TTTGGATTGAAGGGAGCCCTA	bna-miR159_v4		bna-MIR159	8153
	TTTGGATTGAAGGGACCTCTA	bna-miR159_v5		ptc-MIR159a	7927
	TTTGTATTGAAGGGAGCTCTA	bna-miR159_v6		bna-MIR159	6853
	TTTGGATTGAAGGGAGATCTA	bna-miR159_v7		ptc-MIR159a	3935
	CTTGCATATCTTAGGAGCTTT	bna-miR159_v8		ptc-MIR159c	3806
	TTTGGATTGAAGGGAGCACTA	bna-miR159_v9		bna-MIR159	3448
**miR160**	TGCCTGGCTCCCTGTATGCCA	bna-miR160_v1	ath-miR160a	ath-MIR160a	91013
	TGCCTGGCTCCCTGTATACCA	bna-miR160_v2		zma-MIR160e	3993
**miR161**	TCAATGCACTGAAAGTGACTA	bna-miR161_v1	bna-miR161	bna-MIR161	5423
**miR162**	TCGATAAACCTCTGCATCCAG	bna-miR162_v1	ath-miR162a	ath-MIR162a	15091
**miR164**	TGGAGAAGCAGGGCACGTGCA	bna-miR164_v1	ath-miR164a	ath-MIR164a	4298
**miR165**	TCGGACCAGGCTTCATCCCCC	bna-miR165_v1	ath-miR165a	ath-MIR165a	49308
	TCGGACCAGGCTTCATCCCC	bna-miR165_v2	aly-miR165a	aly-MIR165a	8234
**miR166**	TCGGACCAGGCTTCATTCCCC	bna-miR166_v1	ath-miR166a	ath-MIR166a	149247
	GGAATGTTGTCTGGCTCGAGG	bna-miR166_v2	zma-miR166c*	zma-MIR166c	7006
	TCGGACCAGGCTTCATACCCC	bna-miR166_v3		zma-MIR166d	3864
	GTCGGACCAGGCTTCATTCCC	bna-miR166_v4		aly-MIR166a	3710
	TCGGACCAGGCTTCATTCCC	bna-miR166_v5	zma-miR166h	zma-MIR166h	3039
	TGAAGCTGCCAGCATGATCTA	bna-miR167_v1	ath-miR167a	ath-MIR167a	436575
**miR167**	TGAAGCTGCCAGCATAATCTA	bna-miR167_v2		gma-MIR167d	28902
	TGAAGCTGCCAGCATGACCTA	bna-miR167_v3		bdi-MIR167a	18620
	GATCATGTTCGCAGTTTCACC	bna-miR167_v4	aly-miR167a*	aly-MIR167a	13484
	TGAAGCTGCCAGCATGATCT	bna-miR167_v5		gma-MIR167g	10622
	TGAAGCTGCCAGCATCATCTA	bna-miR167_v6		bna-MIR167b	4726
	TGAAGCTGCCAGCATGAACTA	bna-miR167_v7		mtr-MIR167	4258
	GAAGCTGCCAGCATGATCTA	bna-miR167_v8		zma-MIR167c	4011
**miR168**	TCGCTTGGTGCAGGTCGGGAC	bna-miR168_v1		bdi-MIR168	5401
	TCGCTTGGTGCAGGTCGGGAA	bna-miR168_v2	ath-miR168a	ath-MIR168a	5113
**miR169**	CAGCCAAGGATGACTTGCCGA	bna-miR169_v1	ath-miR169a	ath-MIR169a	10010
**miR171**	TGATTGAGCCGCGCCAATATC	bna-miR171_v1	ath-miR171a	ath-MIR171a	54482
	TGATTGAGCCGCGCCAATACC	bna-miR171_v2		sly-MIR171c	33222
	TGATTGAGCCGCGTCAATATC	bna-miR171_v3	mtr-miR171b	mtr-MIR171b	16452
	TGATTGAGCCGCGTCAATACC	bna-miR171_v4		mtr-MIR171b	5107
**miR172**	AGAATCTTGATGATGCTGCAG	bna-miR172_v1	ath-miR172c	ath-MIR172c	422468
	GGAATCTTGATGATGCTGCAT	bna-miR172_v2	ath-miR172e	ath-MIR172e	9098
	AGAATCTTGATGATGATGCAG	bna-miR172_v3		aly-MIR172d	8479
	AGAATCTTGATGATGCTGCAT	bna-miR172_v4	ath-miR172a	ath-MIR172a	6876
	AGAATCTTGATGATGTTGCAG	bna-miR172_v5		ath-MIR172c	4545
	AGAATCTTGATGATGCTGCA	bna-miR172_v6	zma-miR172a	zma-MIR172a	3850
**miR319**	TTGGACTGAAGGGAGCTCCCT	bna-miR319_v1	ath-miR319a	ath-MIR319a	46441
	TTGGACTGAAGGGAGCTCCC	bna-miR319_v2	mtr-miR319	mtr-MIR319	37491
	TTGGACTGAAGGGAACTCCCT	bna-miR319_v3		vvi-MIR319f	12179
	TTGGACTGAAGGGAGCTCCTT	bna-miR319_v4	ath-miR319c	ath-MIR319c	6369
	TTGGACTGAAGGGAACTCCC	bna-miR319_v5		ptc-MIR319b	6132
	TTTGGACTGAAGGGAGCTCCT	bna-miR319_v6	vvi-miR319e	vvi-MIR319e	3347
**miR390**	AAGCTCAGGAGGGATAGCGCC	bna-miR390_v1	ath-miR390a	ath-MIR390a	13211
	CGCTGTCCATCCTGAGTTTC	bna-miR390_v2		bna-MIR390b	9543
	CGCTGTCCATCCTGAGTTTCA	bna-miR390_v3		bna-MIR390b	3655
**miR394**	TTGGCATTCTGTCCACCTCC	bna-miR394_v1	ath-miR394a	ath-MIR394a	19062
	TTTGGCATTCTGTCCACCTCC	bna-miR394_v2		bdi-MIR394	3195
**miR395**	CTGAAGTGTTTGGGGGAACTC	bna-miR395_v1	ath-miR395a	ath-MIR395a	31659
	CTGAAGTGTTTGGGGAAACTC	bna-miR395_v2		osa-MIR395t	4325
**miR396**	TTCCACAGCTTTCTTGAACTT	bna-miR396_v1	ath-miR396b	ath-MIR396b	3623
**miR397**	TCATTGAGTGCAGCGTTGATGT	bna-miR397_v1	bna-miR397a	bna-MIR397a	3500
**miR400**	TATGAGAGTATTATAAGTCAC	bna-miR400_v1	ath-miR400	ath-MIR400	10737
**miR403**	TTAGATTCACGCACAAACTCG	bna-miR403_v1	ath-miR403	ath-MIR403	36064
**miR408**	ACAGGGAACAAGCAGAGCATG	bna-miR408_v1		aly-MIR408	18138
**miR824**	TAGACCATTTGTGAGAAGGGA	bna-miR824_v1	ath-miR824	ath-MIR824	72249
	TAGACCATTTGTGAGAAGGG	bna-miR824_v2		bra-MIR824	8270
	TAGACCATTTGTGAGAAGGGAA	bna-miR824_v3		bna-MIR824	7041
	TAGACCATTTGTGAGAAAGGA	bna-miR824_v4		bol-MIR824	4663
	TAGACCATTTGTGAGTAGGGA	bna-miR824_v5		bra-MIR824	4604
	TAGACCATTTGTGAGAAGAGA	bna-miR824_v6		bra-MIR824	4267
	CCTTCTCATCGATGGTCTAGA	bna-miR824_v7	aly-miR824*	aly-MIR824	4227
**miR827**	TTAGATGACCATCAACAAATA	bna-miR827_v1		tcc-MIR827	59977
**miR845**	CGGCTCTGATACCAATTGATG	bna-miR847_v1	ath-miR845a	ath-MIR845a	4297
**miR858**	TTTCGTTGTCTGTTCGACCTT	bna-miR858_V1	ath-miR858	ath-MIR858	7316
	TTCGTTGTCTGTTCGACCTTG	bna-miR858_V2	ath-miR858b	ath-MIR858b	5218
**miR894**	CGTTTCACGTCGGGTTCACCA	bna-miR894_v1		ppt-MIR894	4474
**miR916**	GGCCTATTAGCTCAGTTGGTTAG	bna-miR916_v1		cre-MIR916	7489
**miR1885**	TACATCTTCTCCGCGGAAGCTC	bna-miR1885_v1		bra-MIR1885	13998

Sequences were compared to miRBase Release 18 for homology searches.

### Temporal expression patterns of conserved miRNAs

**Figure 4:**
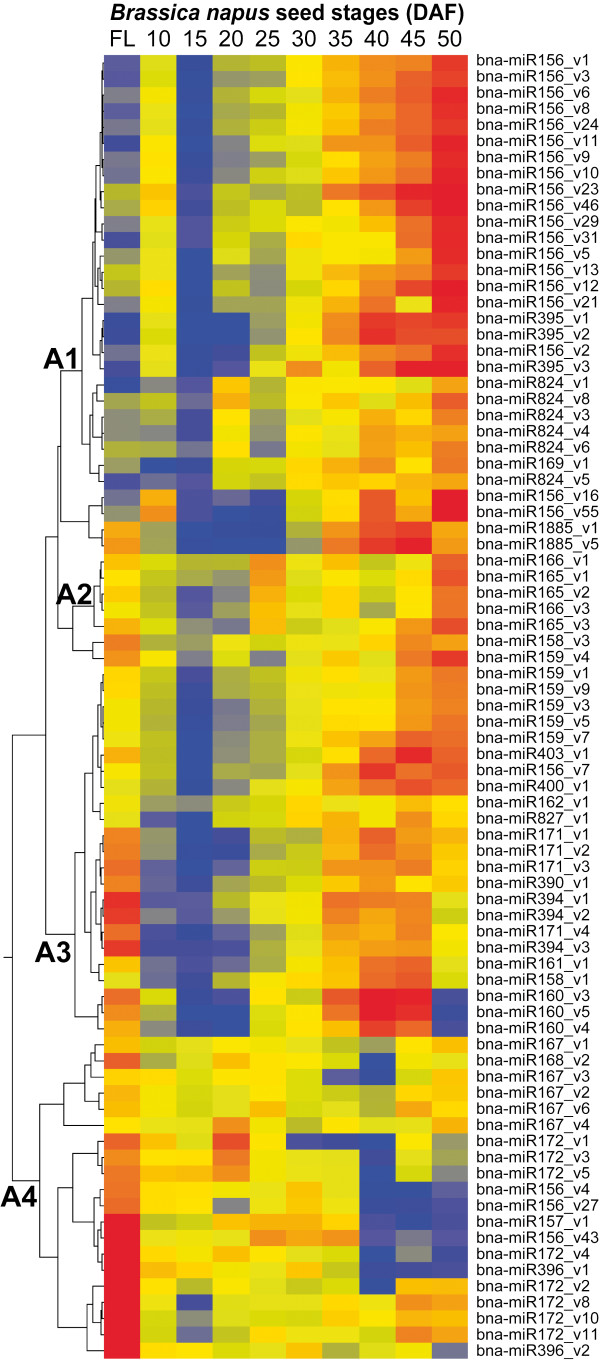
**Temporal expression patterns of conserved miRNAs and variants.** Hierarchical clustering of the 80 most abundant miRNA/variants during seed development and at flowering (FL). The color indicates the relative expression level: Blue-low, yellow-medium and red-high. Four major expression patterns (A1 – A4) are identified.

More than 500 conserved miRNA variant sequences with at least 300 reads were identified from *B. napus* seed developmental stages and flower buds. The expression profiles of these miRNA/variant sequences in flower buds, seed stages and compartments are listed in Additional file 3: Table S3. We performed hierarchical clustering analysis on the 80 most abundant miRNAs in the 10 flower bud and whole seed samples (SOLiD dataset) and Figure 4 shows the resulting expression patterns. Expression of miRNAs can be grouped into 4 major patterns, A1-A4. The majority of miRNAs (A1-A3) increased during seed maturation while a minority (A4) were preferentially expressed at early- and mid- seed development stages. miRNAs in Group A1 included most variants of miR156, miR395 and miR824 and were weakly expressed in flower buds. They showed transient expression at early seed development (10DAF) then decreased to a low level at 15DAF. After 15DAF their expression steadily increased until 50DAF. Most group A2 miRNAs such as miR159, miR158, miR166, miR400, miR403 were weakly expressed in flower buds and strongly expressed at later stages of seed development. The expression of miR166 and miR165 (group A2) were high in both flower buds and seed and had two peaks in expression at 25DAF and 50DAF. Group A3, including miR171, miR160 and miR394, were expressed in both flower buds and during seed development. Their expression increased during seed development until 45DAF and then decreased at the end of seed maturation. miRNAs in group A4, represented by miR172, miR157, miR167, miR396 and several miR156 variants, were preferentially expressed in flower buds and, to a much lesser extent, in seed developmental stages.

### Tissue-specific expression of conserved miRNAs

**Figure 5:**
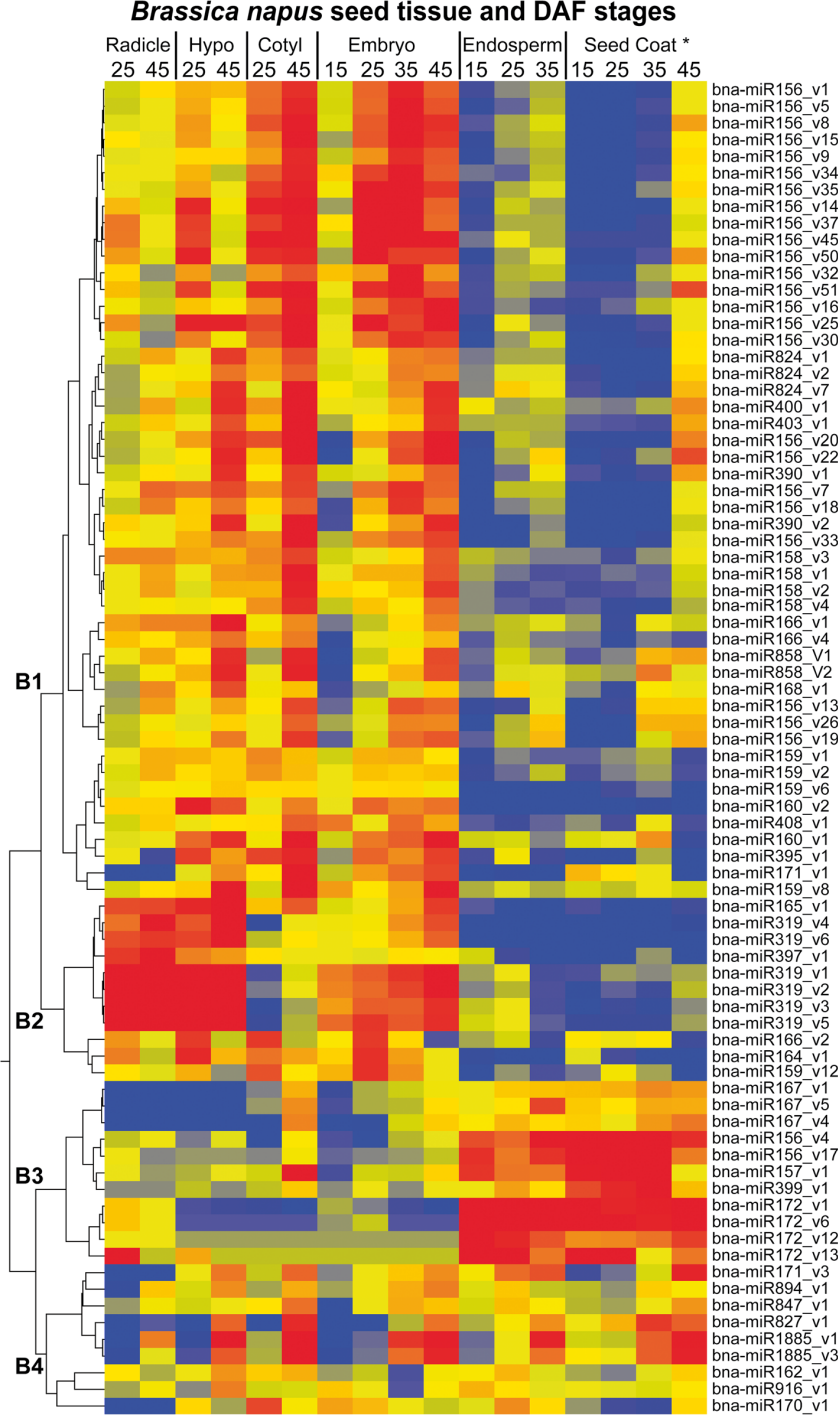
**Tissue-specific expression of most of the conserved miRNA/variants.** Hierarchical clustering of the most abundant 80 miRNA/variants in seed tissues during seed development. The color indicates the relative expression level: Blue-low, yellow-medium and red-high. Four major expression patterns (B1 – B4) were identified.

To compare the expression of miRNAs in different seed parts, hierarchical clustering analysis was performed on the normalized read counts of the top 80 miRNAs in the 17 seed part samples (Illumina dataset). The results showed strong tissue-specific expression of most of the conserved miRNAs (Figure 5). Four distinct expression patterns (B1-B4) were identified. B1 and B2 groups were preferentially expressed in embryo. miRNAs in pattern B1, which included most variants of miR156, miR158, miR824 and miR159, were preferentially expressed in the cotyledons and hypocotyl while miRNAs in pattern B2, which included miR319, miR165 and miR397 were mostly expressed in the radicle and hypocotyl. Most of the miRNAs in B3 represented by miR172, miR167and several variants of miR156/157, were endosperm and seed coat specific. miRNAs in B4 including mir827 and miR1885 seemed less tissue-specific but were preferentially expressed at later seed developmental stages. They were highly expressed in the cotyledons and hypocotyl at later stages and occasionally expressed in endosperm and/or seed coat.

Most miRNA family members showed similar expression patterns such as miR159 (A2, B1), miR319 (B2), miR172 (A3, B3), miR167 (A3, B3), miR824 (A1) and miR160 (A1). However, some miRNA families showed strikingly different expression patterns among variants. For example, most of the miR156 family (patterns B1, A1) were highly expressed in the embryo cotyledons and their expression increased steadily during seed maturation. In contrast, some miR156 variants (Groups B3, A3) showed high endosperm- and seed coat-specific expression and decreased during seed maturation. In *Arabidopsis*, many known miRNA families are expressed from precursor genes in multiple paralogous loci. This is also true for *B. napus* since several miRNA members within a family were mapped to multiple loci in the *Brassica rapa* genome [24] in our mapping analysis (see below). Members of miRNA families with divergent expression patterns are probably expressed from different paralogous loci. However, it must be noted that within each family, different loci sometimes produce identical miRNAs, therefore the abundance of a mature miRNA may not always correspond to expression of a specific precursor.

### Validation of miRNA expression by TaqMan miRNA Assay (qPCR)

**Figure 6:**
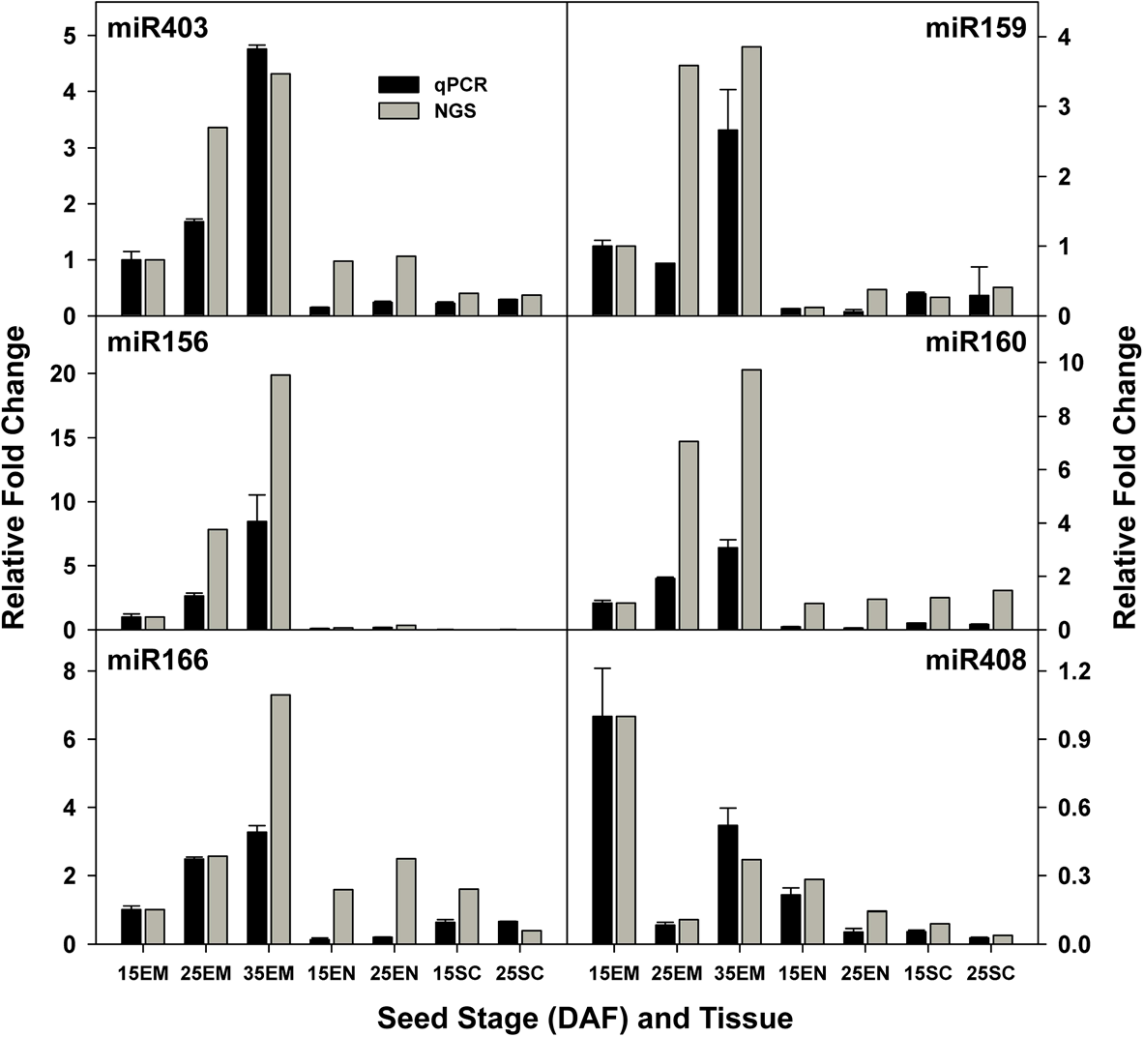
**Comparison of miRNA expression measured by qPCR (TaqMan MicroRNA Assays) and Solexa sequencing (NGS).** For 6 selected miRNAs, the miRNA expression changes at different tissues/stages were calculated relative to the embryo at 15 DAF(15EM). 15EM: embryo at 15 DAF, 25EM: embryo at 25DAF, 35EM: embryo at 35DAF, 15EN: endosperm at 15 DAF, 25EN: endosperm at 25 DAF, 15SC: seed coat at 15 DAF, 25SC: seed coat at 25 DAF.

We employed quantitative TaqMan PCR (qPCR) to validate miRNA expression determined by deep sequencing. Six moderately to highly abundant miRNAs were chosen for validation in seven tissues and stages. All of the 6 selected miRNAs were detected in all tissues and stages. As shown in Figure 6, the relative changes in miRNA expression calculated from qRT-PCR and sequencing are highly correlated (R^2^ = 0.825), indicating consistency between both approaches.

### Identification of putative precursors of *B. napus* miRNA’s using the *B. rapa* genome as a surrogate for the *B. napus* A genome

Currently, the complete *B. napus* genome sequence is not available; therefore it is difficult to identify miRNA precursor genes since weakly expressed genes are not well represented in EST databases and homeologous genes are not reliably distinguished. It is well-known that *B. rapa* contributed the A genome to the allotetraploid *B. napus* containing A and C genomes. Although the *B. napus* A genome has undoubtedly diverged from the *B. rapa* reference prior to and after polyploidization, evidence suggests that it retains *B. rapa* characteristics. For example, Cheung et al. [25] showed that “the A genomes of *B. rapa* and *B. napus* show a high degree of similarity along their entire length” and that “only a small number of homeologous recombination events have been observed in oilseed rape cultivars”. Furthermore, when aligned genome segments of *B. rapa* and the *B. napus* genome A were compared over 5 contigs, a low average SNP frequency of 1.14% in coding regions was observed and 1.45% overall (Table 11, [25]). Comparison of a specific clone between subspecies *B. rapa trilocularis* and *B. rapa pekinensis* revealed a SNP frequency of 0.63%. When the sequence of the same clone was compared between *B. rapa trilocularis* and the *B napus* A genome, the SNP rate was still a relatively low 1.66% (p1922 in [25]). The SNP frequencies suggest that the *B. napus* A genome and *B. rapa* have not diverged enough to invalidate sequence-based annotations and predictions based on a *B. rapa* reference.

**Figure 7:**
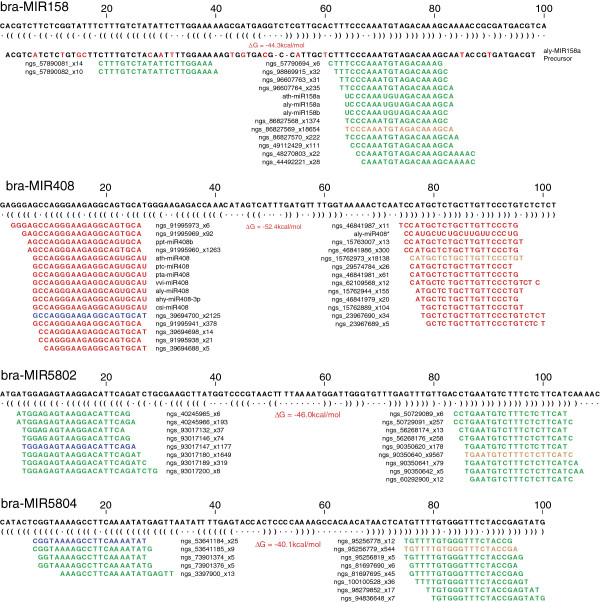
**Typical mapping patterns of miRNAs.** Sequence reads are mapped to the putative precursor regions of the *B. rapa* genome. Four examples of miRNA patterns are shown including miR158 and miR408 and two novel putative miRNAs (miR5802 and miR5804), each exhibiting two well-defined regions of alignment. The unique reads are mapped to the stem-loop sequence (the secondary structure with folding energy value is shown immediately below the genome sequence). Sequences with <5 reads are not shown. The sum of the read counts of each sequence are incorporated into the sequence name as _x read count. The green color represents the forward reads, red represents reverse reads. Mature miR sequences and miR* sequences are highlighted in orange and blue respectively.

Therefore, we analyzed the recently released *Brassica rapa* genome [24] for *B. napus* miRNA precursor sequences. We mapped the sequencing reads, known plant miRNA mature sequences and hairpin sequences from miRBase (Release18) to *B. rapa* genome sequences (v1.1), then merged the 3 mapping results to identify the conserved miRNA loci. In total, 300 plant miRNA hairpin sequences and more than 2000 mature miRNAs from miRBase (Release 18) were mapped to *B. rapa* chromosomes and scaffolds. Mapping of the 300 known plant miRNA hairpin sequences identified 128 regions of similarity to the *MIRNA* queries in the *B. rapa* genome (Additional file 4: Table S4) which covered 40 miRNA families. In most cases, there were corresponding annotated mature sequences and sequencing reads mapped to these loci and the mapping patterns of sequencing reads to the regions were very typical- as described in Kozomara and Griffiths-Jones [9] – indicating they are orthologous MIRNA loci in *B. rapa* (Additional file 5: Figure S1). The miRNA-like stem-loop secondary structures further confirmed authentic MIRNA loci. Examples of typical mapping patterns of miRNAs are shown in Figure 7. Multiple sequences, including the annotated miRNA and sequence variants with different sizes (one or two bases shorter, longer or shifted in the 5’ or 3’ direction), star sequences and variants of star sequences, were mapped to each precursor locus, on both arms of each precursor stem-loop structure. The ‘true’ miRNA sequences are presumably dominant among the sequence variants. Most of the miRNAs with stem-loop structures and typical mapping patterns are highly conserved in the plant kingdom including miR156, miR159, miR160, MiR166, miR167, miR172, miR319 and miR395 [26]. However, several regions with similarity to MIR840, MIR414, MIR415, MIR2911 and MIR2936 can be folded into stem-loop secondary structures but have no sequencing reads mapped to the genomic loci, indicating that these loci are not expressed during seed development in *B. napus* or may not be true MIRNA loci. Loci with similarity to MIR2911, MIR2906, MIR2916 and MIR1310 showed multiple offset reads distributed across the entire gene sequences. Patterns of overlapping reads covering the entire putative locus indicate they are more likely siRNA loci rather than MIRNA loci according to recent mapping guidelines in Kozomara and Griffiths-Jones [9].

Examining the mapping patterns of the annotated miRNA sequences (mature and/or mature*), conserved new miRNA sequences identified in this study, and other sequencing reads, revealed an additional 55 MIRNA loci in the *B. rapa* genome (Additional file 4: Table S4 and Additional file 5: Figure S1). No known orthologous hairpin sequences of these miRNAs were mapped to these 55 loci in *B. rapa,* however, the stem-loop structure of the flanking regions and the typical mapping patterns of sequencing reads indicated they are authentic MIRNA loci according to current guidelines [9,27]. In these cases, there is low sequence conservation in the B. rapa flanking regions outside the miRNA mature sequences. For example, the miR408 mature sequence, its variants and star sequences were cleanly mapped to the *B*. *rapa* genome (Figure 7), although the orthologous MIR408 precursors from other species do not share sequence similarity with the flanking region. Both the miRNA-like stem-loop secondary structure and the embedded nature of miRNA sequencing reads provided strong evidence that this region is an authentic MIR408 locus in *B. rapa*.

The evaluation of mapped sequence reads also provides valuable information about the relative abundance of mature sequences from different arms of stem-loops, isoforms (variants) of mature miRNA sequences, and the confidence in a given microRNA annotation. In most cases, the annotated miRNA (mostly 21nt or 20nt) was the most abundant small RNA sequence mapped to an MIRNA locus. Sometimes, the 22-25nt isoforms of the annotated miRNA were more abundant, suggesting they are more likely to be the ‘true’ (most representative) miRNA in *B. napus.* In some cases, several annotated miRNAs from different species were mapped to the same MIRNA loci. For instance, both the annotated bna-miR397a, b (3,500 reads) and ath-miR397a, b (302 reads) were mapped to MIR397a. bna-miR397a, b was substantially more abundant than ath-miR397a, b, indicating it is the ‘true’ miRNA in *B. napus* as annotated (Additional file 5: Figure S1_bra-MIR397a). Occasionally, the star sequence or a variant of the star sequence from the opposite hairpin arm of the miRNA precursor was more abundant than the annotated miRNA. For example, the miR2111* sequence was more abundant than miR2111 in bra-MIR2111a, b, c and d (Additional file 5: Figure S1), suggesting miR2111* is the true miRNA mature sequence in *B. napus*. This is also true for bra-MIR391a and bra-MIR408. The sequence of miR391* (303 reads) was more abundant than the annotated miR391 (38 reads) and a variant of miR408* (18,138 reads) is considerably more abundant than miR408 (2,125 reads) and miR408* (300 reads) (Figure 7), indicating that they are most likely the dominant mature miRNA sequences in *B. napus* and therefore are named as bna-miR391a and bna-miR408 (Additional file 5: Figure S1).

Mapping of small RNA sequencing reads and known miRNA sequences (miRBase, Release18) to the *B. rapa* genome revealed several chromosomal regions with clusters of MIRNA genes. These included a cluster containing three MIR156 genes (bra-MIR156f, k, r) on chromosome A06 within a 1000 bp region; a MIR169 cluster as well as a MIR395 cluster, also on chromosome A06. In addition, we also found that some MIRNA precursors gave rise to two or more distinct miRNA/miRNA* duplexes from different positions. As shown in Additional file 5: Figure S1, the MIR159, MIR319 and MIR169 precursor loci as well as newly identified putative novel MIRNA loci MIR5801 and MIR5810 (Additional file 6: Figure S2) can be folded into a long stem-loop structure and generated multiple distinct sRNAs.

### miRNA target prediction

miRNAs regulate gene expression through targeting transcripts for cleavage, translational repression or mRNA decay. Therefore, it is important to identify miRNA targets to understand miRNA function. In plants, all known miRNA targets have perfect or near-perfect complementary sequences to cognate miRNAs [1,28,29]. This has allowed many computational approaches to predict miRNA targets [30-33]. We used TargetFinder (Release 1.6; http://carringtonlab.org/resouces/targetfinder) [33], to search against Brassica EST unigenes (http://brassicagenomics.ca/data/brassica_ests.fasta) for the targets of miRNAs with more than 300 reads. We predicted 5,948 miRNA-target pairs including 1,783 unique *B. napus* miRNA targets (EST contigs) for 543 unique miRNAs (Additional file 7: Table S5). Among these predicted targets, 1,591 EST contigs are annotated as homologs of *Arabidopsis* genes by BLAST-based analysis of sequence homologies in TAIR9 transcripts and protein datasets, while 192 of them have no *Arabidopsis* homologs (Additional file 7: Table S5, Group 4)*.* These non-annotated EST contigs might represent genes specific to *B. napus.*

To understand the biological roles of the miRNA-target pairs in seed development, we performed miRNA-pathway analysis by mapping the *Arabidopsis* homologs of the 1591 target ESTs to Mapman pathways [34]. The functional classification based on MapMan terms using the Classification SuperViewer Tool w/Bootstrap (http://bar.utoronto.ca/ntools/cgi-bin/ntools_classification_superviewer.cgi) revealed that these homologs of target genes were highly enriched in pathways of RNA, DNA, protein, S-assimilation, development, signaling and redox (P < 0.01). Detailed inspection of the results revealed that some miRNAs function in the same pathway(s) by targeting different genes in the same family. Previous reports grouped miR156 and miR157 as one miRNA family (miR156/157) due to their high sequence homology and co-targeting to Squamosa-promoter Binding Proteins (SBP) or SBP-like proteins (SPL). Similarly, miR159/319, miR165/166 and miR170/171 were also grouped together based on shared targets [22,28]. In addition, we also predict that miR173, miR400 and miR396 "co-target" pentatricopeptide (PPR) repeat-containing proteins; miR156, miR394, miR319 "co-target" F-box family proteins and miR160, miR167, miR390 and miR156 "co-target" various auxin response factors (ARFs). Therefore, many miRNAs may function together via co-targeting to regulate functionally related genes or pathways. GO term analysis using the BINGO plugin tool in Cytoscape [35] on the *Arabidopsis* homologs of the 1591 unique target sequences (corresponding to 1158 *Arabidopsis* genes) revealed that these genes are highly overrepresented in regulation, development and stress responses (Table 3, Additional file 8: Figure S5).

**Table 3:** Enriched GO terms associated with miRNA targets (homologs in Arabidopsis)

**GO- ID**	**Description**	**corrected p-value**
9987	cellular process	5.82E-09
48856	anatomical structure development	1.12E-08
48367	shoot development	1.25E-08
22621	shoot system development	1.43E-08
48366	leaf development	2.21E-08
7275	multicellular organismal development	4.51E-08
32502	developmental process	4.51E-08
48827	phyllome development	4.51E-08
32501	multicellular organismal process	4.53E-08
44237	cellular metabolic process	5.60E-07
50896	response to stimulus	1.09E-06
65007	biological regulation	6.26E-06
48608	reproductive structure development	6.71E-06
10014	meristem initiation	7.32E-06
42221	response to chemical stimulus	7.32E-06
48532	anatomical structure arrangement	8.21E-06
3006	reproductive developmental process	8.21E-06
48513	organ development	1.13E-05
48731	system development	1.13E-05
22414	reproductive process	1.18E-05
8152	metabolic process	1.18E-05
9933	meristem structural organization	1.36E-05
10072	primary shoot apical meristem specification	1.93E-05
3	reproduction	2.37E-05
50789	regulation of biological process	2.55E-05
9791	post-embryonic development	2.75E-05
50793	regulation of developmental process	8.12E-05
19222	regulation of metabolic process	1.80E-04
31323	regulation of cellular metabolic process	2.61E-04
6950	response to stress	3.41E-04
10468	regulation of gene expression	3.83E-04
50794	regulation of cellular process	3.92E-04
60255	regulation of macromolecule metabolic process	3.92E-04
48507	meristem development	3.92E-04
10016	shoot morphogenesis	3.93E-04
48508	embryonic meristem development	4.30E-04
44281	small molecule metabolic process	4.40E-04
80090	regulation of primary metabolic process	5.20E-04
45449	regulation of transcription	5.20E-04
19219	regulation of nucleobase, nucleoside, nucleotide and nucleic acid metabolic process	5.20E-04
9653	anatomical structure morphogenesis	5.20E-04
10035	response to inorganic substance	5.55E-04
9889	regulation of biosynthetic process	6.72E-04
31326	regulation of cellular biosynthetic process	6.72E-04
51171	regulation of nitrogen compound metabolic process	7.47E-04
10154	fruit development	7.77E-04
9628	response to abiotic stimulus	9.42E-04
6457	protein folding	9.42E-04
44238	primary metabolic process	9.48E-04
51239	regulation of multicellular organismal process	1.04E-03
10556	regulation of macromolecule biosynthetic process	1.04E-03
48316	seed development	1.25E-03
40034	regulation of development, heterochronic	1.38E-03
6725	cellular aromatic compound metabolic process	1.45E-03
6519	cellular amino acid and derivative metabolic process	4.16E-03
10033	response to organic substance	4.38E-03
6970	response to osmotic stress	5.63E-03
9793	embryonic development ending in seed dormancy	5.75E-03
51716	cellular response to stimulus	6.12E-03
19752	carboxylic acid metabolic process	6.12E-03
43436	oxoacid metabolic process	6.12E-03
6082	organic acid metabolic process	6.24E-03
44283	small molecule biosynthetic process	6.36E-03
9855	determination of bilateral symmetry	6.50E-03
51252	regulation of RNA metabolic process	7.42E-03
42180	cellular ketone metabolic process	8.00E-03
9408	response to heat	8.53E-03

In *Arabidopsis*, a majority of miRNA targets have been identified and experimentally validated. Many homologs of these *Arabidopsis* miRNA target genes can be found from the predicted targets, confirming that conserved miRNAs also have conserved targets across species and likely have similar functions in *B. napus* and *Arabidopsis*. This is consistent with previous studies in other species [22,36]. In addition to the conserved targets, we also predicted new targets for the conserved *B. napus* miRNAs. For example, in addition to members of the *SPL* gene family (*SPL2*, *SPL3*, *SPL6*, *SPL10* and *SPL15*), we also predicted other new miR156 targets, such as homologs of *Arabidopsis* vacuolar H^(+)^-ATPases (*AVA-P3*), *LOW CELL DENSITY1 (LCD1*), an F-box family protein (AT1g51550), a leucine-rich repeat transmembrane protein kinase (At4g23740), an auxin-responsive GH3 family protein (At2G37860) and *FUSCA3* (*FUS3*), which is a key regulator of seed development [37]. Individual miR156 variants may have specific additional targets such as the homolog of *SEC14* cytosolic factor (At4g39170) specific for bna-miR156_v1, an ARID/BRIGHT DNA-binding domain-containing protein (*ARID*) specific for bna-miR156_v21, *FUS3* and an ENTH domain-containing protein (At2G01600) specific for bna-miR156_v54, an isoflavone reductase (AT1g19540) and an isocitrate lyase (AT3G21920) specific for bna-miR156_v17 and *BRASSINOSTEROID INSENSITIVE 1* (*BRI1*) for bna-miR156_v5 and bna-miR156_v7. This suggests the possibility that individual miRNA variants have different functions through their specific targets.

### Expression of putative conserved miRNA targets

**Figure 8:**
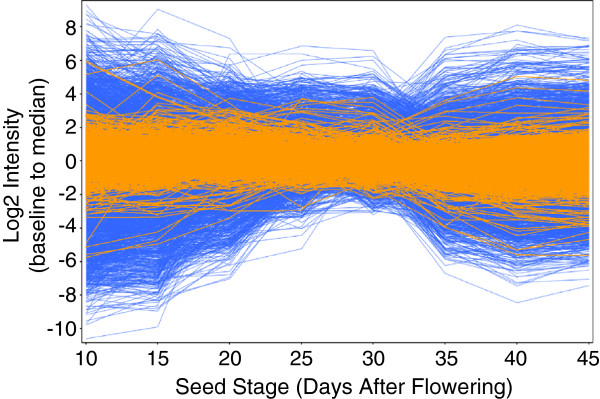
**Overview of gene expression during seed development (in blue).** The predicted miRNA targets are highlighted in orange. Each line represents one unigene. The Y-axis shows the normalized signal intensity (with baseline transformation to the median of all samples).

To compare the expression of the miRNAs with that of their targets, we performed global transcript profiling using *Brassica* 90K Combimatrix arrays (http://www.brassicagenomics.ca/90koligoarray.html). The expression of miRNA target genes in seed development was extracted from the microarray results and is listed in Additional file 7: Table S5. The overview of the expression of *B. napus* genes (based on a set of 90K Brassica EST contigs) is shown in Figure 8. The expression changes of miRNA targets (highlighted in orange) were notably smaller than overall changes in gene expression. Detailed comparison of the expression patterns of miRNAs and their targets at the corresponding time points (10-45DAF) revealed that, although some miRNA-target pairs showed the expected negative (inverse) correlation, many others showed either no correlation or even displayed a positive correlation (Additional file 7: Table S5). Most miRNAs were expressed at low levels (Additional file 7: Table S5, Group 3). Similarly, many of the miRNA target genes were also expressed at relatively low levels during seed development and maturation (Additional file 7: Table S5, Group 2). The maximum raw signal intensities of genes in Group 2 were under 500 throughout seed development, indicating that the expression of these target genes might be repressed throughout seed development. Homologous EST contigs of many known miRNA target genes including most *SPLs* (targets of miR156), *MYB101* (target of miR159), *ARF6* and *ARF8* (targets of miR167) and numerous others either showed negative expression patterns or were expressed at very low levels throughout seed maturation.

### Identification of putative novel miRNAs, precursors and targets

Many miRNA families are highly conserved in evolution [22]. However, there are also species-specific, recently evolved miRNA genes [38,39]. To identify miRNAs specific to *Brassica*, we again used the *B. rapa* genome (V1.1) since the complete *B. napus* genome is not available. All of the sequences with read counts >300 that were not annotated as tRNA, rRNA, snoRNA, miRNA, repeat or cDNAs, but mapped to the *B. rapa* genome were used to detect potentially novel miRNA genes. The flanking genomic sequences of the mapped reads were extracted and searched for regions that could be folded into a stable miRNA precursor-like hairpin with strong pairing between the mature miRNA and miRNA* sequence on the opposite hairpin arm. This analysis employed NOVOMIR, a program for the identification of plant miRNA genes [40]. We identified 78 putative novel miRNA sequences which mapped to 578 genomic loci in *B. rapa*. To eliminate siRNAs from repeat sequence classes and ensure that they were from good quality single-stranded hairpins, reads that mapped more than 10 times to the reference genome or mapped to a bidirectional small RNA cluster were also discarded. The mapping patterns of sequence reads in each genomic locus were manually examined and the loci that didn't meet the consensus set of guidelines for miRNA annotation [9] were filtered out. Loci with an embedded known miRNA were identified as conserved miRNAs and therefore also eliminated. Following the above filtering, we identified 10 putative novel MIRNA loci for 13 out of the 78 presumed miRNA sequences and several conserved miRNA variants including miR400, miR169 and miR395. These putative novel miRNA sequences and their hairpin structures, as well as the mapping patterns of sequencing reads in the hairpin regions, are presented in Additional file 9: Table S6 and Additional file 6: Figure S2. Several putative miRNA sequences were mapped to the same loci. For example, 3 putative novel miRNA sequences were embedded in the MIR5802; of these ngs_90350641_x9567 is the most abundant sequence and most likely the true miRNA mature sequence (miR5802) and ngs_93017147_x1177 is the corresponding star sequence (miR5802*) while ngs_93017180_x1647 is a variant of the same miRNA* (Additional file 6: Figure S2). Previous studies indicated that non-conserved miRNAs are usually weakly expressed with a tissue-specific pattern [41]. Due to the unavailability of *B. napus* genome sequences (or of a C genome reference) and the fact that the small RNA libraries were mostly constructed from seeds, miRNAs identified in this study might represent only a fraction of the total number of novel miRNAs in *B. napus*.

We also predicted targets for the novel miRNA candidates using TargetFinder software (R1.6). In total we predicted 64 *Brassica* contigs as targets of the 10 putative miRNAs (Additional file 10: Table S7). Based on the annotations of their *Arabidopsis* homologs, the functions of these predicted novel miRNA targets are involved in many pathways including development and signaling. miR5801 was the most abundant potential miRNA (57,087 total reads) among all predicted novel miRNAs. It is preferentially expressed in embryo, especially in the cotyledons, and increased substantially during seed maturation. It was predicted to target homologs of *Arabidopsis DEMETER* (*DME*) and AT1G16900 (sugar binding/transferase). The expression of these two genes reciprocally decreased during seed maturation. miR5807 (20,078 reads), the second most abundant potential miRNA, accumulated in different compartments of the seed during seed maturation. It was predicted to target EST contigs homologous to an Arabidopsis pentatricopeptide (PPR) repeat-containing protein and a sphere organelles protein-related gene. The expression of the target PPR repeat-containing protein showed consistently low expression from 10 to 45 DAF in seed.

## Discussion

### Increase in the number of identified miRNAs in *B. napus and B. rapa*

miRNAs are essential regulators of gene expression in all plants and animals. According to the miRBase database (Release18), 291 *Arabidopsis* and 581 rice miRNAs have been identified respectively. *B. napus* is polyploid with a much larger and more complex genome than *Arabidopsis* and rice. However, there were only 46 miRNAs (27 unique sequences) annotated in miRBase (Release 18). In this study, we identified more than 500 miRNAs (unique sequences) including 27 known *B. napus* miRNA sequences already in miRBase and 10 putative novel miRNAs. The miRNA-like hairpin structures of the flanking sequences and the typical mapping patterns of the NGS reads to the precursor regions (Additional file 5: Figures S1 and Additional file 6: Figure S2) provided supporting evidence for the newly discovered miRNAs.

In the absence of a *B. napus* genome sequence, we used the newly released *B. rapa* draft genome sequence (v1.1) for identification of the hairpin precursor sequences of the newly identified miRNAs. This led to the discovery and annotation of more than 180 miRNA hairpin sequences and loci. To date, there are only 19 miRNAs and their hairpin precursor sequences from *B. rapa* deposited in miRBase (Release 18). Identification of the MIRNA genes in the *B. rapa* genome has significantly enriched the repertoire of *Brassica* MIRNA genes and improved the annotation of the newly sequenced *B. rapa* genome.

In a report published while this article was in preparation, Zhao et al. [14] analyzed small RNA expression profiles of *B. napus* siliques at early embryonic developmental stages (3,7,14 and 21 DAF) in two cultivars with differing oil contents. They identified 50 conserved miRNAs of which 36 were identical to and 14 highly homologous to known miRNAs in miRBase. In addition, they predicted 11 new miRNAs. This study provides a more comprehensive temporal and spatial profiling of miRNAs focused on post-embryonic development and seed maturation. By employing greater sequencing depth, we identified many more miRNAs including conserved and novel putative miRNAs with high confidence and investigated their specific expression patterns during seed development. To provide a more complete picture of miRNAs in *B. napus* seed development we provide a detailed comparison of our data with that of Zhao et al. [14] (Additional file 11: Table S8). We also identified almost all of the conserved miRNAs that they reported. For the 11 predicted new miRNAs, we identified the corresponding precursor loci in the *B. rapa* genome and examined the patterns of sequences that mapped to each region (Additional file 12: Figure S3). Among the 11 new miRNAs predicted by Zhao et al. [14] bna-miR2203, bna-miR2204 and bna-miR2207 were also predicted in this study and bna-miR2202 was predicted to be a star sequence. We did not detect reads corresponding to the exact sequences of bna-miR2206a, b (these two sequences are identical), bna-miR2211 and bna-miR2216. The sequences of bna-miR2205, bna-miR2206a, b and bna-miR2225 occurred in the *B. rapa* genome 30, 37 and 24 times respectively, suggesting that they are unlikely to be authentic miRNAs. The observed pattern of overlapping reads mapping to a broad region of the flanking sequences of miR2205, miR2206 and miR2225 indicates that they are more likely siRNA loci (Additional file 12: Figure S3). The number of sequence reads for bna-miR2209 (5 counts) and bna-miR2220 (32 counts) were low and therefore excluded from our prediction.

Due to the inconsistency of miRNA names in Zhao et al. [14] and miRBase, (miR-2202 to miR-2225 were annotated to other species in miRBase, and the sequence of bna-miR1114 in their paper is different from the annotated bna-miR1114 sequence in miRBase) we retain our temporary miRNA names for all miRNA/variants with reads >300 in each family according to their abundance. These names are subjected to change when the *B. napus* whole genome sequence becomes available.

Very recently, Xu et al. identified 41 conserved and 62 Brassica-specific candidate *B. napus* miRNAs from pooled *B. napus* tissues using *B. napus*, *B. rapa* and *B. oleracea* genomic survey sequences (GSS) databases and ESTs as references [16]. We detected 95 out of the 99 available sequences they identified, of which 48 had >300 total reads, mostly conserved miRNAs although a few of them are actually the same as the new conserved or putative novel miRNAs we identified (Additional file 13: Table S9). For instance, the sequence of Bna-miRC17a-1 is identical to our new conserved bna-miR158_v1 (379,019 reads), the sequence of Bna-miRC25 is annotated as bna-miR319_v3 (12179 reads) and Bna-miRC2 is the same as our predicted miR5807a/b (20,078 reads). The remaining 55 sequences with no reads or total reads less than 300 were excluded from our analysis. Zhou et al. [15] identified 84 conserved and non-conserved miRNAs (belonging to 37 miRNA families) from Cd-treated and non-treated *B. napus* seedlings using the *B. napus* cDNA database and tentative consensus sequences of the *B. napus* Gene Index, leaving 1,731 miRNA homologues with high similarity to miRNAs from other species (with no more than two nucleotide mismatches) not validated due to lack of a reference sequence. Comparison of the expression of the 90 known miRNAs in Zhou et al. [15] and this study revealed many commonly expressed miRNAs despite the very different conditions and stages, especially for the most highly expressed miRNAs such as miR156, miR167, miR158 and miR166. However, 23 of the 90 previously annotated miRNAs were not expressed under any tissues/stages/conditions in either study (no expression in seed stages, flower bud, seedling, root shoot and Cd treatment), indicating that some of the "known" miRNAs are likely not true miRNAs (Additional file 14: Table S10). In this study, we identified many more miRNAs with a much more stringent cutoff at >300 reads using the *B. rapa* genome as reference. With our deep sequencing data, we were able to follow the latest microRNA annotation guidelines [9] to integrate sequencing data with MIRNA annotation and exclude many false MIRNA loci which are more likely siRNA loci based on read mapping patterns. Many of the miRNAs identified in this study are now validated by recent reports (Additional file 11: Table S8, Additional file 13: Table S9 and Additional file 14: Table S10) [14-16].

### The most abundant miRNA in seeds of *B. napus* relative to other species

miR156 (ath-miR156a) is the most abundant miRNA in *B. napus* seeds, accounting for more than 48% of total miRNA reads. In monocotyledonous plants, miR168a was reported as the most abundant miRNA in rice [42-44], *Brachypodium distachyon*[45], Barley [46] and wheat [47] while miR156 is the second most abundant miRNA in these species. Our data showed miR156 (ath-miR156a) to be preferentially expressed in the cotyledons of embryo and miR168 (ath-miR168a) to be preferentially expressed in endosperm at a much lower level (than miR156). But miR168 is the most abundant miRNA not only in monocot seeds (e.g. developing rice grains) but also in other monocot tissues (e.g. seedlings). Therefore the extraordinarily high level of miR168 in monocots is not due to the larger portion of endosperm in grass seeds but represents a significant regulatory difference between monocots and dicots. It has been reported that miR168 targets *ARGONAUTE1* (*AGO1*) in *Arabidopsis*, rice, and wheat. *AGO1* is involved in miRNA biogenesis and miR168-mediated regulation of *AGO1* mRNA has been reported to be important for proper plant development [48]. It will be interesting to further compare the regulation of miR168-*AGO1* in monocots and dicots as more data becomes available.

### miRNA targets

Various prediction approaches have been applied successfully for the identification of miRNA targets in plants [30-33]. In *B. napus*, Xie et al. [11] predicted 67 potential target genes for 21 newly identified miRNAs. Huang et al. [49] identified several miR395 target genes. Since few miRNAs targets have been identified in *B. napus*, we may assume that the majority of miRNA targets have not been discovered. In this study, we predicted 5,948 miRNA-target pairs including 1,783 unique *B. napus* miRNA targets (EST contigs) for 543 unique miRNAs using TargetFinder (Release 1.6). Very recently, Xu et al. [16] experimentally identified 33 non-redundant mRNA targets of 17 conserved Brassica miRNAs and 19 new non-redundant mRNA targets of novel Brassica-specific miRNAs by sequencing the mRNA degradome. Zhou et al. [15] identified 802 targets for 37 miRNA families by deep sequencing of four degradome libraries from Cd-treated and control (untreated) roots and shoots of *B. napus* seedlings. Comparing our putative targets to Xu et al. [16] and Zhou et al. [15], we found significant overlap among them based on the annotations of target genes, including miR156 targets (SPLs), miR167 targets (ARFs), miR159 targets (MYBs), miR169 targets (NF-Y subunits), miR164 targets (NAC-domain proteins) and miR172 targets (AP2-like transcription factors). Detailed comparison revealed that 78 out of the 200 category I miRNA targets from Zhou et al. [15] were also predicted as targets of the same miRNA families in this study (Additional file 15: Table S11). miRNAs with reads < 300 counts such as miR860 and miR414 were excluded from our target prediction. This might explain some of the differences between two studies. In addition, different *B. napus* EST datasets were used in the two studies which might also contribute to differences.

### Expression patterns of miRNA’s and their putative targets

A typical miRNA acts to downregulate expression of its target gene by directing cleavage of its highly complementary target transcripts. This implies that expression of miRNAs and their targets should be negatively correlated. However, as others have noted [15,50], the expected inverse correlation was often not observed. In this study, we found that the top 5 most abundant miRNAs represented 85% of all miRNA sequence reads and that most miRNAs were expressed at low levels (Additional file 3: Table S3). Similarly, many of the miRNA target genes were expressed at relatively low levels during seed development and maturation. For example, miR156 expression increased substantially but *SPLs* (known targets of miR156) were expressed at low levels from 10 to 50 DAF (Additional file 16: Figure S4A & B). In *Arabidopsis*, the expression of *SPLs* significantly decreased from the globular to heart stage of seed development and remained low throughout the rest of seed development (Additional file 16: Figure S4C). Thus, in *B. napus,* it is likely that expression of *SPLs* was already suppressed by miR156 before 10DAF.

We also observed that typically, the range of expression changes exhibited by the miRNA targets during seed development were smaller than the overall range of gene expression changes (Figure 8). This observation is consistent with previous reports that miRNA-mediated post-transcriptional regulation might contribute to buffering changes in target gene expression by establishing a threshold for target-triggered regulatory effects within a complex regulatory network [51,52]. The buffering function of miRNA might be critical to prevent the fluctuation of target gene expression levels and therefore prevent aberrant development events. The occurrence of miRNA multiple targeting and co-targeting also complicates the relationship between miRNA and their targets. Given the complexity of gene regulatory networks, an individual miRNA target might also be regulated by various factors in addition to the cognate miRNAs and one of the other factors may be predominant in a given tissue or stage. In addition, recent studies have shown that plant miRNAs can repress target genes at the level of translation with little or no influence on the mRNA abundance [6].

### The roles of miRNAs in seed development and maturation

Plant miRNAs are important for fine-tuning plant development including organ identity, patterning, polarity, developmental phase transitions and development of cellular organelles. Previous reports suggested the potential involvement of miR156, miR160, miR169, and miR396 in seed development, dormancy, and germination [53]. However, the specific functions of miRNAs in seed maturation are largely unknown.

miR156, miR172 and their targets (SPLs and AP2-like transcription factors) are key players in coordinating plant phase transitions, from juvenile to adult and from the vegetative to the reproductive phase, during post-embryonic development [54]. In *Arabidopsis* and Maize, miR156 maintains the juvenile phase and prevents precocious flowering while miR172 acts downstream of miR156 and promotes flowering by repressing *APETALA* 2-like repressors of *FLOWERING LOCUST* (*FT*). miR156 targets and represses the expression of *SPL* genes that are positive regulators of miR172. The levels of miR156 and miR172 exhibit contrasting age/development-specific expression patterns: miR156 levels decline during vegetative development whereas miR172 levels correspondingly increase [55,56].

As noted earlier, the miR156 family is the most abundant miRNA in seed development. ath-miR156a, the most abundant variant of the miR156 family, is preferentially expressed in embryo and increased 113-fold from 15DAF to 50DAF. ath-miR172c, the most abundant variant of the miR172 family, was preferentially expressed in endosperm in early seed development and decreased substantially during subsequent maturation, showing the opposite expression pattern to miR156. The complementary tissue- and stage-specific expression patterns of miR156 and miR172 are analogous to the contrasting age/development-specific expression patterns of miR156 and miR172 during the transition from the vegetative phase to flowering. This suggests that indirect repression of miR172 by the highly expressed miR156 is important for embryo maturation and that the miR156-SPLs-miR172 regulatory cascade also plays an important role during seed development and germination. Nodine and Bartel [57] proposed that miRNAs enable proper embryonic patterning by preventing precocious expression of genes normally expressed during seed maturation, partially through miR156-mediated repression of *SPL* transcripts (*SPL10* and *SPL11*). However, the substantial increase of miR156 during seed maturation and very low expression of the target *SPL* genes in late seed development indicate that the miR156-*SPL* regulatory cascade is not likely to suppress the expression of maturation genes. Indeed it seems intuitively more likely that miR156 represses floral identity (and associated zygote differentiation) in order to allow vegetative identity to become established during maturation.

The plant life-cycle is characterized by two major developmental phase transitions: germination (from seed to seedling) and flowering (from vegetative to generative growth). Recent studies indicate that genes involved in flowering and other phase transitions also regulate the transition from dormancy to germination, suggesting that conserved mechanisms control all plant phase transitions. A study of global transcriptional interactions (SeedNet) revealed coordinated regulation of plant cellular phase transitions in seed germination. An intermediate transition phase between dormancy and germination is enriched with genes involved in cellular phase transitions such as *EARLY BOLTING IN SHORT DAYS* (*EBS)*, *FLOWERING LOCUS C* (*FLC)*, *FLOWERING LOCUS T* (*FT)* and *ABSCISIC ACID INSENSITIVE 3* (*ABI3)*[58]. The hyper-abundance of miR156 in mature seeds and the presence of common regulators of phase changes in seeds and flowering suggest that the miR156-SPL regulatory cascade might control the developmental phase transition from embryonic to seedling stage, analogous to shoot maturation and the transition from the vegetative state to flowering [55,59]. High levels of miR156 and reduced SPLs and miR172 in the mature embryo may repress the developmental transition and keep seeds in the maturation/dormant state.

ath-miR159 is the second most abundant miRNA during seed development and maturation. The expression pattern of miR159 is very similar to that of ath-miR156. In a study by Reyes and Chua, miR159 was induced by ABA via the transcription factor *ABI3* and cleaves MYB DOMAIN PROTEIN 101 (*MYB101*) and MYB DOMAIN PROTEIN 33 (*MYB33*) transcripts, which were described as positive regulators of ABA signaling during germination [60]. However, this study is inconsistent with other papers that provide evidence that *GAMB-like* genes *MYB33*, *MYB101 and* MYB DOMAIN PROTEIN 65 (*MYB65*) mediate GA effects. For example, miR159 was implicated in floral and anther development by targeting the expression of *MYB33* and *MYB65*, which were shown to be involved in GA-promoted activation of *LEAFY*[61]. Recently, Alonso-Peral et al. [62] demonstrated that in miR159 mutants (mir159ab), deregulation of *MYB33* and *MYB65* in vegetative tissues resulted in up-regulation of genes that are highly expressed in the aleurone and induced by GA during germination, suggesting that miR159 acts as a molecular switch confining the expression of *MYB33* and *MYB65* to anthers and seeds. Our data shows that miR159 is preferentially expressed in the embryo, presumably allowing expression of *MYB33* and *MYB65* in the endosperm to facilitate storage product hydrolysis and later Programmed Cell Death (PCD) (both processes associated with GA action). Furthermore, within the embryo, miR159 is preferentially expressed in the cotyledons and hypocotyl, perhaps repressing specific GA processes to allow ABA-associated maturation (processes such as storage product accumulation and desiccation) to be maximized. ABA regulates many aspects of plant seed development such as seed desiccation tolerance, dormancy, and the inhibition of the phase transitions from embryonic to germinative growth [63]. GAs are antagonists of ABA and release seed dormancy, promote germination and induce flowering [64]. Taken together, we speculate that miR159 restricts specific GA effects in the embryo, primarily affecting cotyledon and hypocotyl tissues.

Several miRNAs that target components of the auxin response were identified during seed development. miR160 and miR167 are predicted to target AUXIN RESPONSE FACTORs (ARFs), specifically *ARF17*, *ARF8* and *ARF6*. miR164 and miR165/166 are predicted to target transcription factors NAC and HD-Zip respectively, which also play an important role in early auxin response [65]. Our data showed very specific expression patterns of these auxin responsive miRNAs. miR167 is one of the most abundant miRNAs during seed development and is preferentially expressed in the seed coat and endosperm. There are two peaks of miR167 reads at 25 and 40 DAF and expression of miR167 was negatively correlated with that of its targets *ARF6* and *ARF8*. miR160 was preferentially expressed in the embryo cotyledons and miR165/166 was preferentially expressed in the hypocotyl. miR164 was preferentially expressed in early embryo development (before 25DAF). The expression of the miRNA targets *ARF17*, *PHV* and *NAC1* were negatively correlated with expression of the corresponding miRNAs. The miR166/165 group and its target genes have been reported to regulate apical and lateral meristem formation, leaf polarity, and vascular development [66]. The key role of auxin in plant embryogenesis has been well described [67,68] and a proper distribution and activity of auxin are crucial for embryo development. The high abundance and specific expression of miR165/166, miR167, miR160 and miR164 indicate that these miRNAs play important roles in maintaining proper auxin signaling homeostasis in seed development.

One of the predicted novel miRNA, miR5801, also the most abundant putative miRNA sequence with 57,087 total reads, was predicted to target homologs of *Arabidopsis DEMETER* (*DME*). DME and DEM-LIKE (DML) proteins are required for appropriate distribution of DNA methylation marks, endosperm gene imprinting and seed viability in *Arabidopsis*[69,70]. Recently, Kim et al. [71] reported that miR402 affects seed germination of *Arabidopsis thaliana* under stress conditions via targeting *DEMETER-LIKE* protein3 mRNA (*DML3*). It is hypothesized that the induction of miR402 by stress guides cleavage of *DML3*, which in turn maintains DNA methylation in genes that play a negative role in seed germination. Lu et al. [72] found both methylation and demethylation events are detected during seed germination in *B. napus*. Our data showed high level of miR5801 and low level of *DME* in developing and mature seeds, suggesting that miRNAs contribute to the epigenetic regulation of genes during seed development, maturation and germination through regulation of *DME* or *DME-like* genes.

## Conclusions

Large numbers of miRNAs with diverse expression patterns, multiple-targeting and co-targeting of many miRNAs, and complex relationships between expression of miRNAs and targets were identified in this study. Development is regulated by complex networks consisting mainly of interactions between TFs, microRNAs and hormones. The relative abundance as well as specific temporal and spatial expression patterns of these miRNAs and their targets suggested that miR156, miR159, miR172, miR167, miR158 and miR166 are the major contributors to the network controlling seed development and maturation through their pivotal roles in plant development. Our data suggests a possible role for miRNA156 in regulating the phase transition from seed (embryonic) to vegetative state (germination) that is analogous to its role in the vegetative to floral transition. Altogether, the large diversity of conserved and novel miRNAs identified in *B. napus* seed development, provide new perspectives on the regulation of gene expression networks and on developmental timing by miRNAs during seed development and maturation.

## Methods

### Plant growth and material collection

Plants of *B. napus* L. cv.DH12075 were grown in 6-in pots containing soil mixed with a slow-release fertilizer 14-14-14 in a growth cabinet with a 16 h/8 h day/night photoperiod, light intensity of 250 μmol m^2^ s^-1^, and day/night temperatures of 22°C/18°C. Individual flowers were tagged on the day of flower opening. Seeds of 10, 15, 20, 25, 30, 35, 40, 45 and 50 DAF were dissected from siliques, immediately frozen in liquid nitrogen and stored at −80°C. For seed compartments, fresh seeds were manually dissected into radical, hypocotyl, cotyledon, embryo, endosperm and seed coat in RNAlater (Life Technologies) and kept at 4°C for a few days for the solution to penetrate into the seeds and then stored in −20°C. Seeds with the same DAF from different plants and different positions in the same plant were pooled together to minimize the sample variation. Only seeds from the main raceme were sampled.

### Small RNA extraction and library preparation

Small RNAs were obtained from *B. napus,* whole seeds at 10, 15, 20, 30, 35, 40, 45 and 50DAF; seed parts consisting of embryo, endosperm, and seed coat at 15, 25, 35 and 45DAF, and the embryo tissues, radical, hypocotyl and cotyledon at 25 and 45 DAF were collected. RNAs were extracted and small RNAs were enriched from all samples with the mirVana miRNA isolation kit (Life Technologies) in combination with Plant RNA Isolation Aid (Life Technologies) according to the manufacturer’s protocols. Briefly, about 10–50 mg of whole seeds or seed parts from each stage were ground into powder in liquid nitrogen and then homogenized in a mixture of the lysis solution from the mirVana miRNA isolation kit and Plant RNA Isolation Aid. The preparation was clarified by centrifugation to remove insoluble materials. Small RNAs were then purified from the lysate according to the protocol for small RNAs enrichment. The quality and the quantity of small RNAs were measured by BioAnalyzer 2100 (Agilent Technologies) with the Agilent Small RNA kit according to the manufacturer's instructions.

For sequencing using the Applied Biosystems SOLiD system (read length 35 nt), 10 libraries from small RNAs of whole seeds at 10, 15, 20, 25, 30, 35, 40, 45 and 50 DAF plus flower buds were prepared by the Small RNA Expression Kit (SREK, Applied Biosystems) with a unique “barcode” to each library. The libraries were quantified and equally pooled into one sample. The mixed sample was sequenced on a single slide using the SOLiD v3 sequencing system (Applied Biosystems) at The Centre for Applied Genomics, The Hospital for Sick Children (Toronto, Canada).

For sequencing using the Illumina Sequencing by Synthesis system (read length 36 nt), 17 libraries were prepared from small RNAs of seed parts including radical, hypocotyls, cotyledon, embryo, endosperm and seed coat at 15, 25, 35 and 45 DAF using Illumina Small RNA Sample Prep Kit v1.5 following the manufacturer's instructions. The libraries were quantified and loaded on individual lanes of a flow-cell and sequenced on a Genome Analyzer II for 36 cycles following the manufacturer's protocols (Illumina).

### Small RNA data analysis

FASTX-ToolKit (http://hannonlab.cshl.edu/fastx_toolkit/index.html) was used to pre-process the data. The sequence reads from 454 and SOLiD datasets were assigned to corresponding samples (libraries) based on specific barcode sequences added to the small RNAs during sample preparation. Sequences of poor quality or consisting only of adaptor nucleotides were removed. The sequencing adaptors were trimmed and reads longer than 18nts were retained for further analysis. The processed sequences were searched against the RFam database [73,74] for tRNA/rRNA/snoRNA-derived sequences. The remaining sequences were mapped against the miRBase database (Release 18) to search for conserved miRNAs. The number of sequence reads for each miRNA was normalized to 10 M total reads/library for both SOLiD and Illumina datasets based on the sequencing depth. The normalized read counts were used to determine the expression level for each miRNA isoform/variant in relation to time (SOLiD dataset) and space (Illumina dataset) during seed development. The normalized read counts of miRNAs from both datasets were imported into and processed in GeneSpring GX11. The miRNA expression pattern analysis was conducted by Pearson correlation hierarchical clustering with complete linkage rule in GeneSpring GX11.5.1.

### Mapping of small RNA sequences to genome and visualization

The unique sRNA sequences pooled from SOLID and Illumina datasets were mapped to *Brassica rapa* genomic scaffolds using Bowtie [75]. CLC genomics workbench 4.5 (CLC Bio, http://www.clcbio.com) was used to visualize the mapping patterns of small RNA reads to *Brassica rapa* chromosomes and genomic scaffolds.

To identify the conserved miRNA precursor loci, we extracted all known plant miRNA mature sequences and hairpin sequences from miRBase (Release 18) and mapped them to *Brassica rapa* genome (V1.1) using CLC genomics workbench software. The sequencing reads were also mapped to the same reference genome with Bowtie [75]. To improve the visualization, we only included unique sequences with read count ≥5. A new mapping file was created by merging three mapping results and visualized in CLC genomics workbench. The mapping pattern and the secondary structure of each potential MIRNA locus were examined manually. Annotations were added to each locus when both the secondary hairpin structure and the expression pattern meet the criteria of miRNA annotation according to guidelines in Kozomara and Griffiths-Jones [9].

### Novel miRNA identification

We extracted 200 bp upstream and downstream genomic sequences (*B. rapa*) for all the sequences that perfectly aligned to the *B. rapa* genome and had more than 300 total reads. We further predicted the hairpin-like RNA secondary structures using NOVOMIR [40] according to criteria described in Meyers et al. [27]. NOVOMIR uses a series of filtering steps and a statistical model to discriminate a pre-miRNA from other RNAs. NOVOMIR also uses two additional programs (RNAFOLD and RNASHAPES) for RNA secondary structure prediction. The patterns of read mapping on the reference genome (*B. rapa*) were further examined and false positives were filtered according to recent miRBase guidelines [9].

### TaqMan qPCR for miRNA validation and quantification

The quantitative real-time RT-PCR for miRNA was performed using TaqMan MicroRNA Assays (Applied Biosystems, Foster City, CA, USA). Briefly, 10 ng of small RNA was quantitated from the same small RNA samples used for deep sequencing and reverse transcribed using a specific looped RT primer for each miRNA using a corresponding TaqMan MicroRNA Reverse Transcription kit (Applied Biosystems). The following amplification was performed using a corresponding TaqMan MicroRNA Assay Mix, TaqMan Universal PCR Master Mix and No AmpErase UNG (Applied Biosystems) in a Stratagene Mx3000 instrument with a minimum of 2 replicates. The expression data was normalized to snoR66 expression. The change in miRNA expression was calculated as 2^-(dCt)^ relative to expression in the embryo at 15DAF. The miRNA Taqman Assays for selected miRNAs were listed in Additional file 17: Table S12.

### miRNA Target prediction

The conserved and novel miRNA sequences with reads >300 were used to search against the 90K *B. napus* EST unigenes (http://brassicagenomics.ca/data/brassica_ests.fasta) for potential targets using the program targetFinder (targetFinder 1.6, http://carringtonlab.org/resources/targetfinder) [33].

### Microarray analysis

The *Brassica* 90K Combimatrix Arrays (printed at PBI/NRC in Saskatoon), containing 90K 40nt oligonucleotide probes and predicted to represent 70% of *B. napus* genes (EST contigs), was used for microarray analysis. Total RNAs were extracted using RNA aqueous kit (Life Technologies) and 750 ng of each RNA sample was subjected to one round of amplification using the Amino Allyl MessageAmp aRNA Amplification Kit (Life Technologies), in which the modified nucleotide aa-UTPs were incorporated during an *in vitro* transcription reaction and then coupled to Cy5. About 10 micrograms of Cy5 labeled aRNA were fragmented to the lengths of 50–200 bases. Three micrograms of fragmented aRNAs were added to the hybridization solution. Hybridization was carried out at 45°C overnight using a 25% formamide based solution according to the manufacturer's protocol [76]. Washing and stripping of chips was done according to manufacturer's protocol. Microarrays were scanned on a GenePix scanner 4000B scanner (Axon Instruments) and data extraction from the scanned images was done using GenePix pro 6.1 software. Microarray results were then analyzed using the GeneSpring GX 10 software (Agilent Technologies). Quantile normalization was applied and the median values of probes were used for baseline transformation.

### Data availability

sRNA sequences have been submitted to the Sequence Read Archive (SRA) at NCBI with the accession number SRA055743 (small RNAs in *Brassica napus*). Gene expression (microarray) data has been deposited to the Gene Expression Omnibus (GEO) at NCBI with the accession number GSE43918 (Gene expression profiles during seed development and maturation in *Brassica napus*).

## Competing interests

The authors have no competing interests.

## Authors’ contributions

AJC and DH designed the study. DH and JAF performed the experiments and CK and DH were responsible for data analysis. DH and AJC wrote the manuscript with assistance from JAF and EWT. All authors contributed to and approved the final manuscript.

## Supplementary Material

Additional file 1: Table S1 Conserved miRNAs in Brassica napus with sequences identical to miRNAs in miRBase (Release 18). Click here for file

Additional file 2: Table S2 Additional conserved miRNAs in Brassica napus with sequences homologous to miRNAs in miRBase (Release 18). Click here for file

Additional file 3: Table S3 Expression profiles of conserved miRNAs (threshold read count of 300). Click here for file

Additional file 4: Table S4 MIRNA loci identified in the Brassica napus A genome (Brapa_sequence_v1.1). Click here for file

Additional file 5: Figure S1 Secondary structures of MIRNA precursor loci and patterns of matching sequencing reads in the *Brassica napus* A genome (*Brassica rapa*). For each precursor, small RNA sequencing raw reads (with a minimum number of at least 5 identical reads) from all libraries (10 SOLiD and 17 Illumina, which together total 12 M unique reads) were combined. Annotated miRNA and miRNA* sequences were downloaded from the miRBase database (Release 18). Only reads with perfect matches to the genomic sequence are shown. Green represents the forward (5’ to 3’) reads, red represents reverse reads. The number of reads of each sequence (read count) was integrated into the sequence name as _x [read count for each unique sequence]. The most likely (most abundant) mature miRNA sequence from each MIRNA locus was underlined in red. Click here for file

Additional file 6: Figure S2 Putative novel *Brassica napus* MIRNAs based on the *Brassica* A genome (*Brassica rapa*). The read mapping patterns are displayed and the putative novel miRNA mature sequences underlined in red. Only reads with perfect matches are shown. Green represents the forward reads, red color represents reverse reads. The read count for each specific sequence was integrated into the sequence name as _x [read count of each unique sequence]. The secondary structures of each locus with their folding energy are shown below the genomic sequence. Click here for file

Additional file 7: Table S5 Conserved miRNAs and expression of their target genes. Click here for file

Additional file 8: Figure S5 BiNGO graph of enriched GO terms associated with miRNA targets (based on Arabidopsis homologs). The size of node indicates the number of genes in a GO category. The yellow and orange nodes represent terms with significant enrichment, with darker orange representing a higher degree of significance, as shown by the legend. White nodes are terms with no significant enrichment, but are included because they have a significant child term. Branches of GO with no significant terms are not shown. Click here for file

Additional file 9: Table S6 Putative novel miRNAs and their precursors in the *B. napus* A genome (B. rapa v1.1). Click here for file

Additional file 10: Table S7 Expression of novel putative miRNAs and their targets. Click here for file

Additional file 11: Table S8 Comparison of data reported in this paper to that reported by Zhao et al. [15]. Click here for file

Additional file 12: Figure S3 Integration of putative miRNAs identified from Zhao et al. [15] into mapping results reported in this paper. The mapping patterns of sequence reads to 10 putative miRNA loci are displayed. Three sequences from Zhao et al. were also predicted as novel miRNAs in this study. They are miR2203, miR2204, miR2207, corresponding to our miR5802, miR5806, miR5803. miR2202 is identical to our miR5802*. Because the Zhao et al. designations miR2202-miR2225 were assigned to other species in miRBase (Release 18), we retained our assigned numbers (miR5801-miR5810) for these novel putative miRNAs. Mapping patterns suggest that Zhao et al. miRNAs 2205, 2206 and 2225 are actually siRNAs. Click here for file

Additional file 13: Table S9 Comparison of miRNAs identified in Xu et al. [16] with current study. Click here for file

Additional file 14: Table S10 Expression of known miRNAs in Zhou et al. [15] and current study. Click here for file

Additional file 15: Table S11 Comparison of predicted targets with Category I miRNA target genes in Zhou et al. [15]. Click here for file

Additional file 16: Figure S4 Gene expression profiles during seed development and maturation. Each line represents one gene. A. Expression profiles of 90K Brassica EST contigs (Baseline transformed to median), miR156 targets -- SPLs are highlighted in orange. B. Expression profiles of 90K Brassica EST contigs (no transformation), SPLs are highlighted in orange. C. Expression profiles of 22K Arabidopsis genes (baseline transformed to median) during seed development (Schmid et al., 2005), SPLs are highlighted in orange. Click here for file

Additional file 17: Table S12 miRNA Taqman Assays for qPCR validation. Click here for file
