# Supplementary material for: MicroRNAs and their putative targets in Brassica napus seed maturation
Source: BMC Genomics. 2013 Feb 28;14:140. doi: 10.1186/1471-2164-14-140 (PMC3602245; doi:10.1186/1471-2164-14-140)
Supplement: Additional file 5: Figure S1 — Secondary structures of MIRNA precursor loci and patterns of matching sequencing reads in the Brassica napus A genome (Brassica rapa). For each precursor, small RNA sequencing raw reads (with a minimum number of at least 5 identical reads) from all libraries (10 SOLiD and 17 Illumina, which together total 12 M unique reads) were combined. Annotated miRNA and miRNA* sequences were downloaded from the miRBase database (Release 18). Only reads with perfect matches to the genomic sequence are shown. Green represents the forward (5’ to 3’) reads, red represents reverse reads. The number of reads of each sequence (read count) was integrated into the sequence name as _x [read count for each unique sequence]. The most likely (most abundant) mature miRNA sequence from each MIRNA locus was underlined in red. [file 1471-2164-14-140-S5.docx]

Figure S1. Secondary structures of MIRNA precursor loci and patterns of matching sequencing reads in the *Brassica napus* A genome (*Brassica rapa*). For each precursor, small RNA sequencing raw reads (with a minimum number of at least 5 identical reads) from all libraries (10 SOLiD and 17 Illumina, which together total 12M unique reads) were combined. Annotated miRNA and miRNA* sequences were downloaded from the miRBase database (Release 18). Only reads with perfect matches to the genomic sequence are shown. Green represents the forward (5’ to 3’) reads, red represents reverse reads. The number of reads of each sequence (read count) was integrated into the sequence name as _x [read count for each unique sequence]. The most likely (most abundant) mature miRNA sequence from each MIRNA locus was underlined in red.

**MIR156**


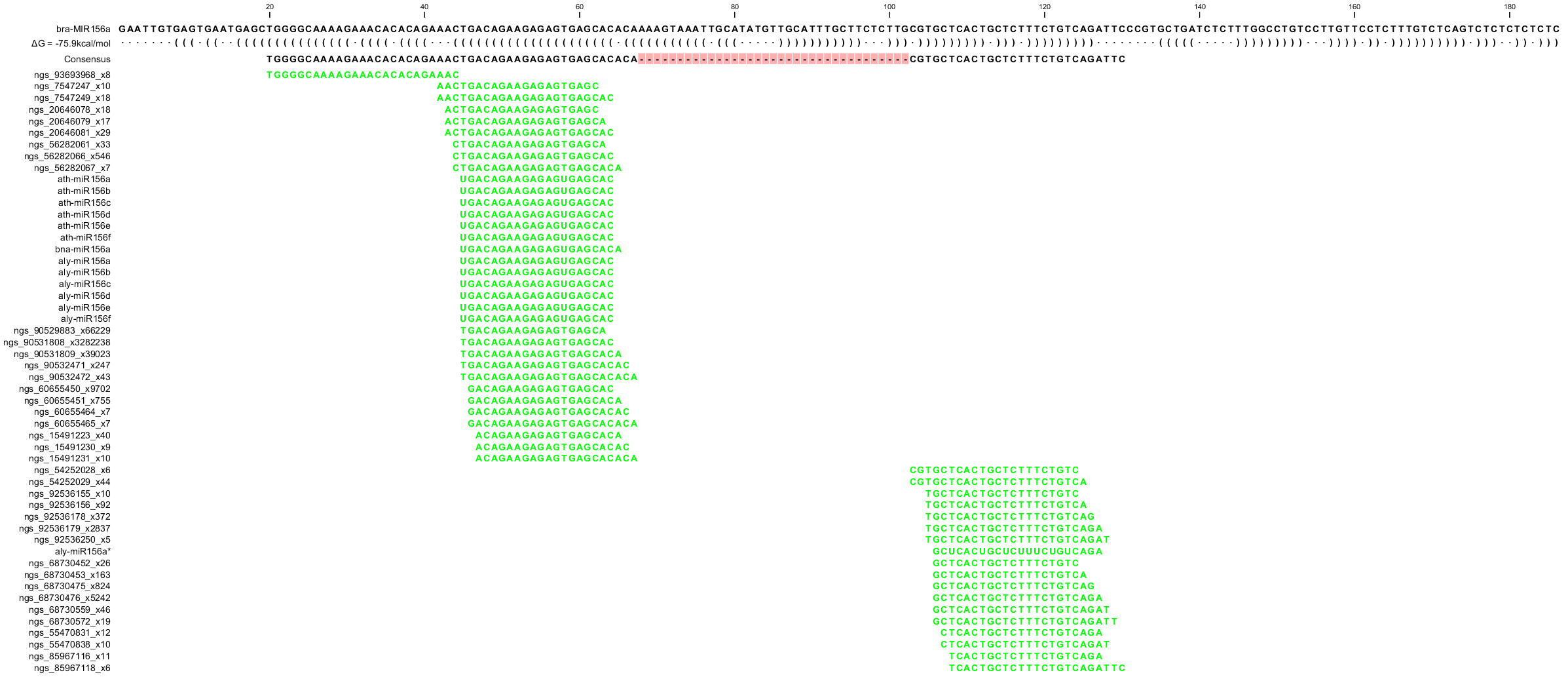


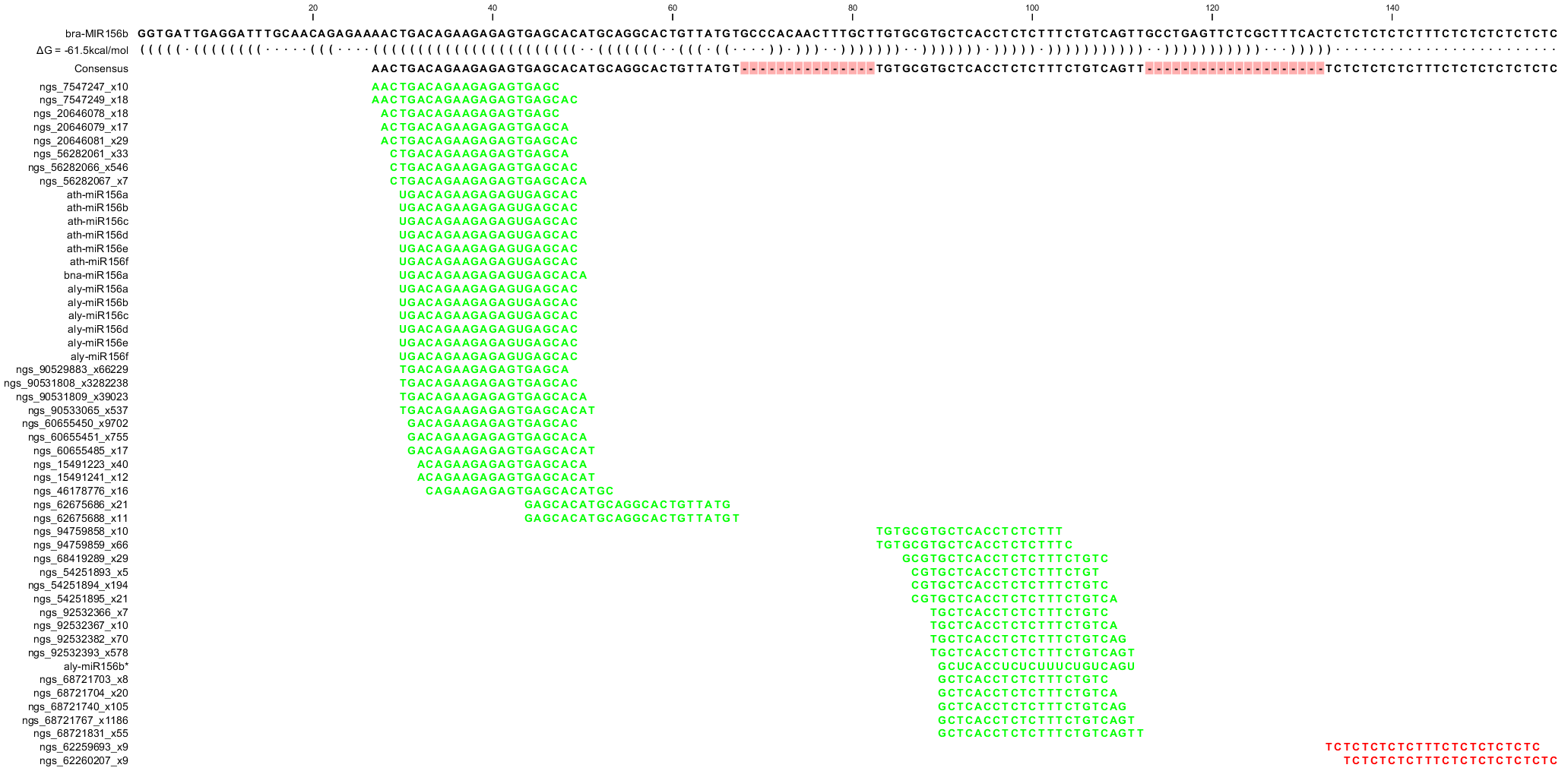

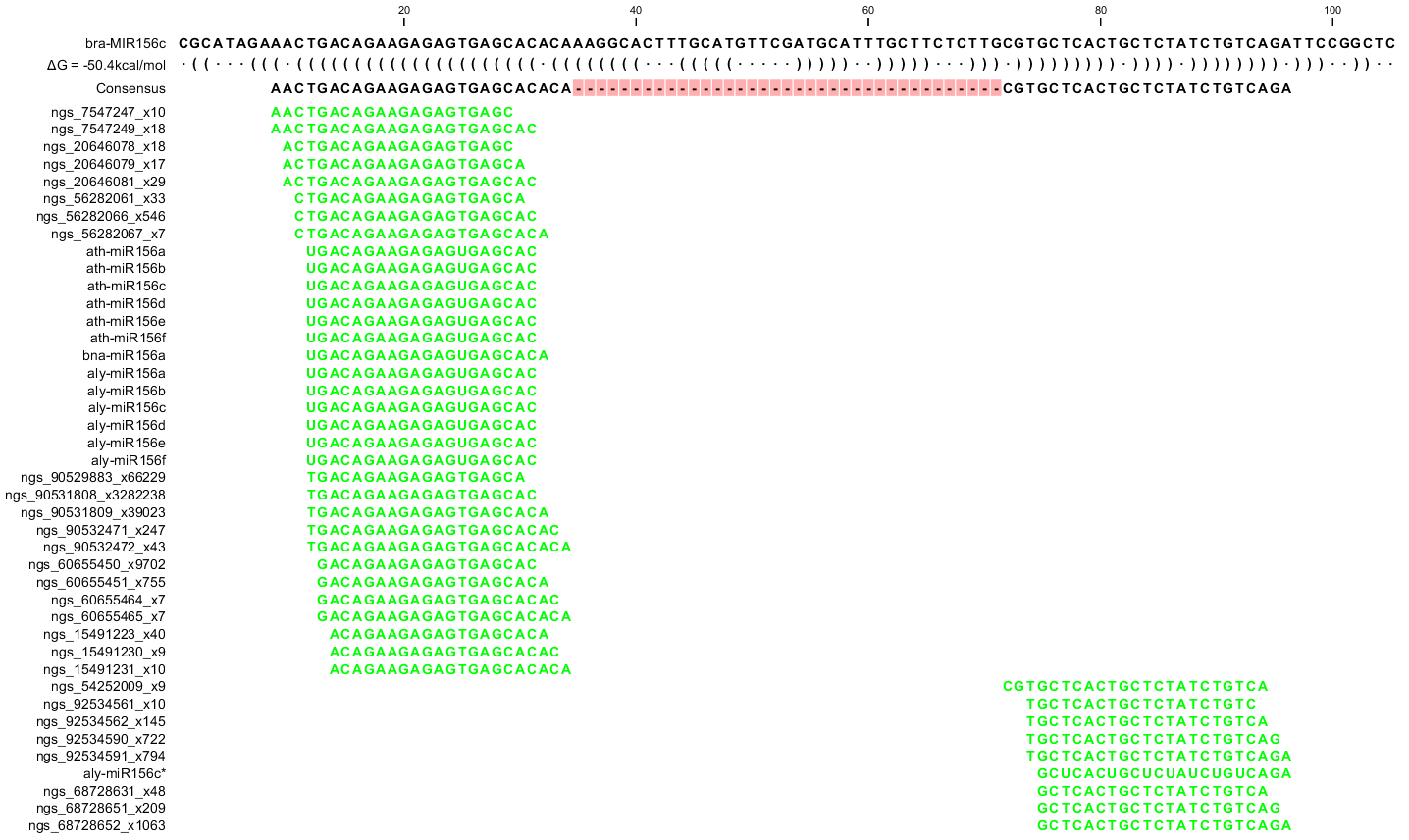

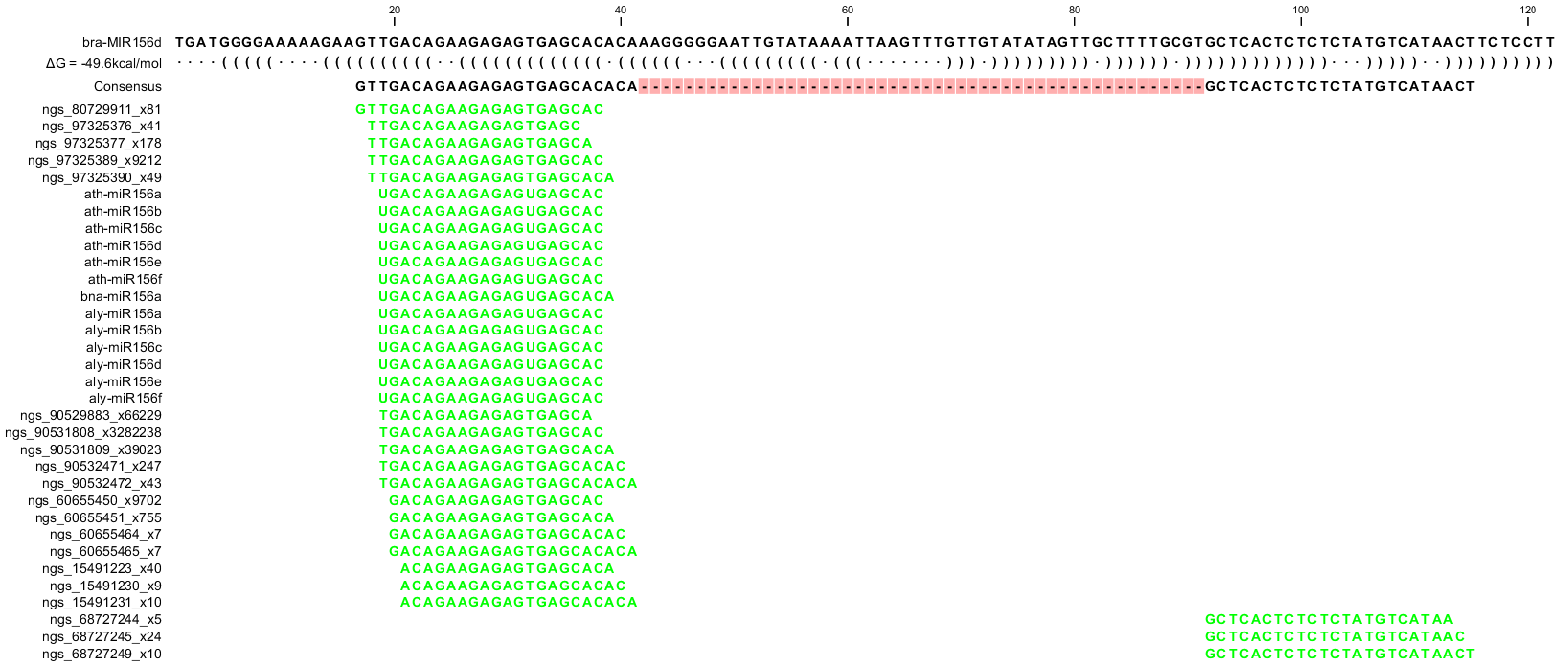

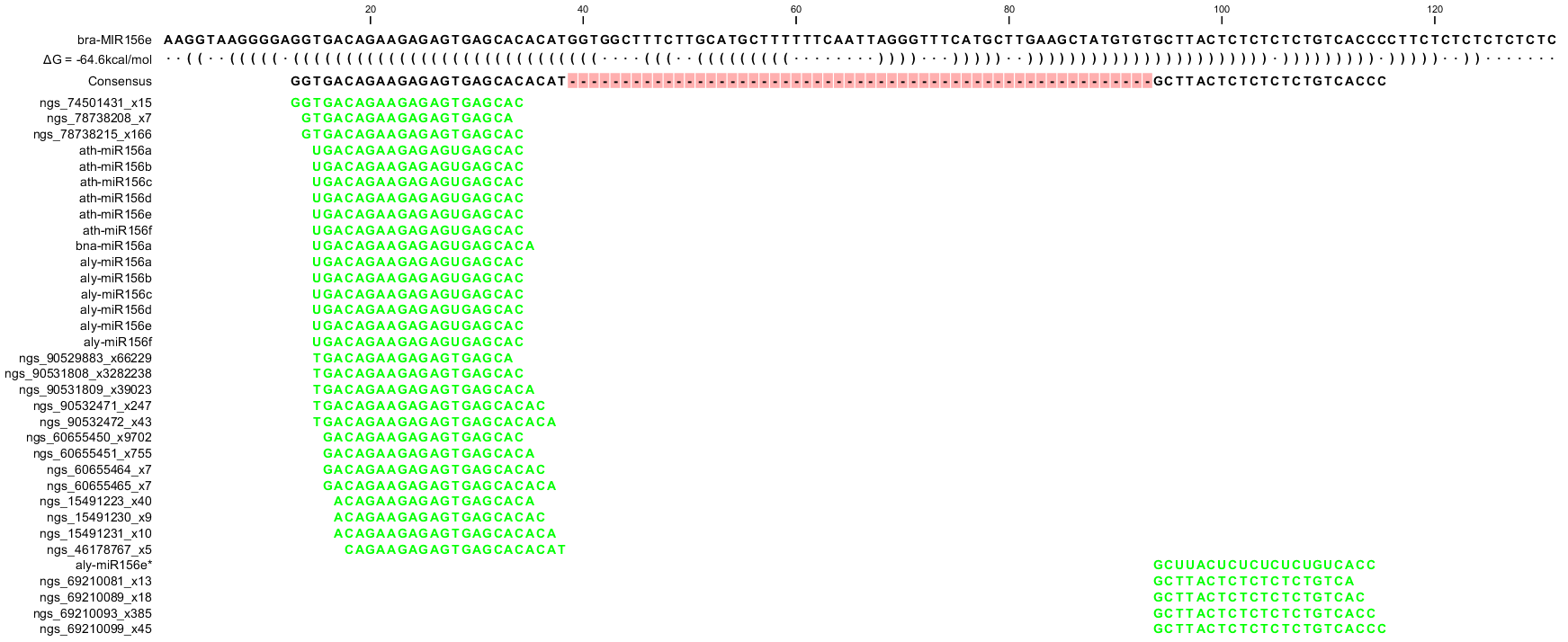

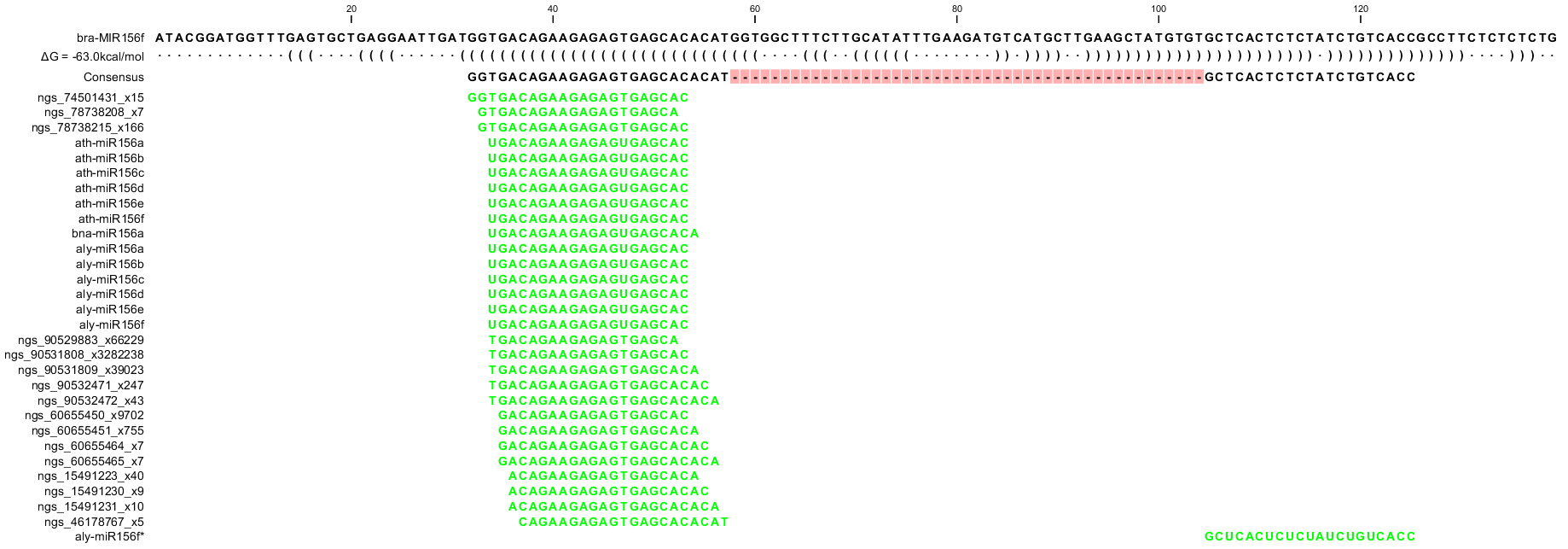


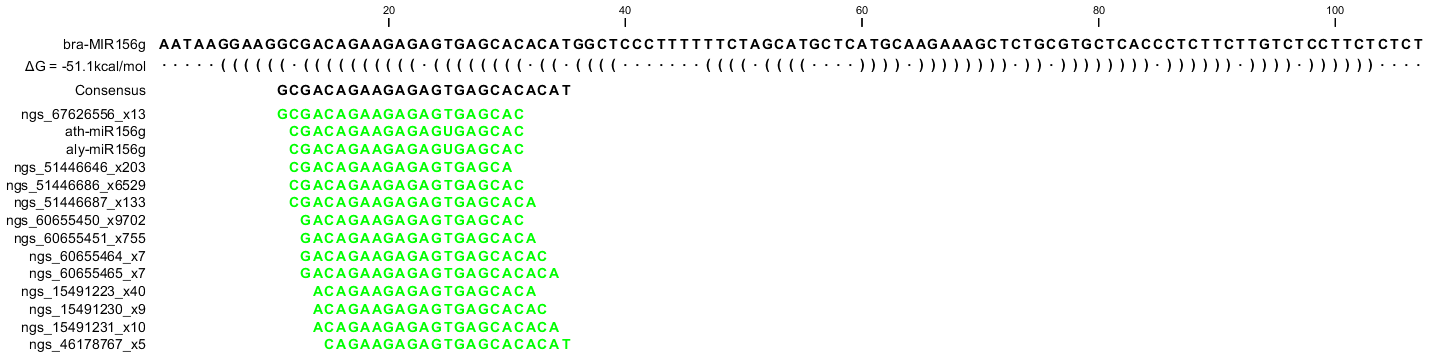


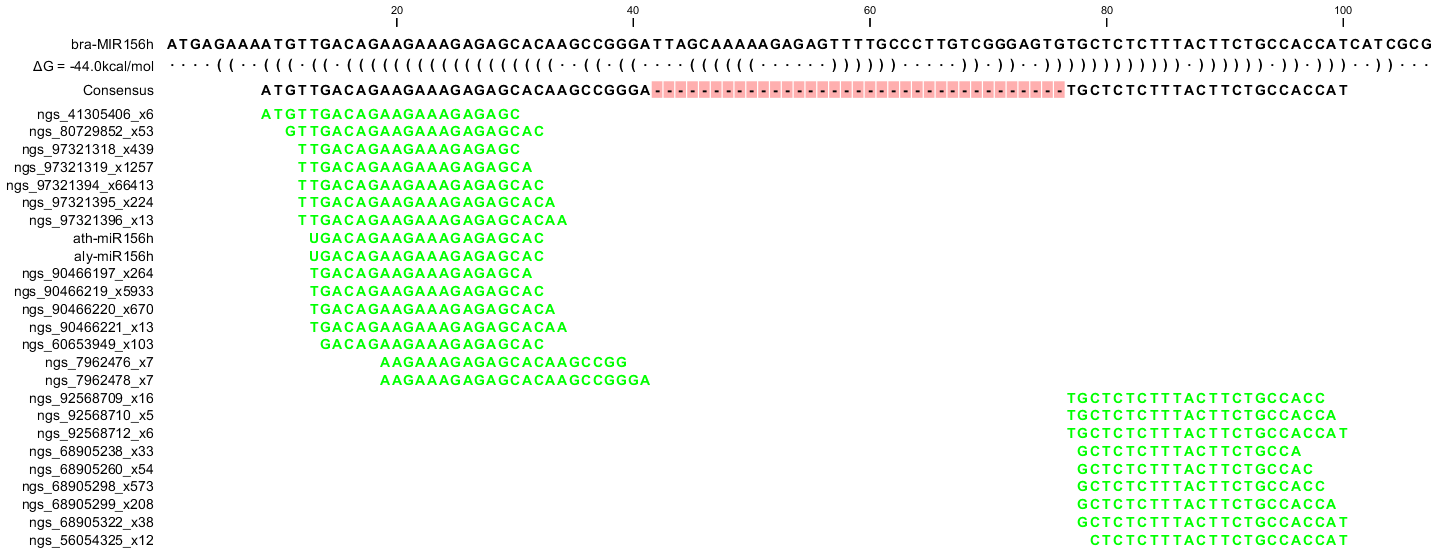

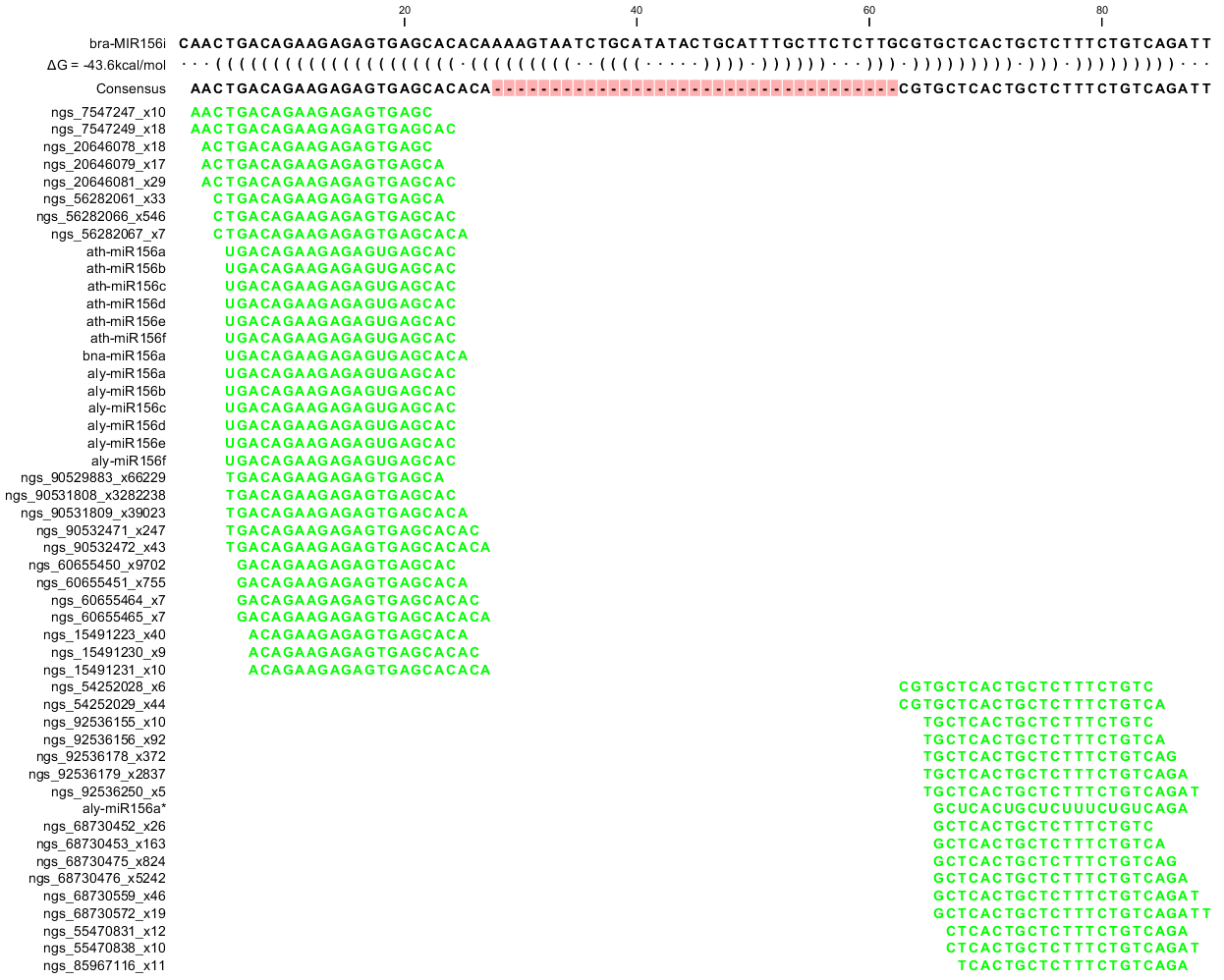

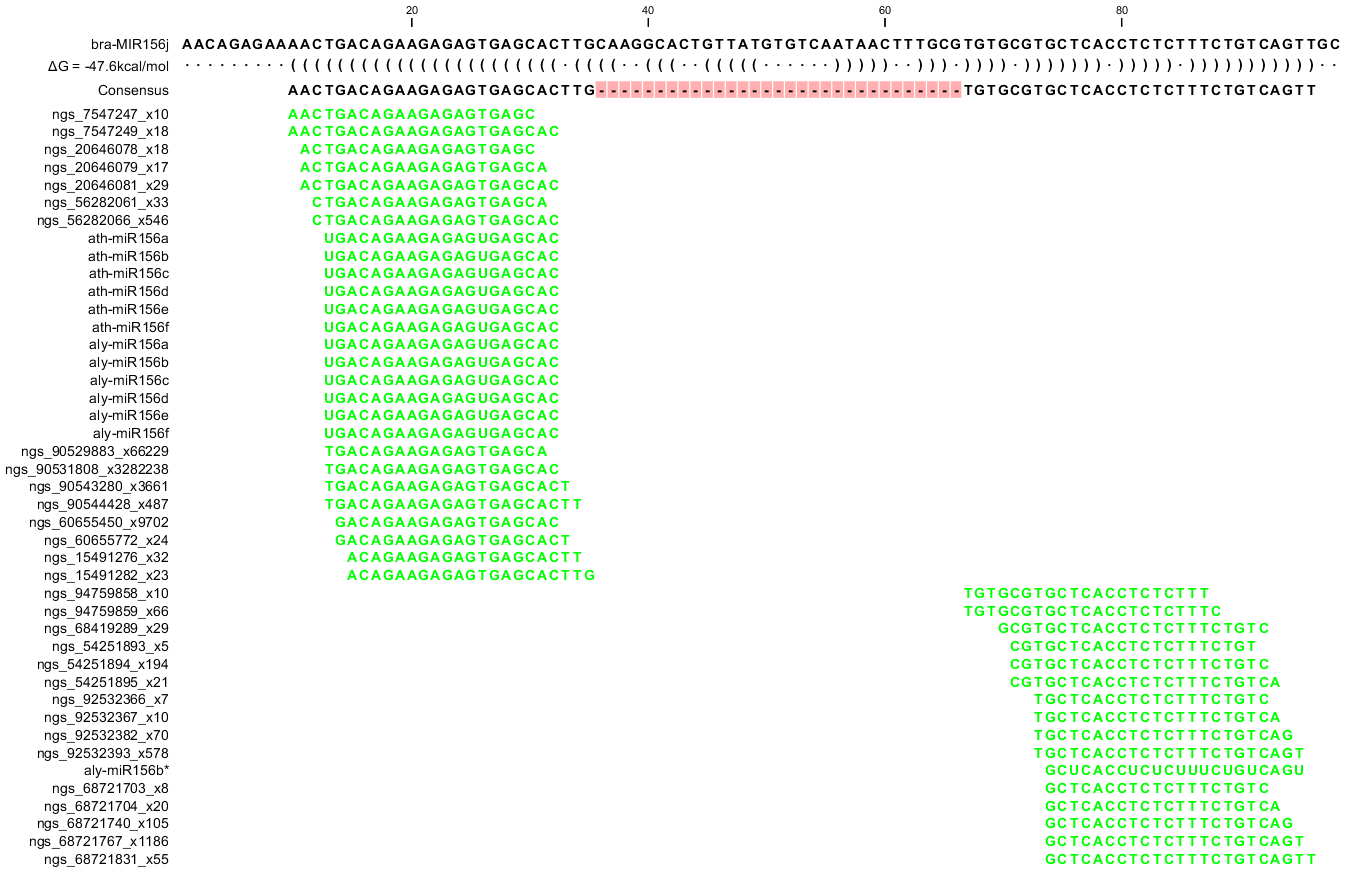

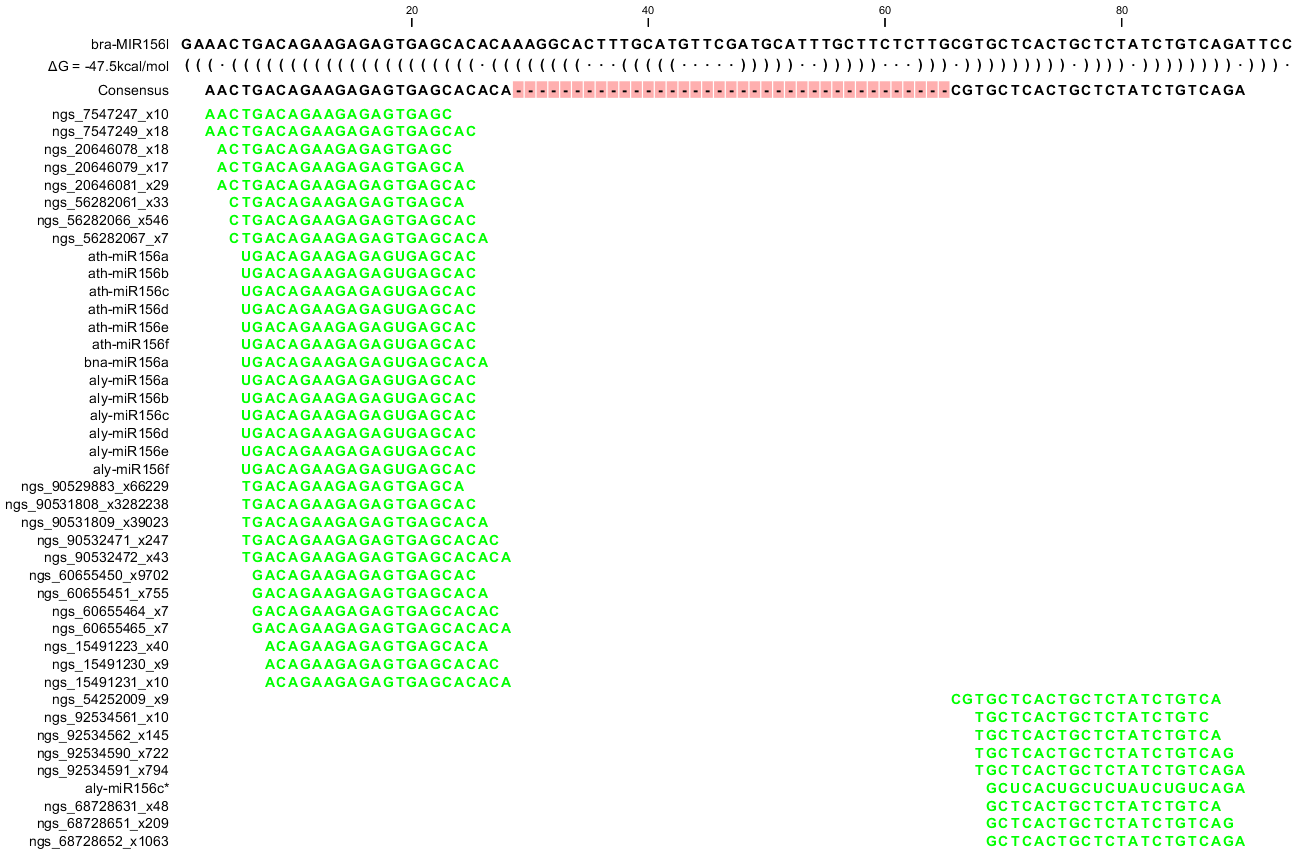

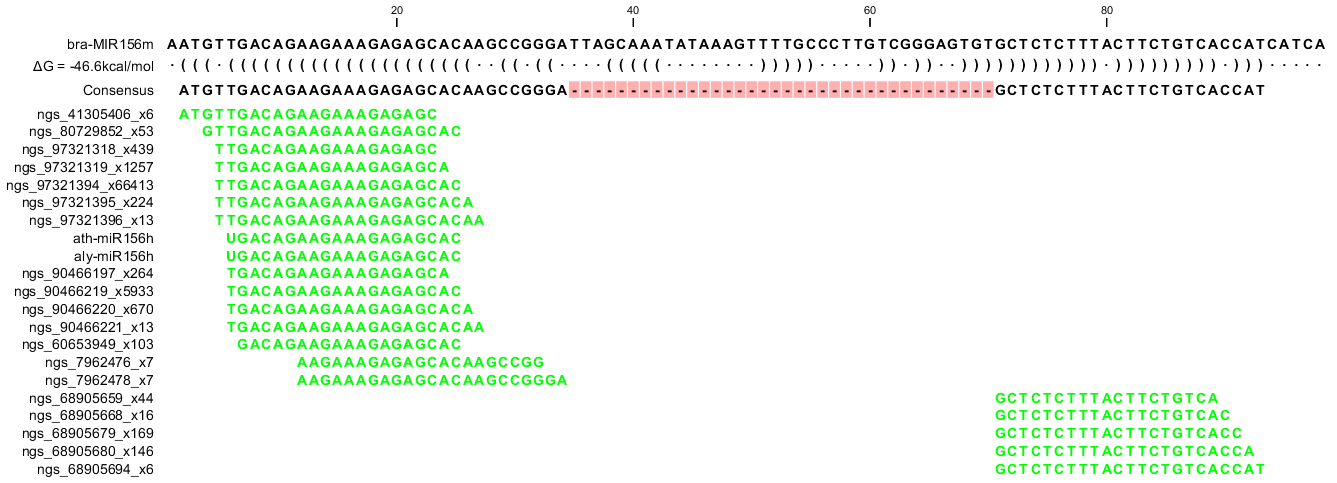

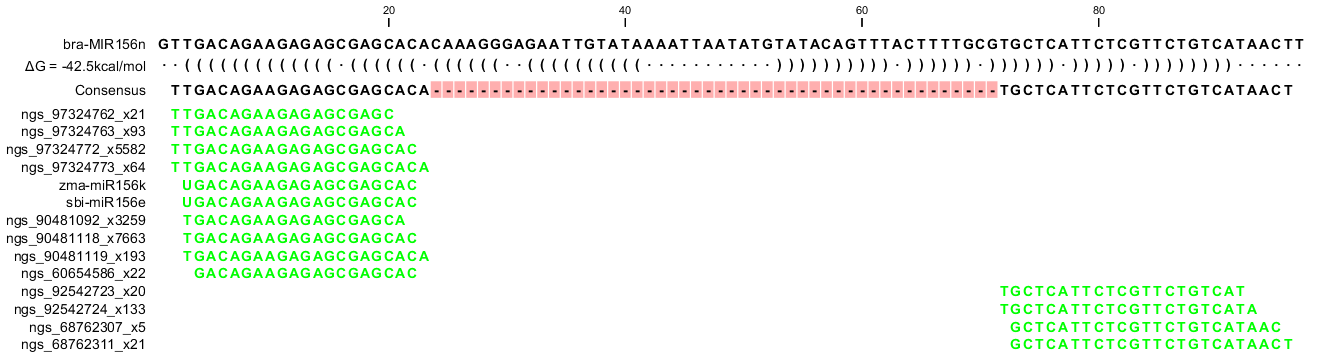

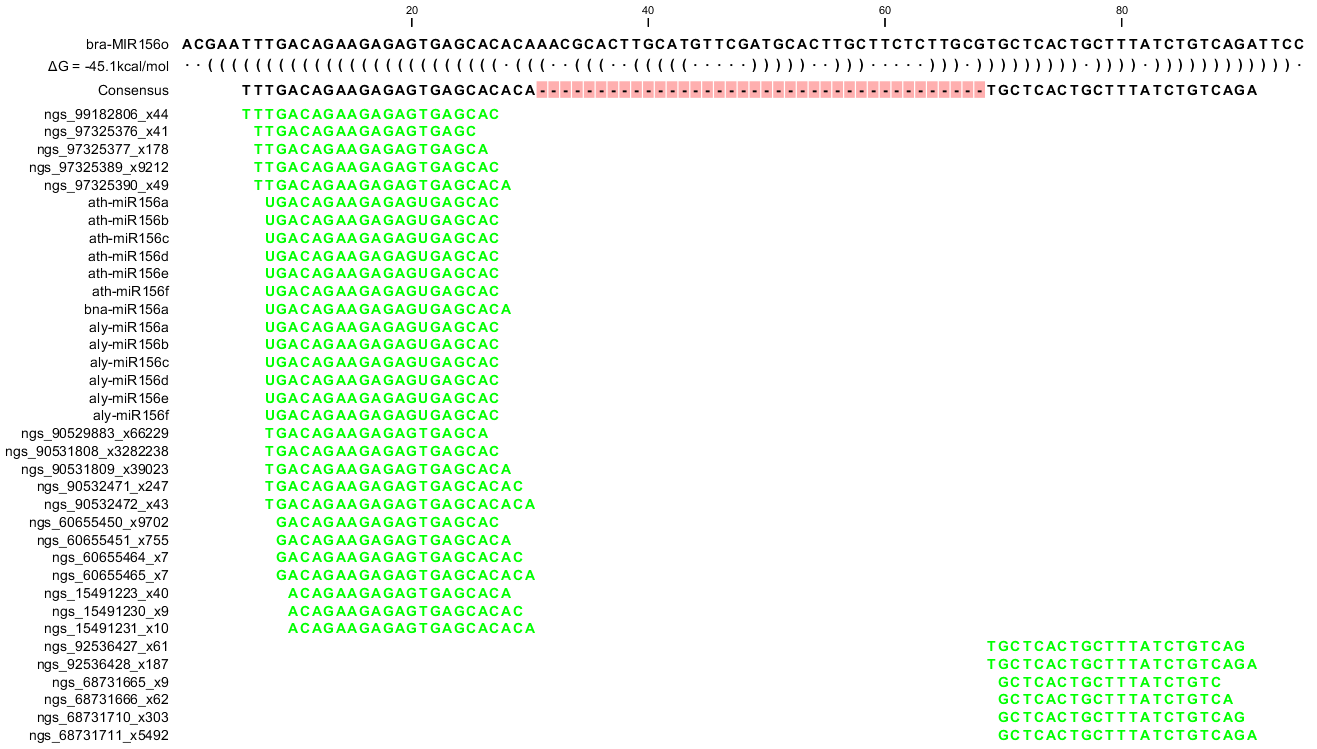

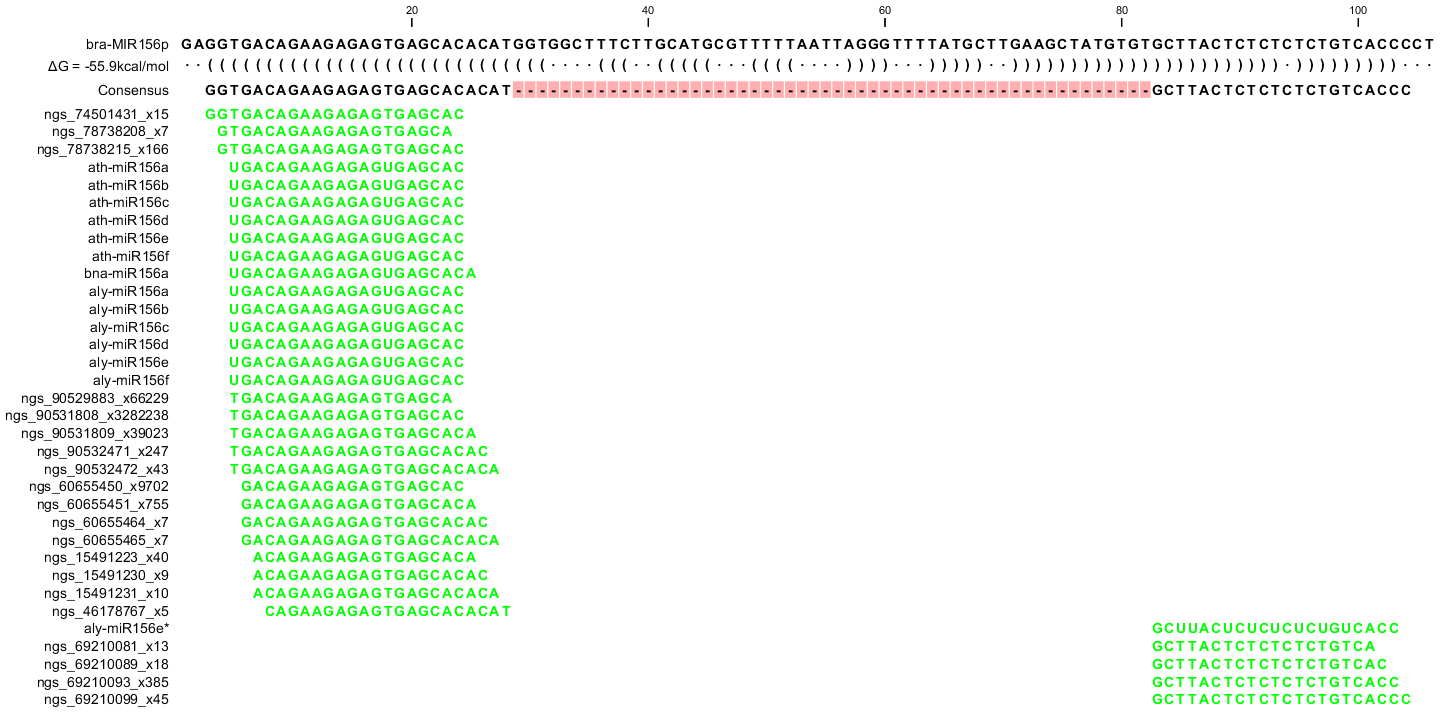

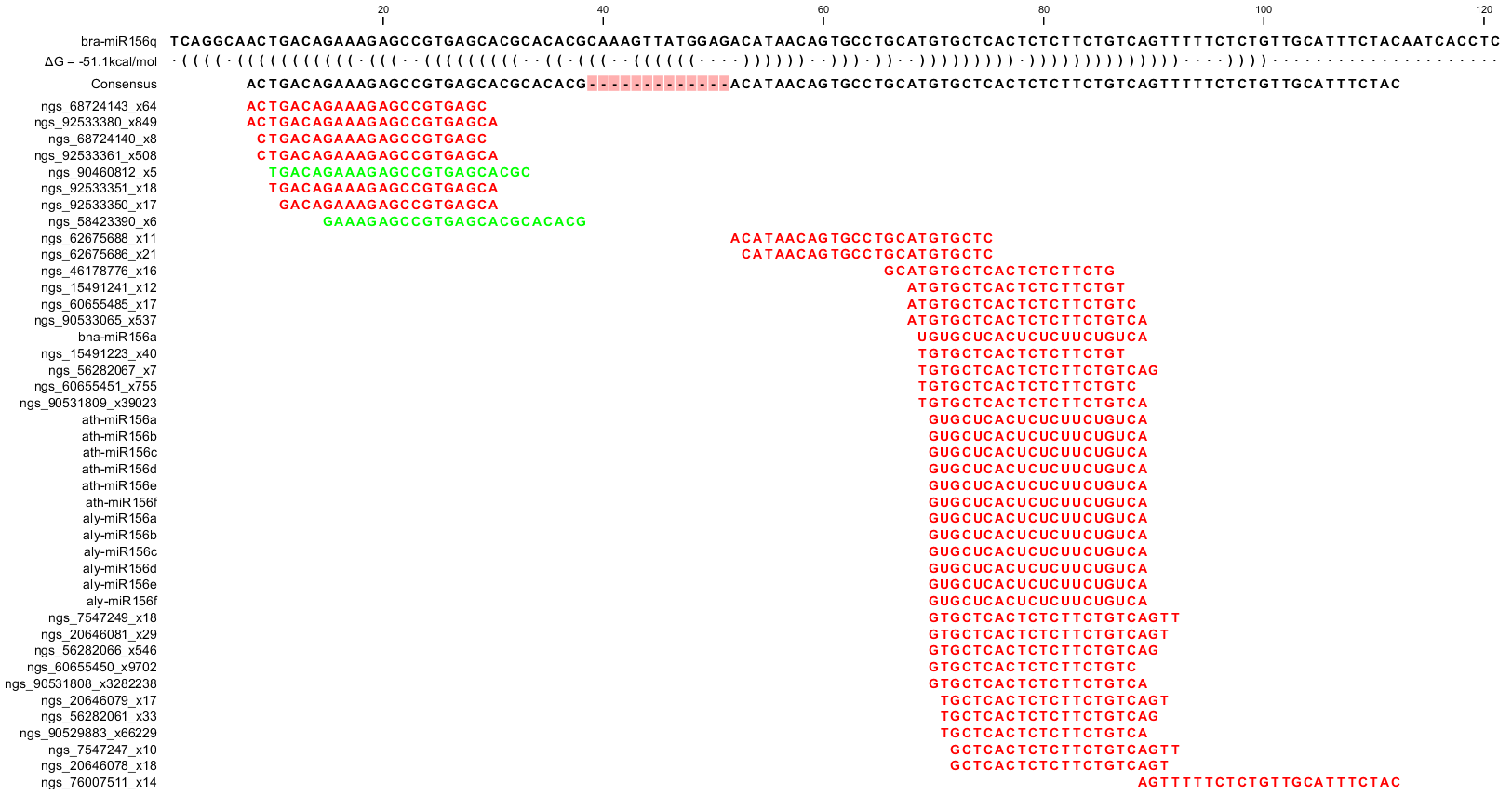


**
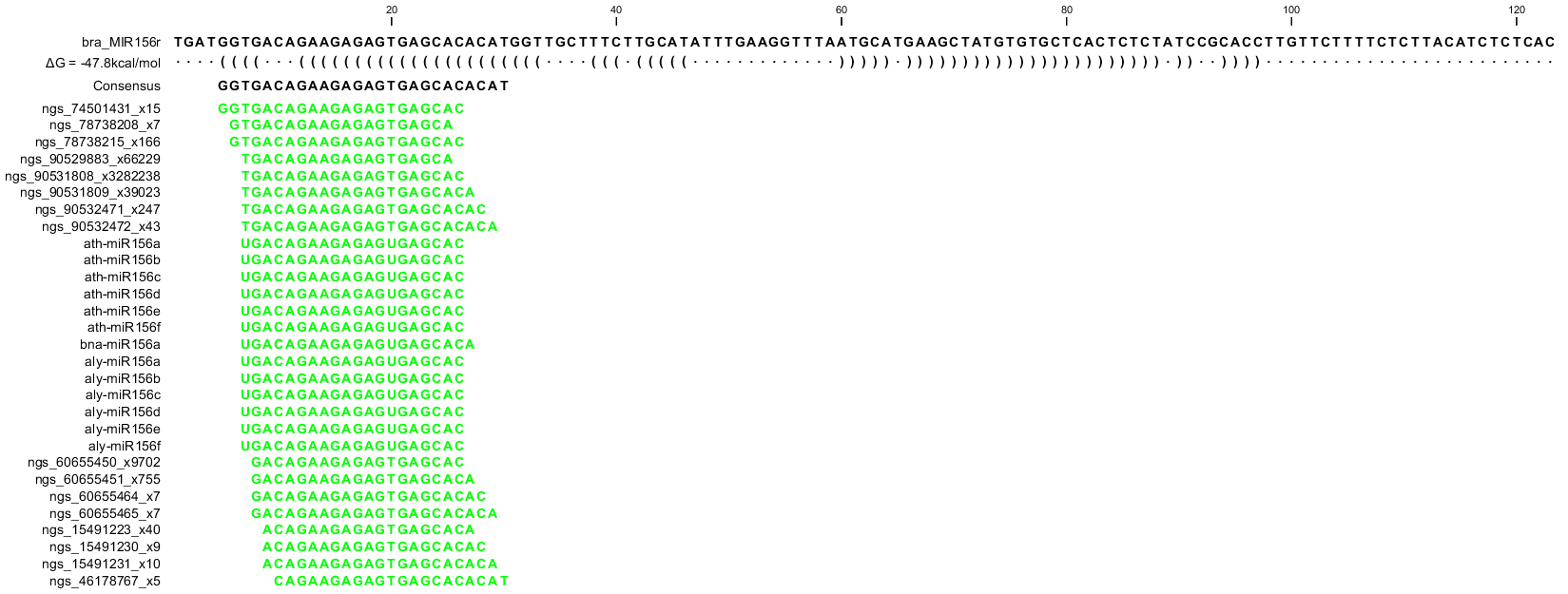
**

**MIR157**
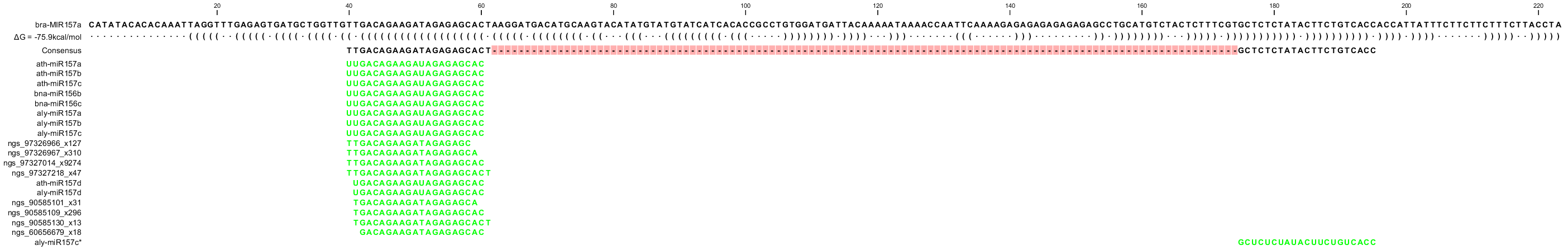

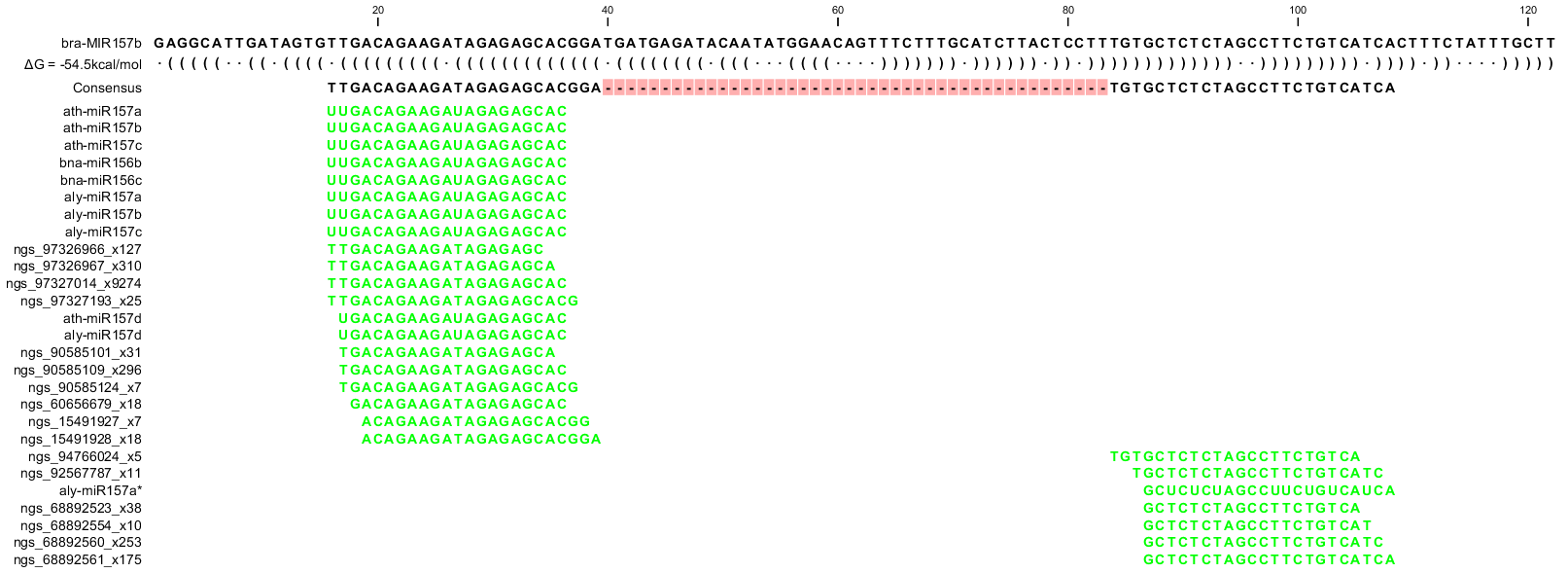

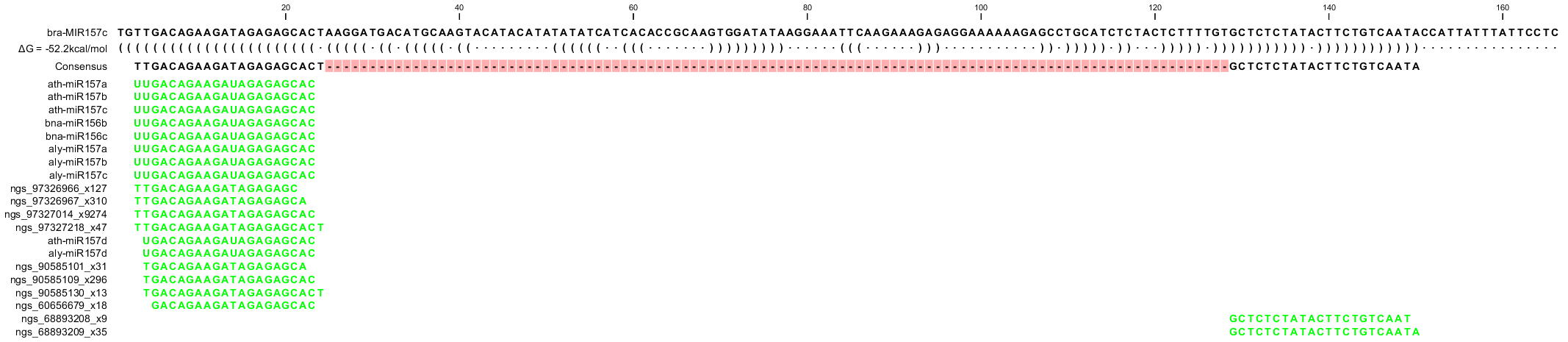


**MIR158**


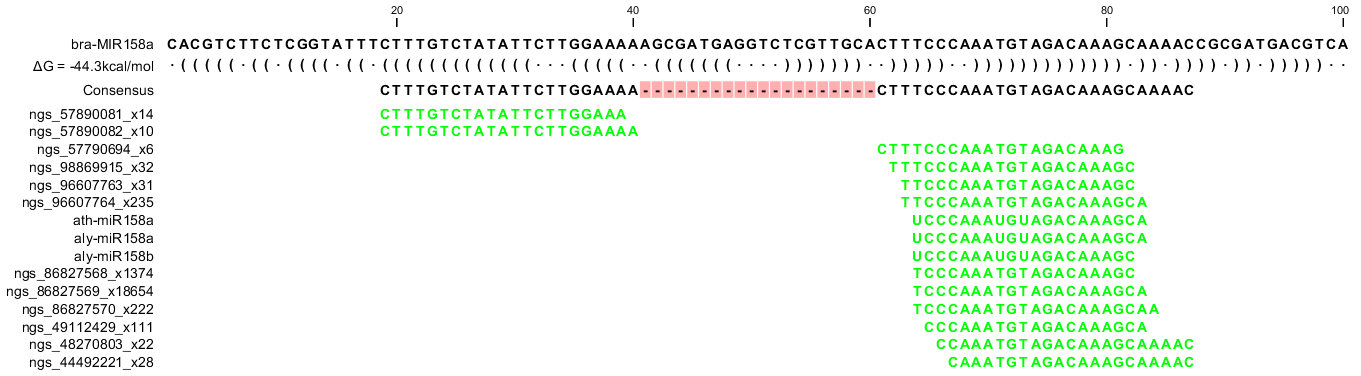


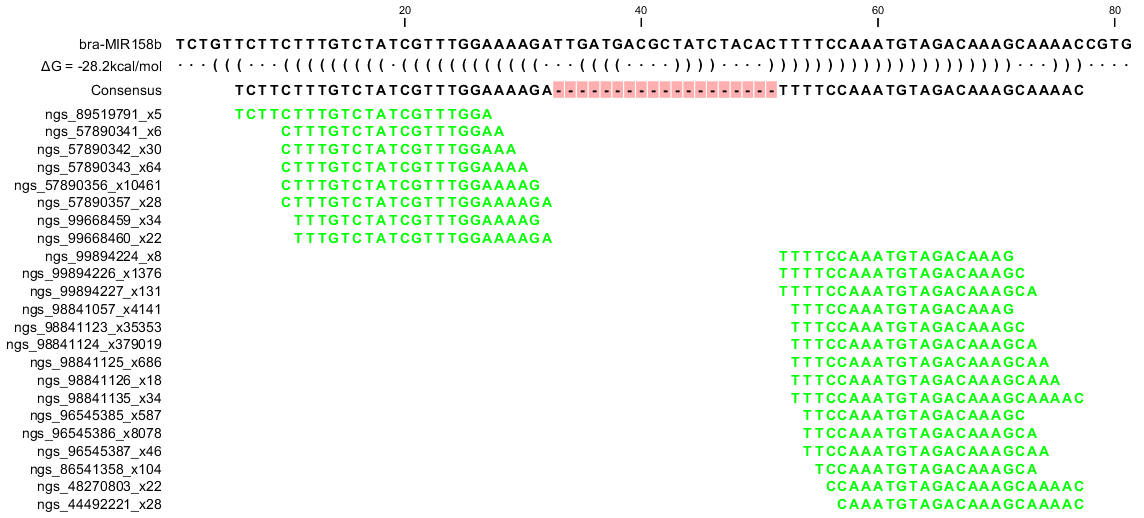


**MIR159**


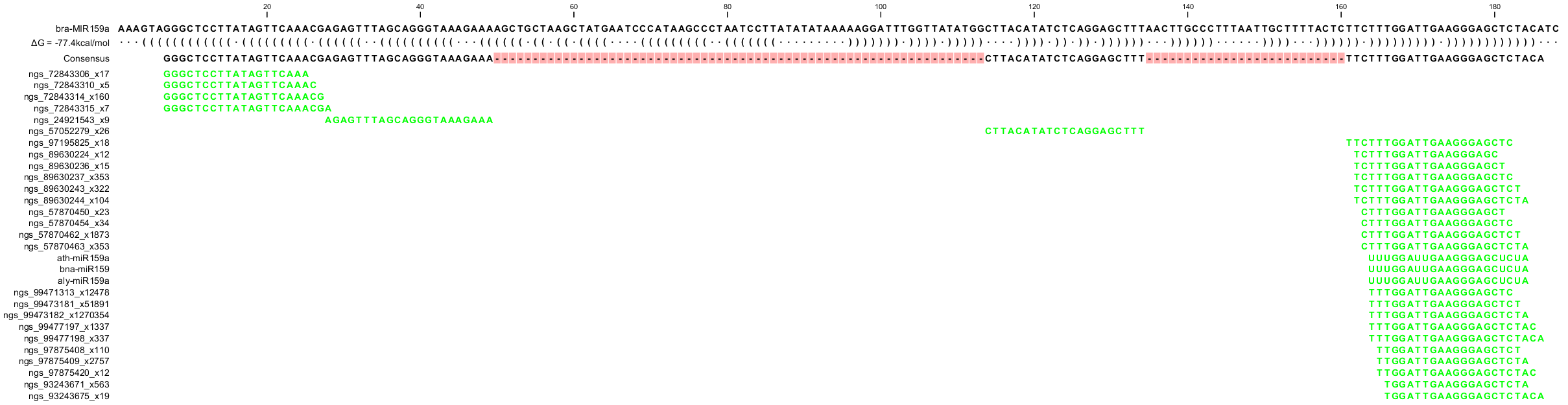


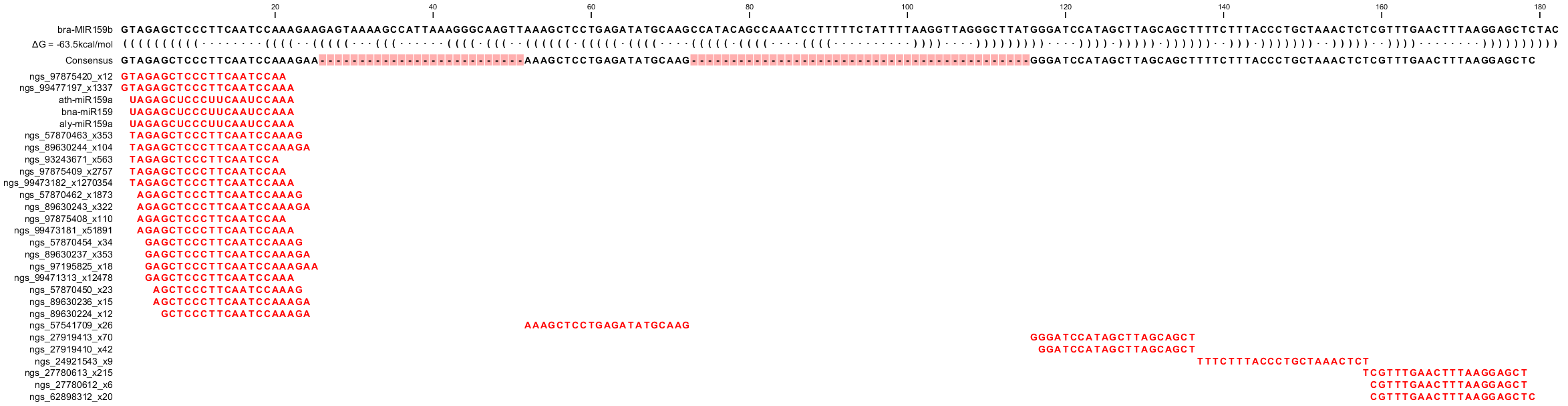


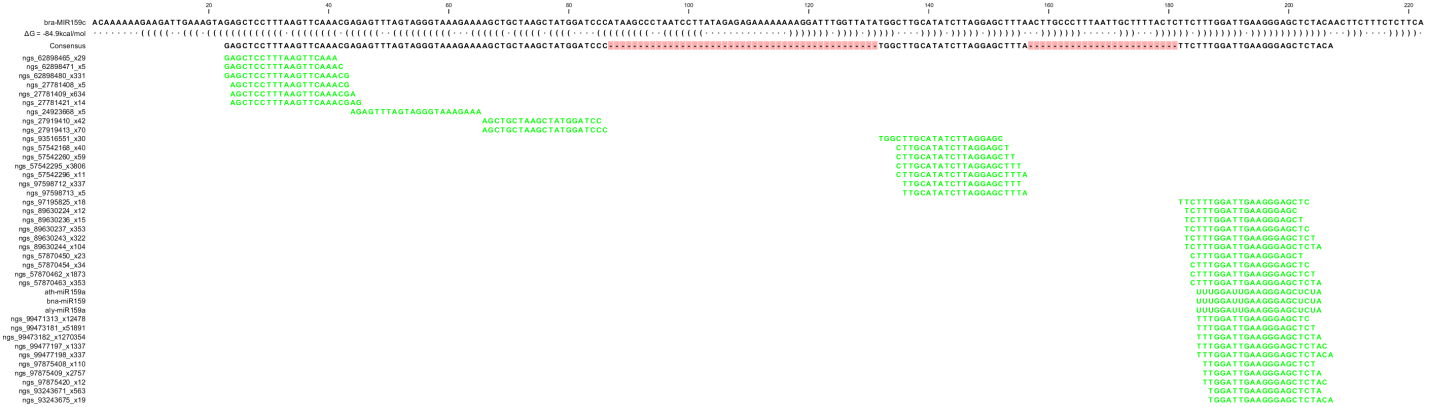

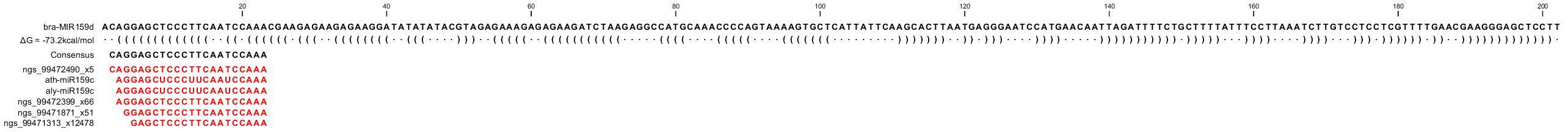


**MIR160**


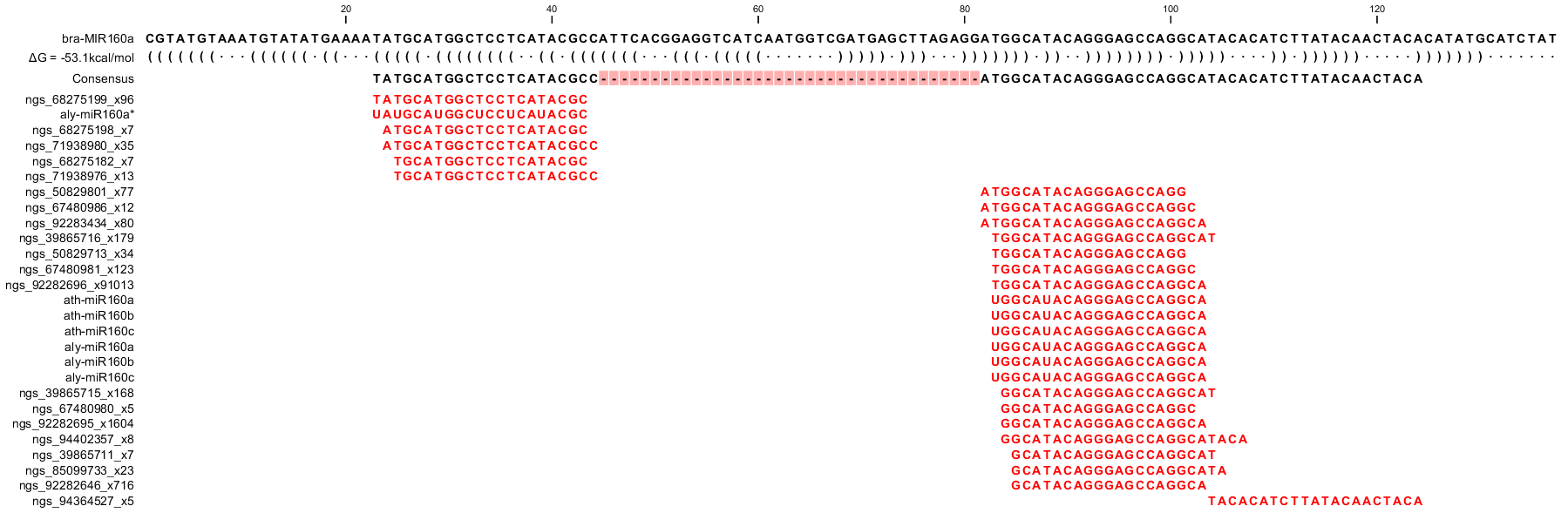

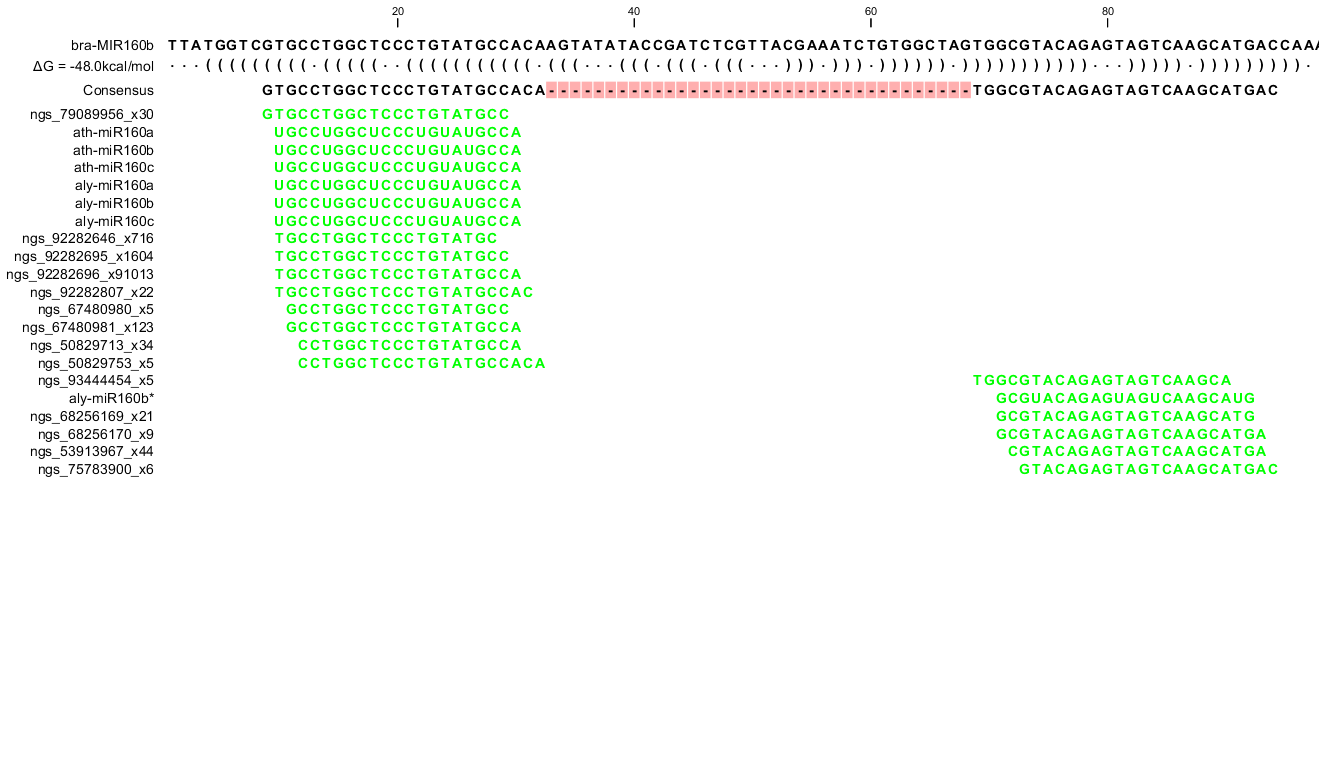

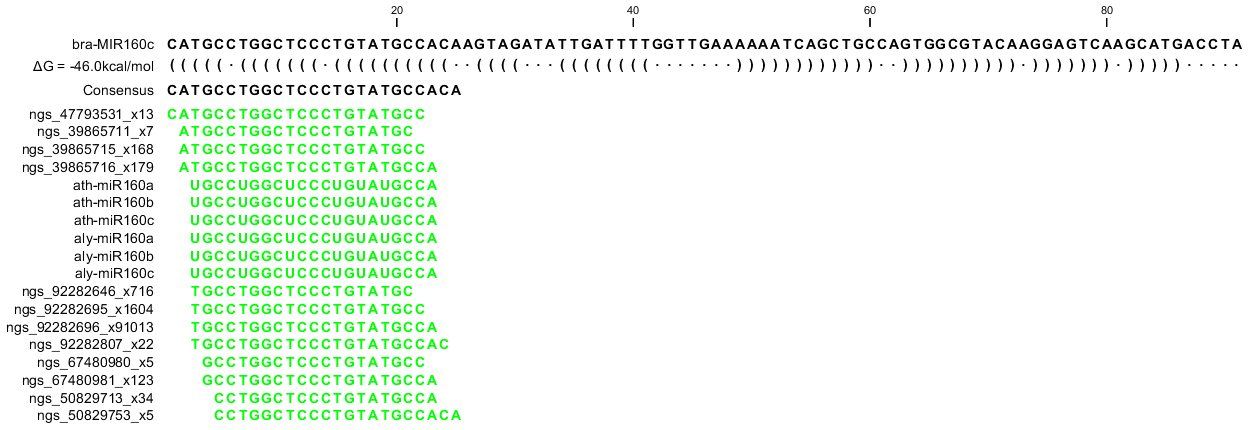

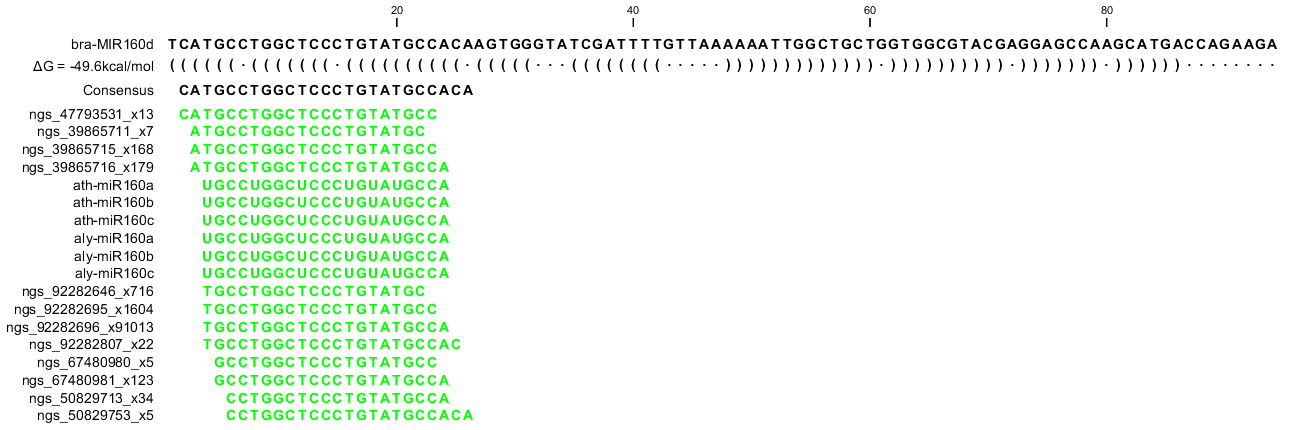

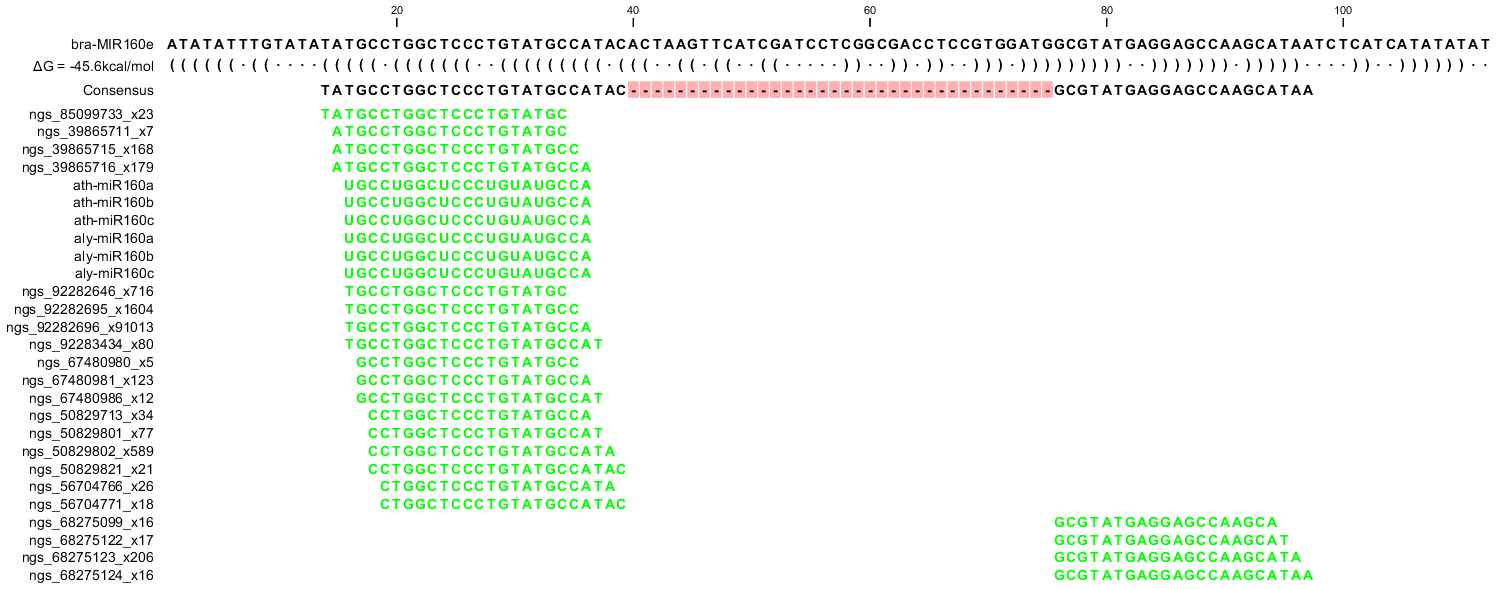

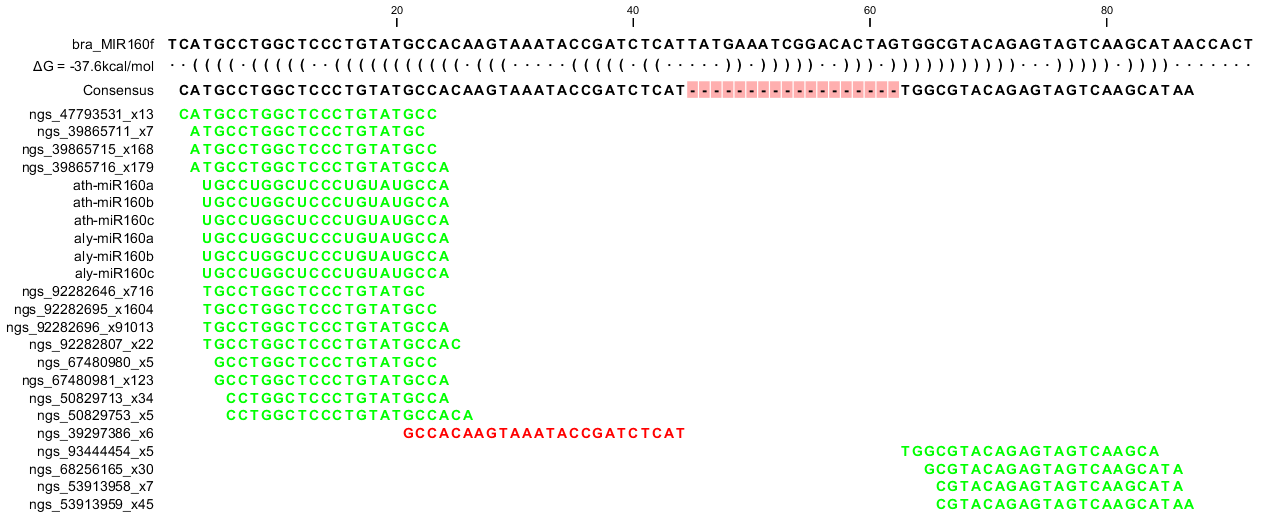


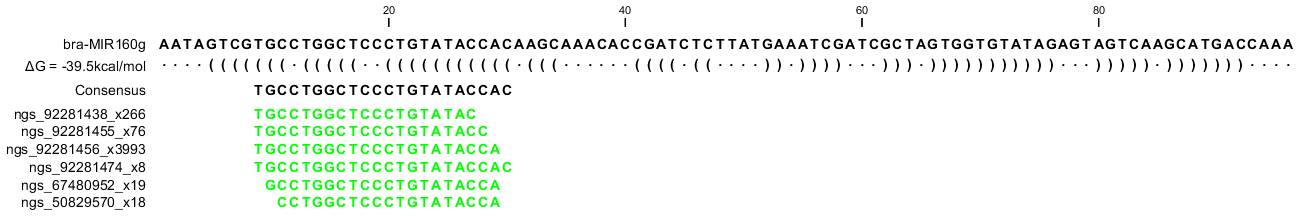


**MIR161**


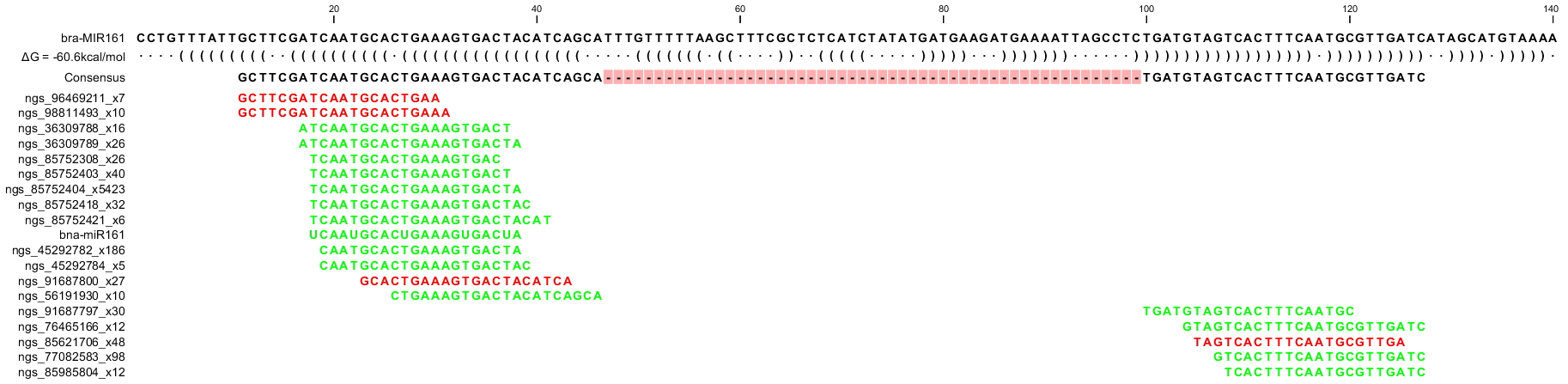


**MIR162**


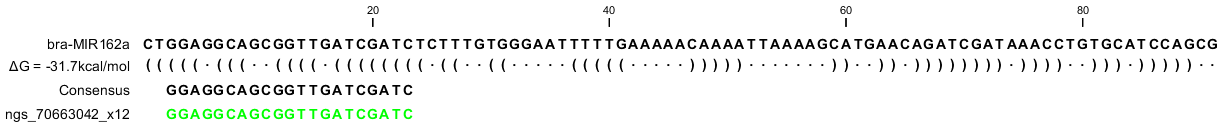


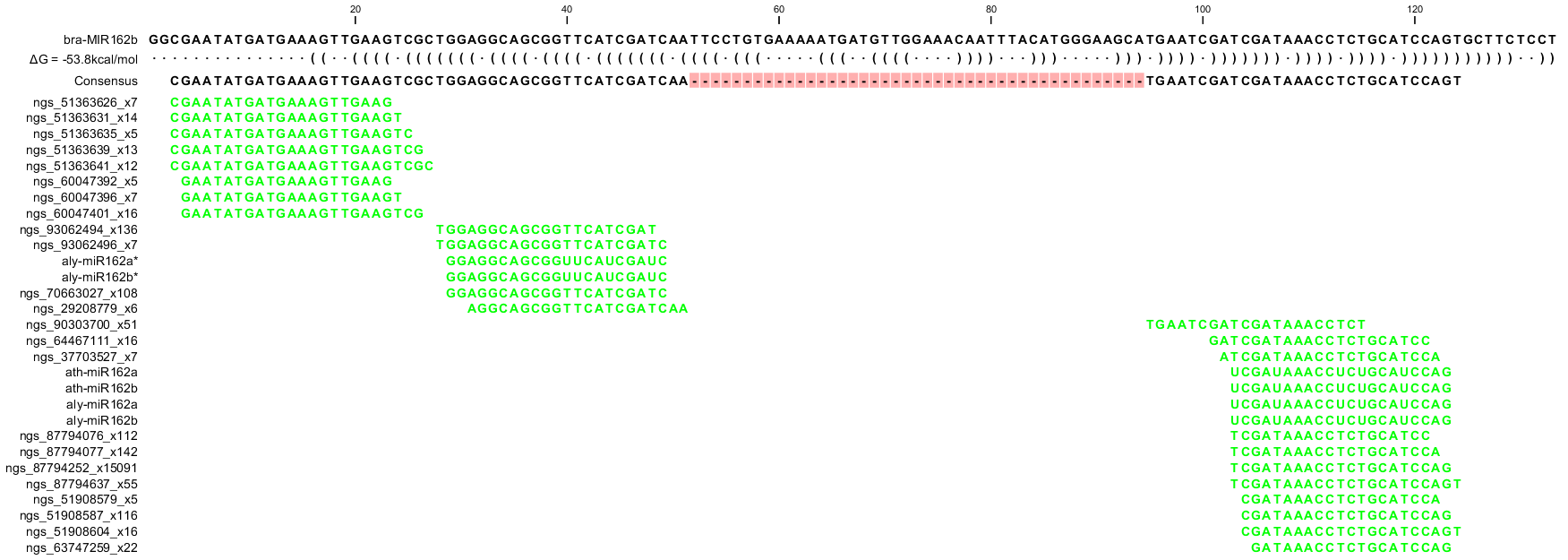

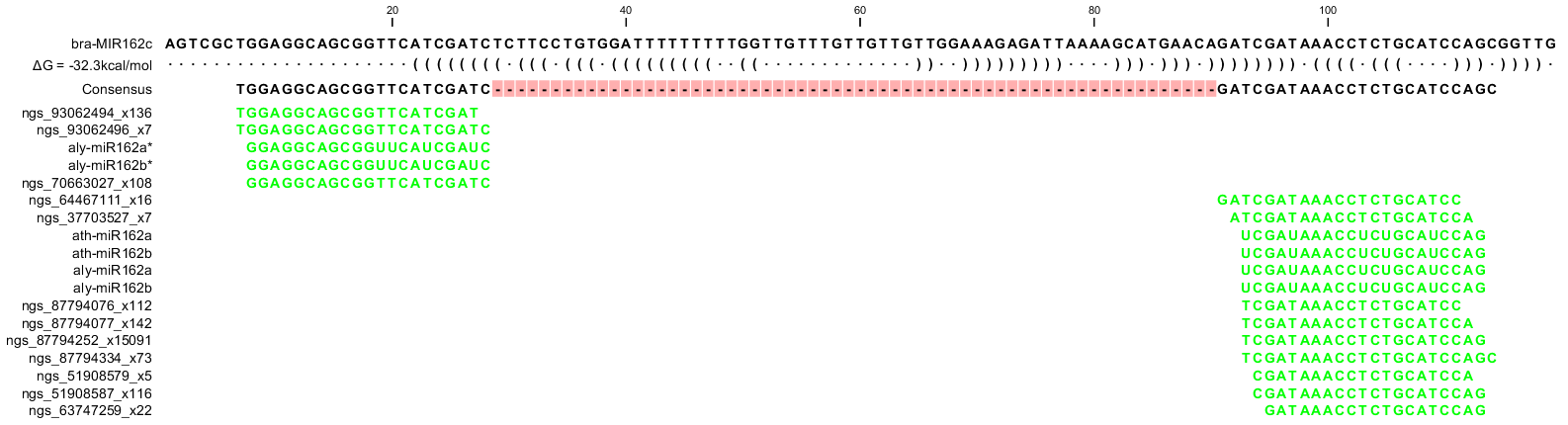


**MIR164**


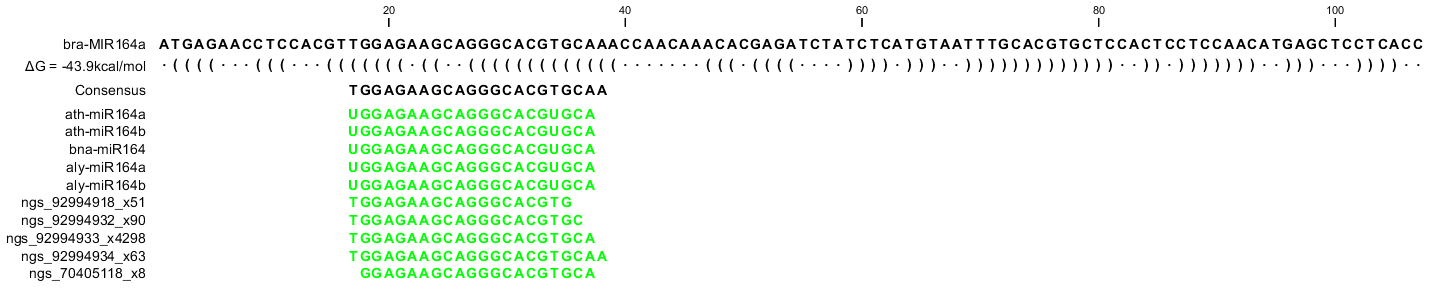

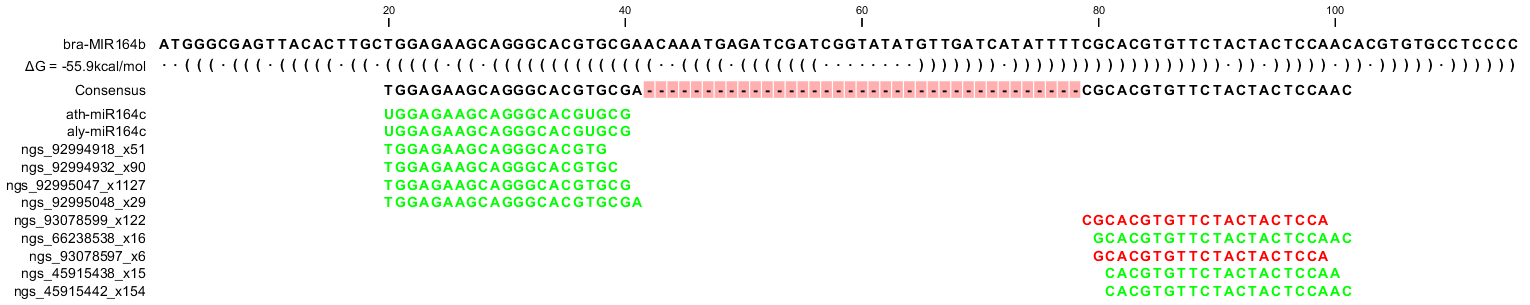

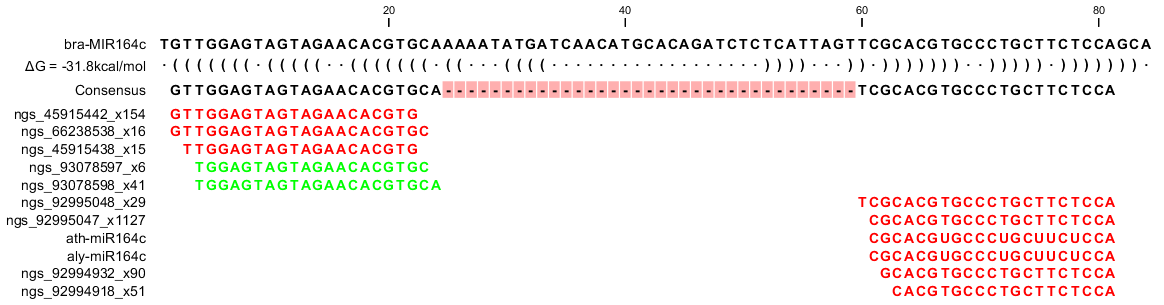

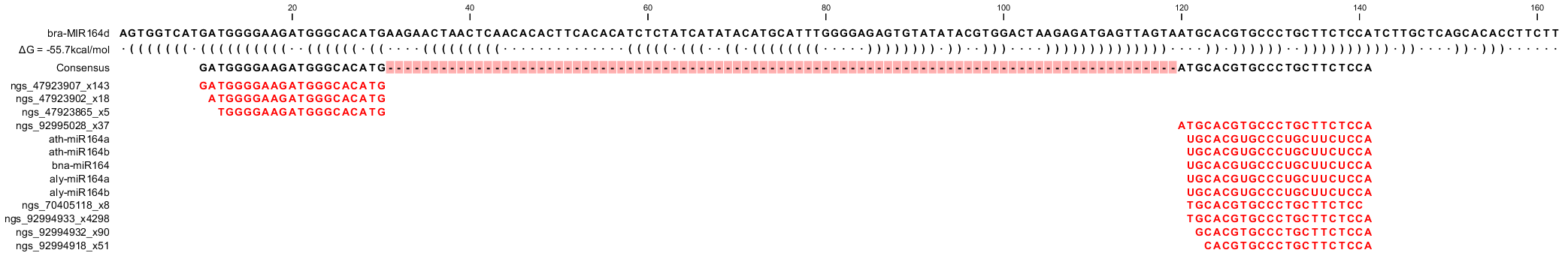


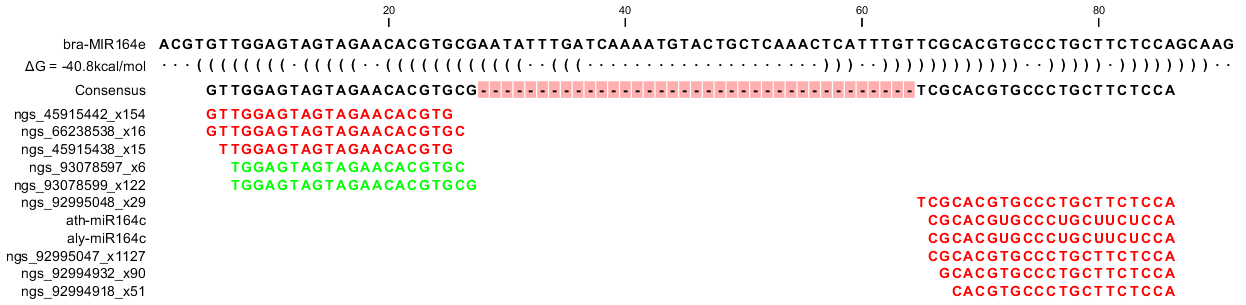


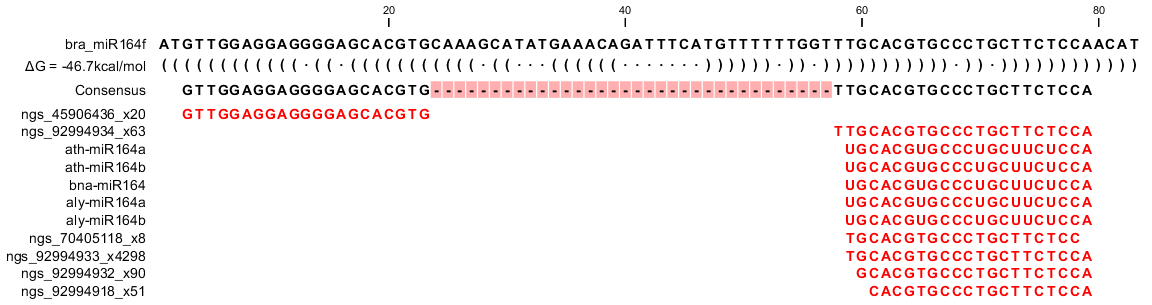


**MIR165**


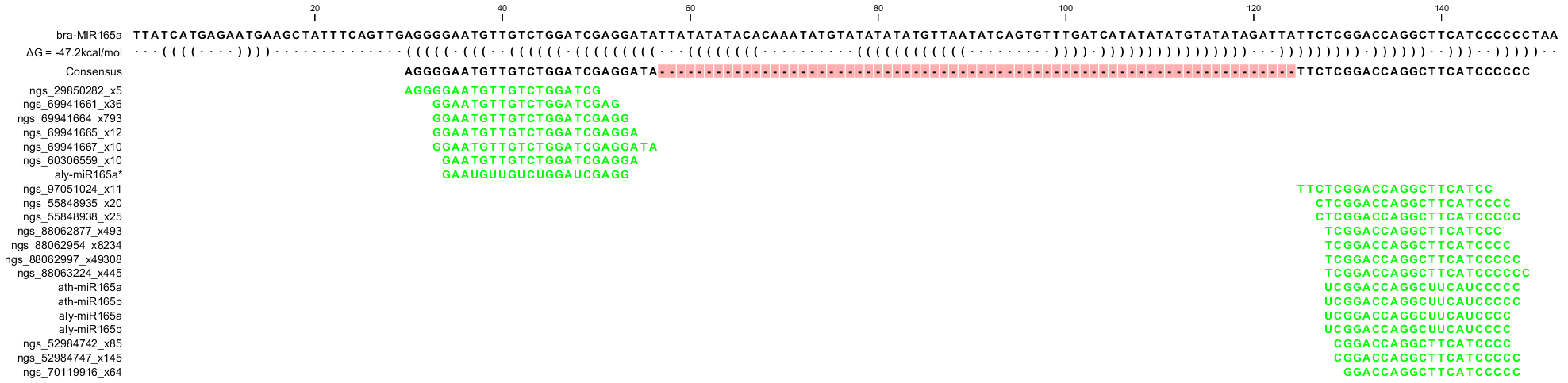

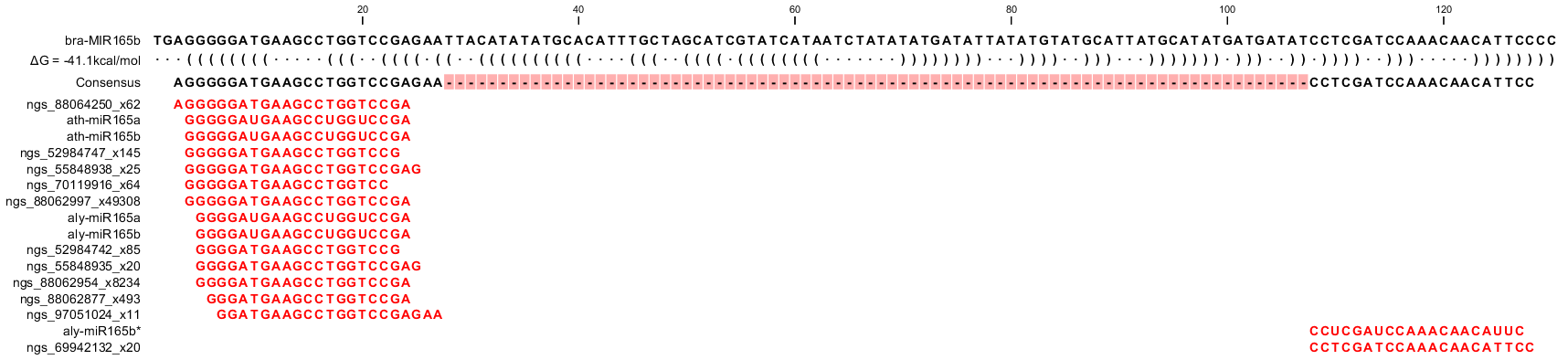


**
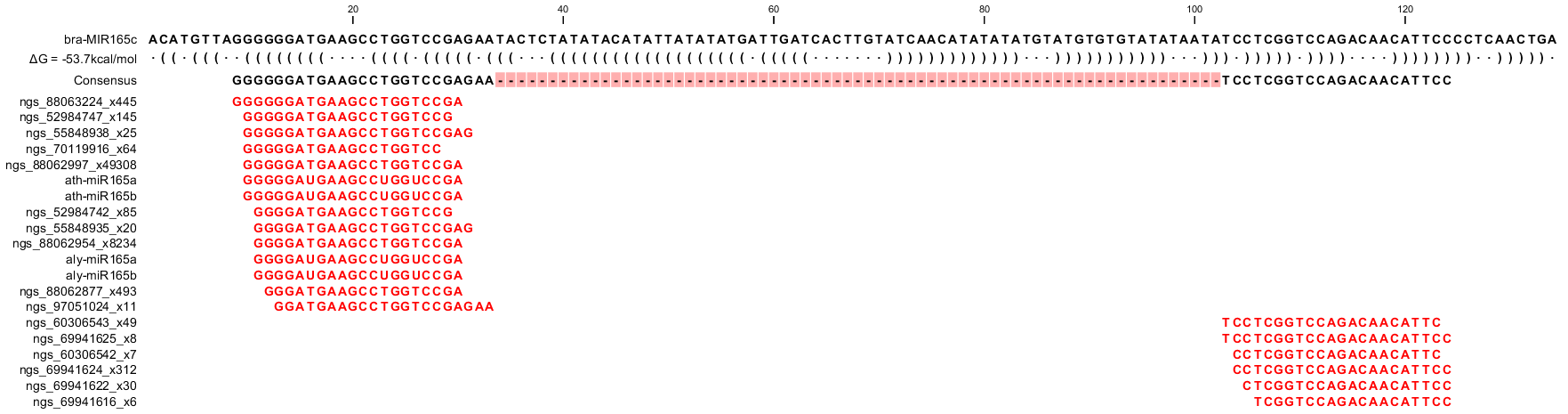
**

**MIR166**
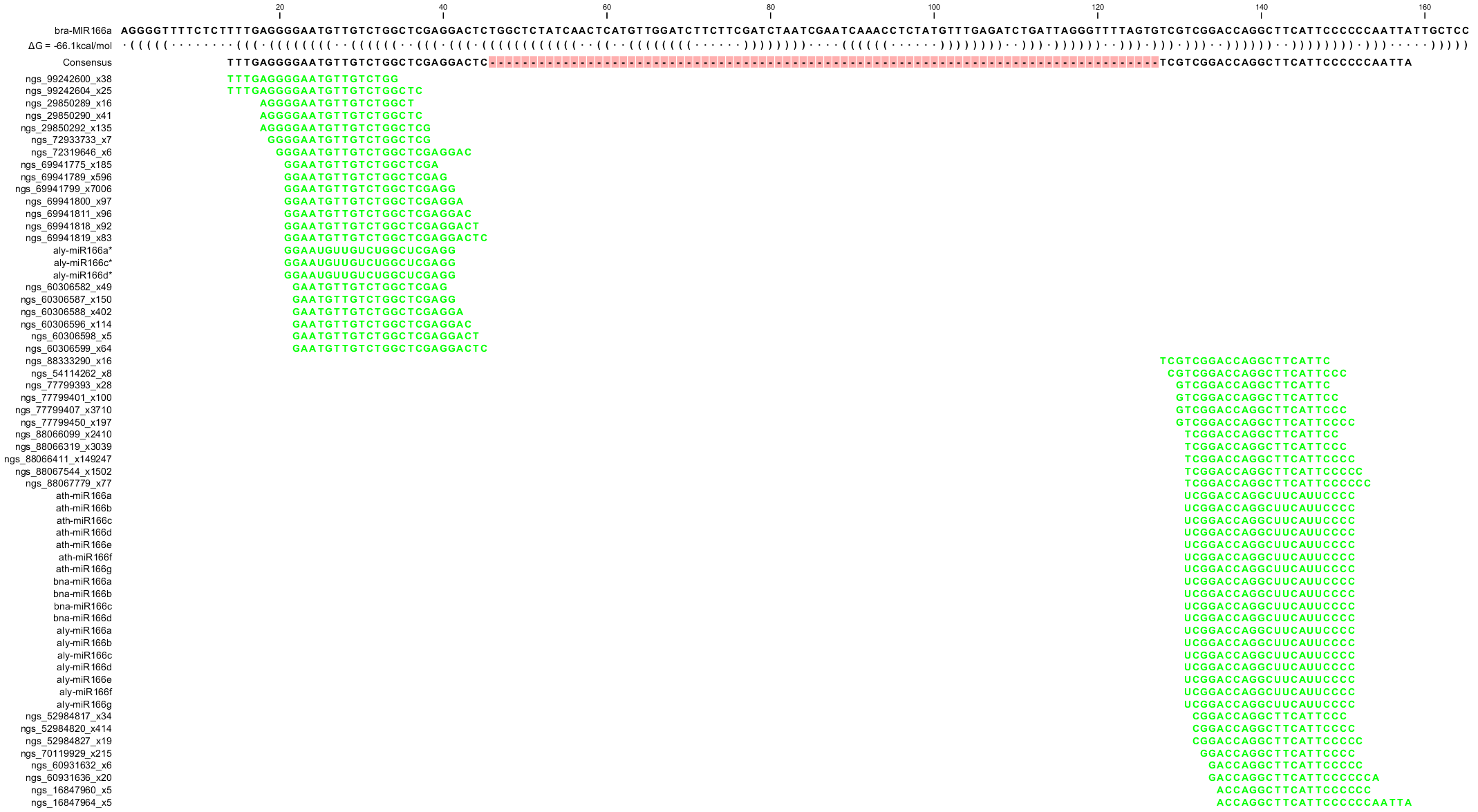

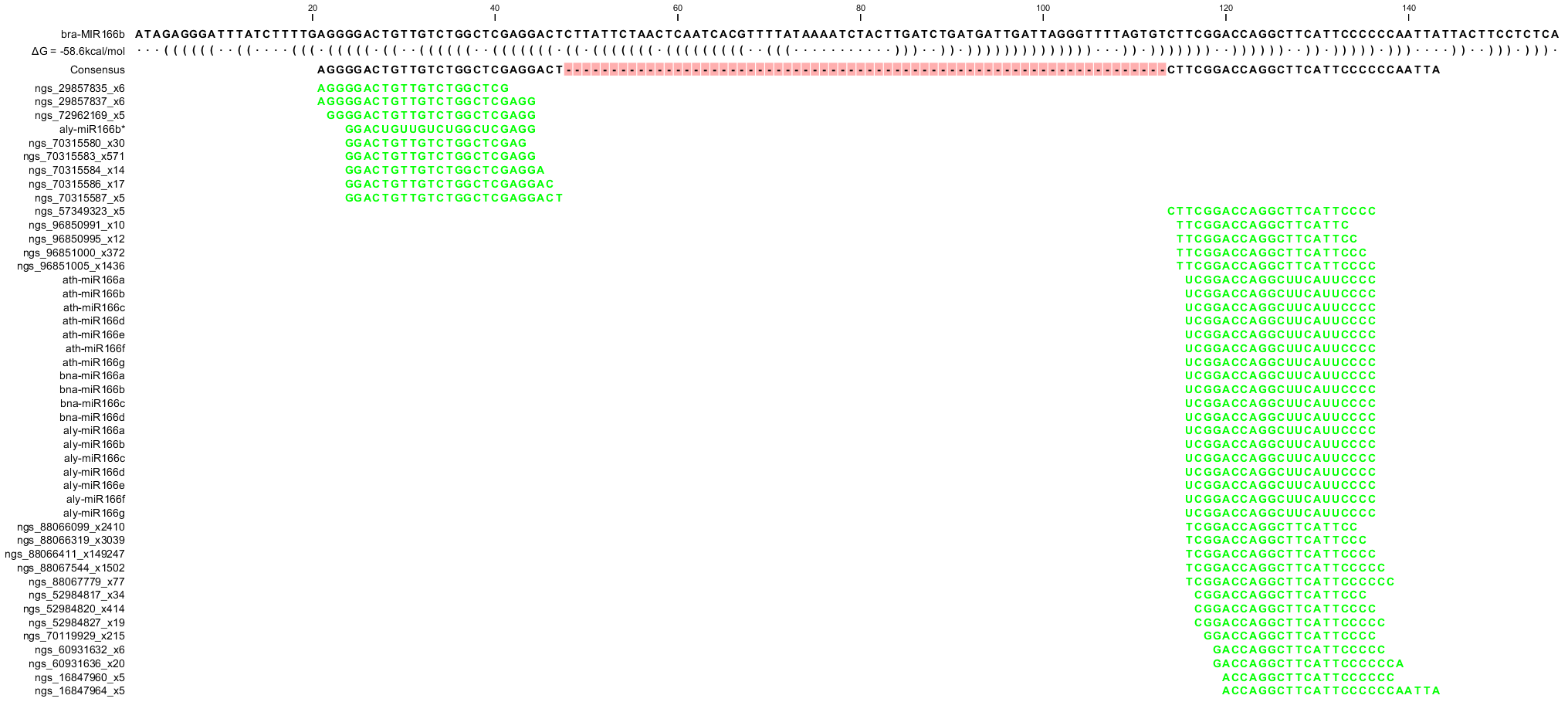

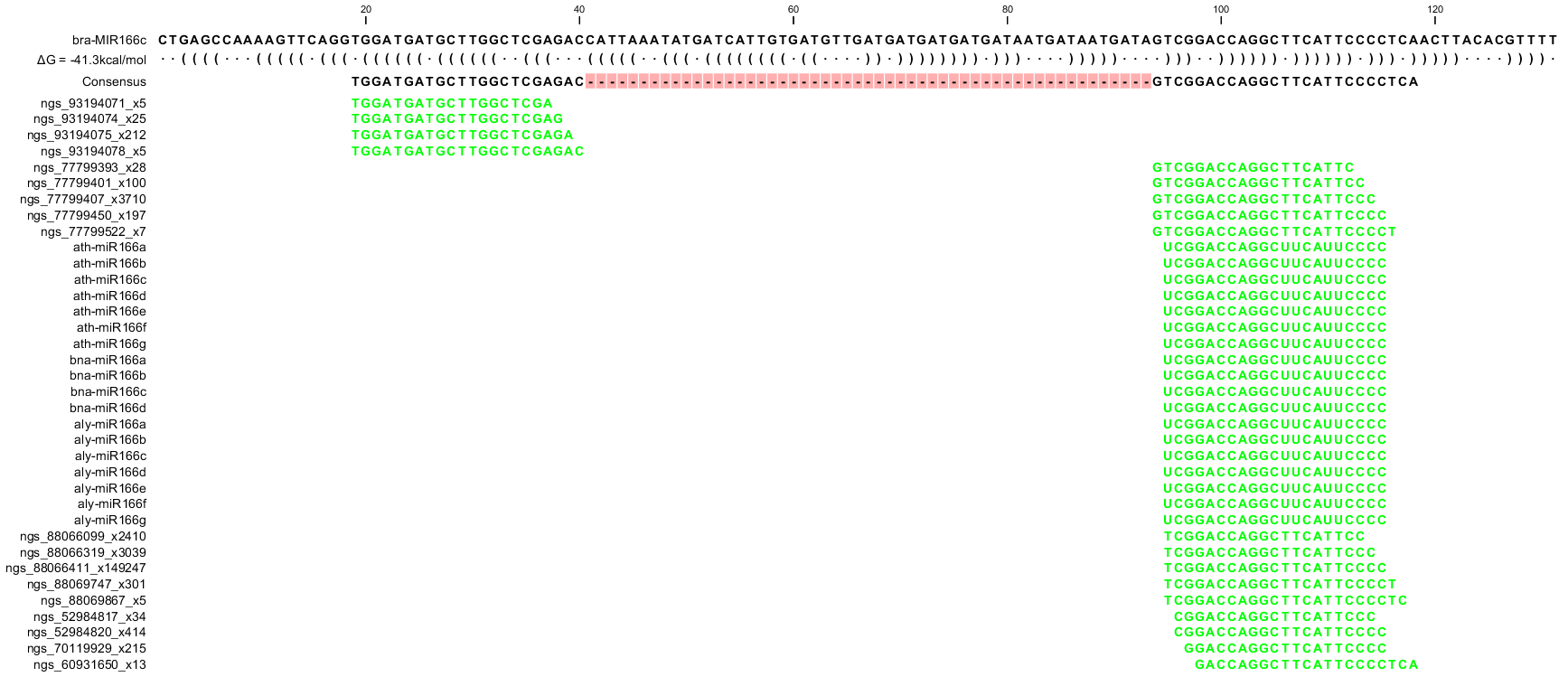

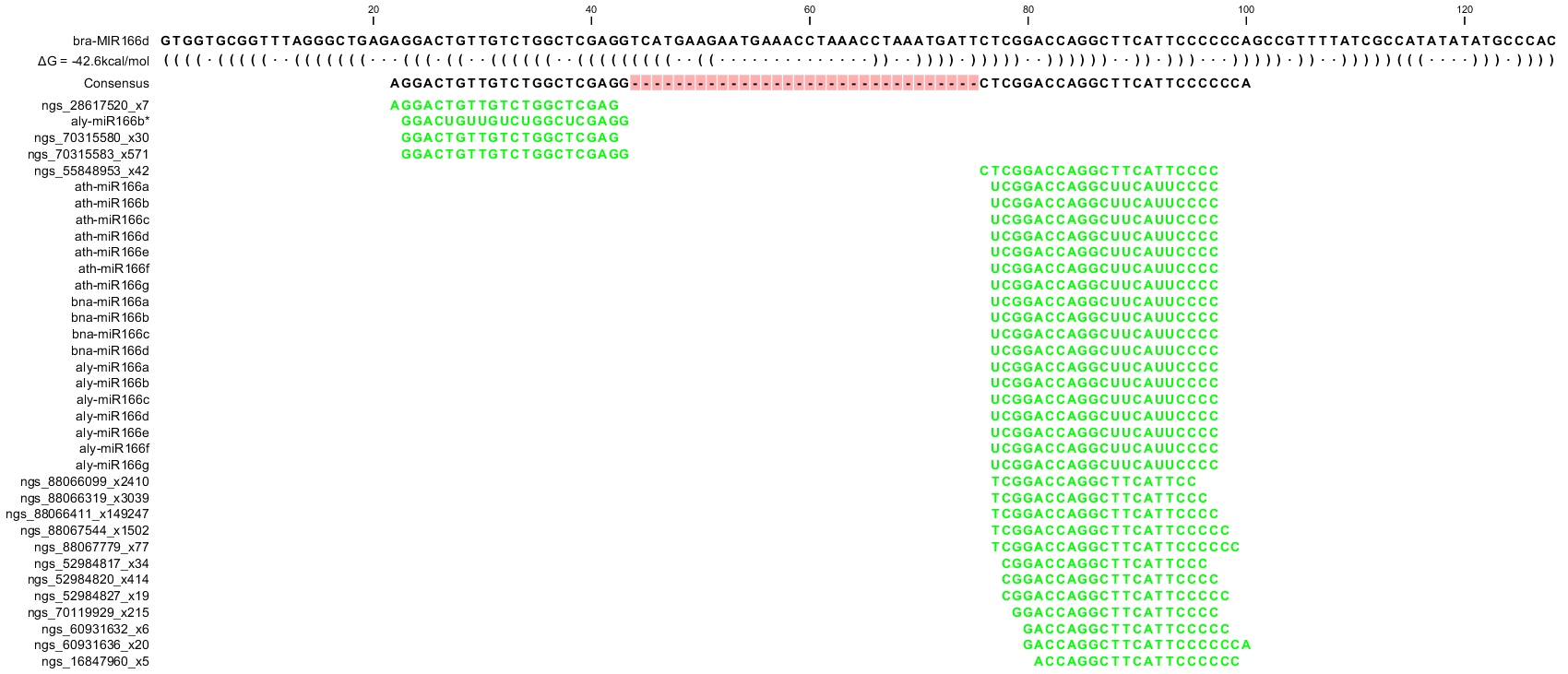

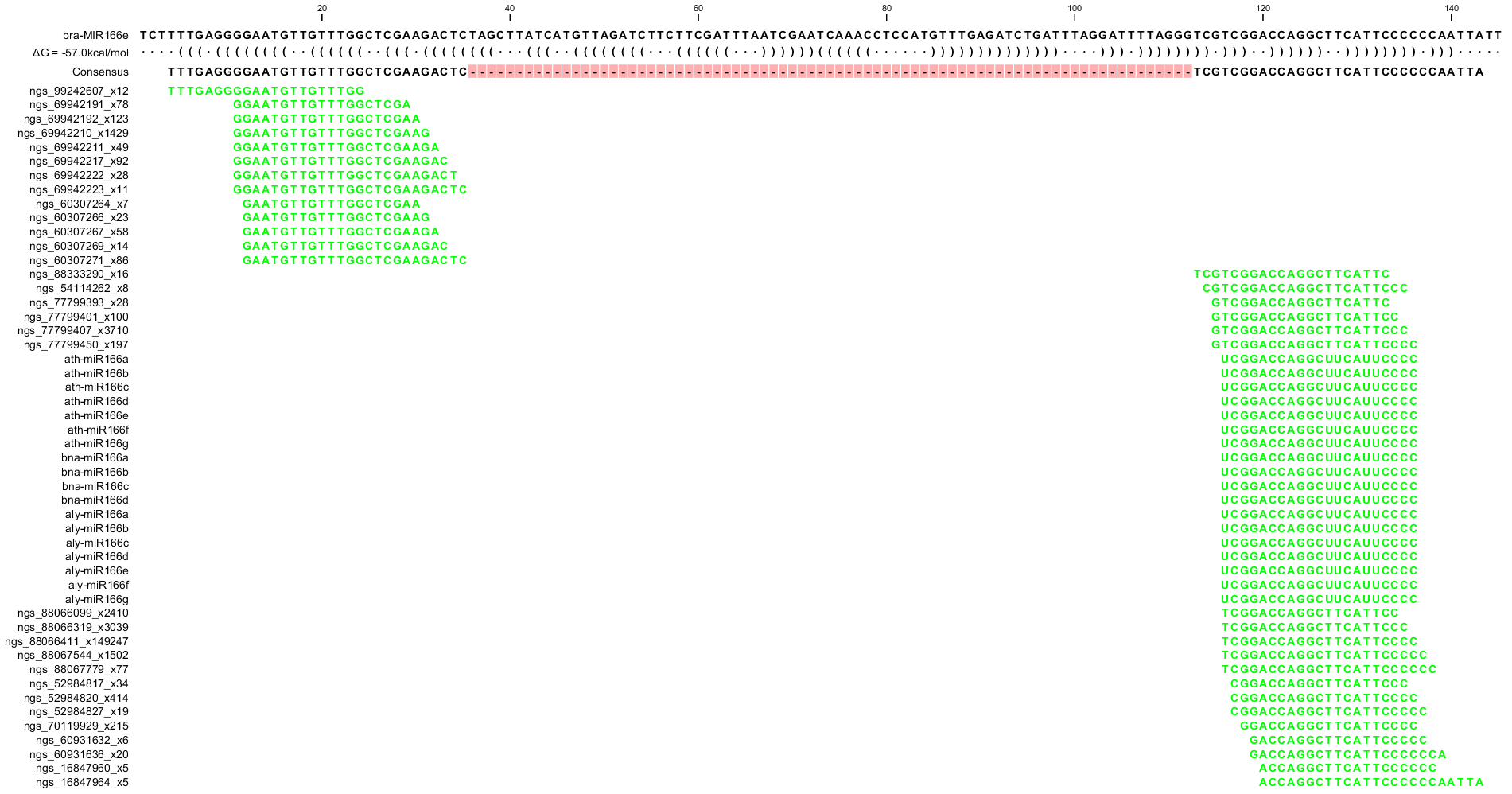

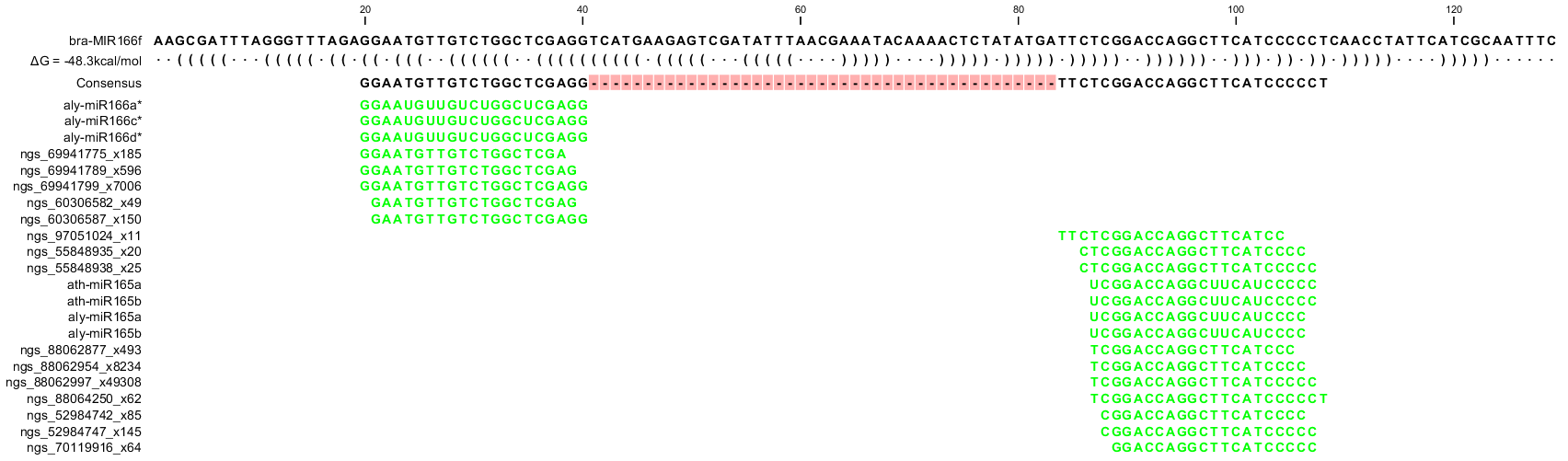


**MIR167**


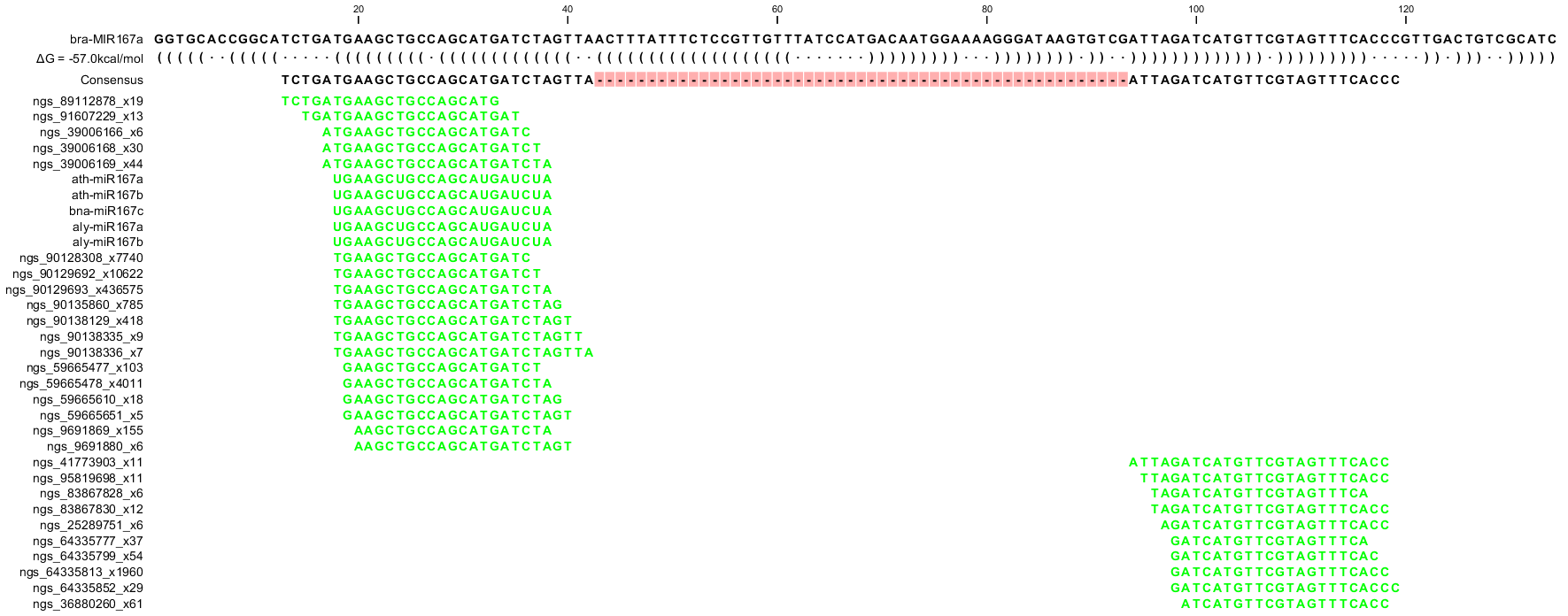


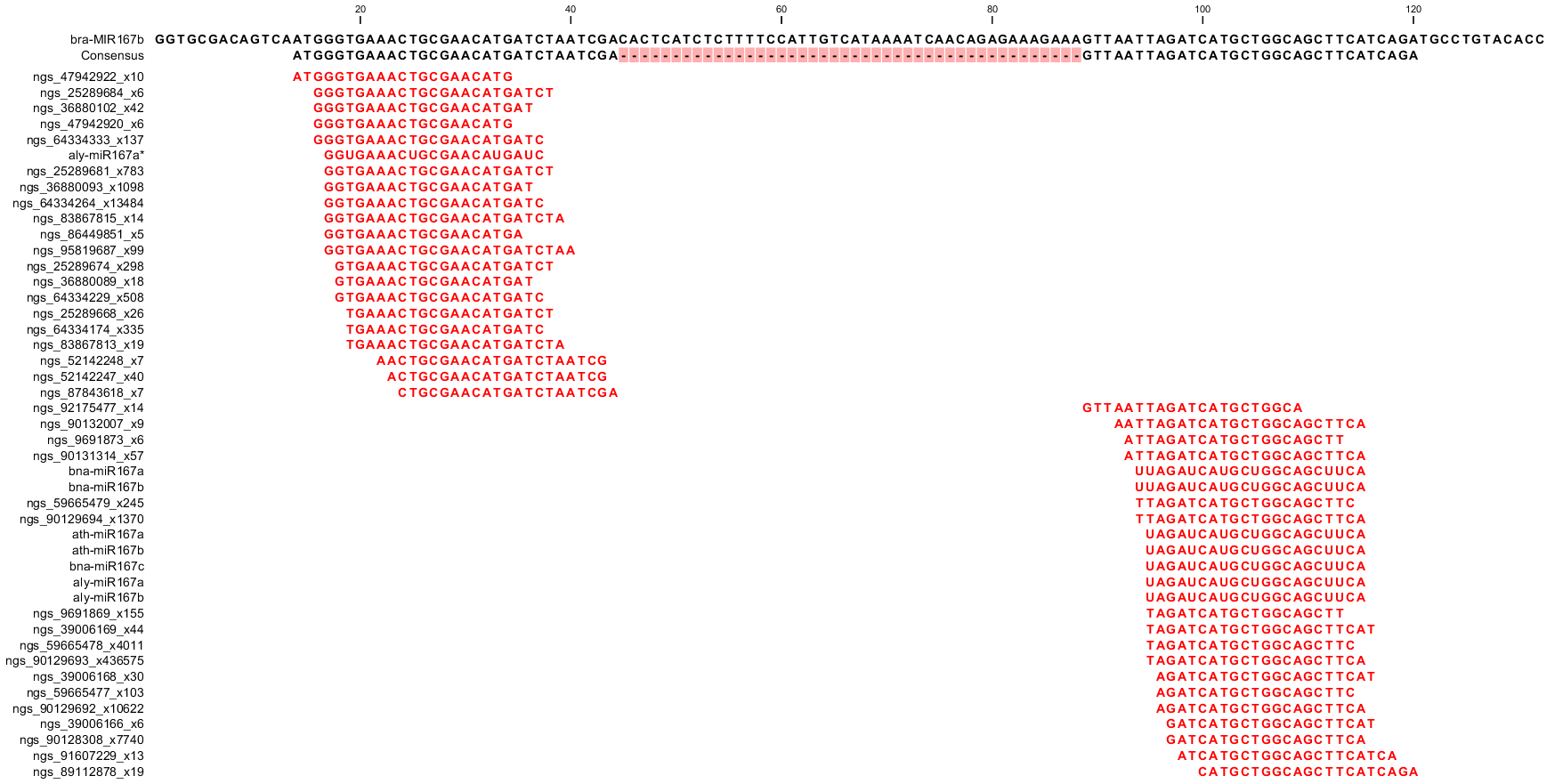

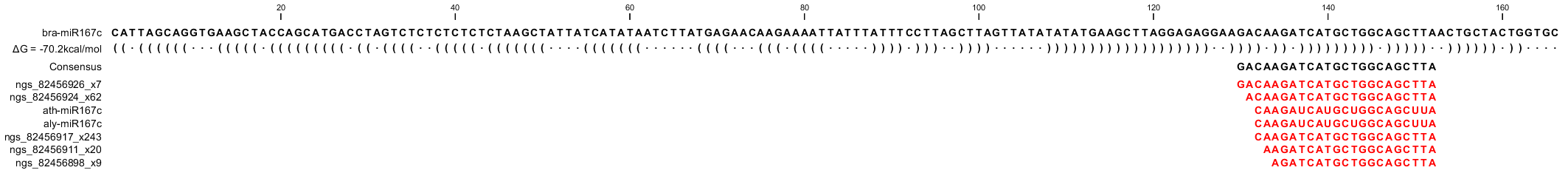

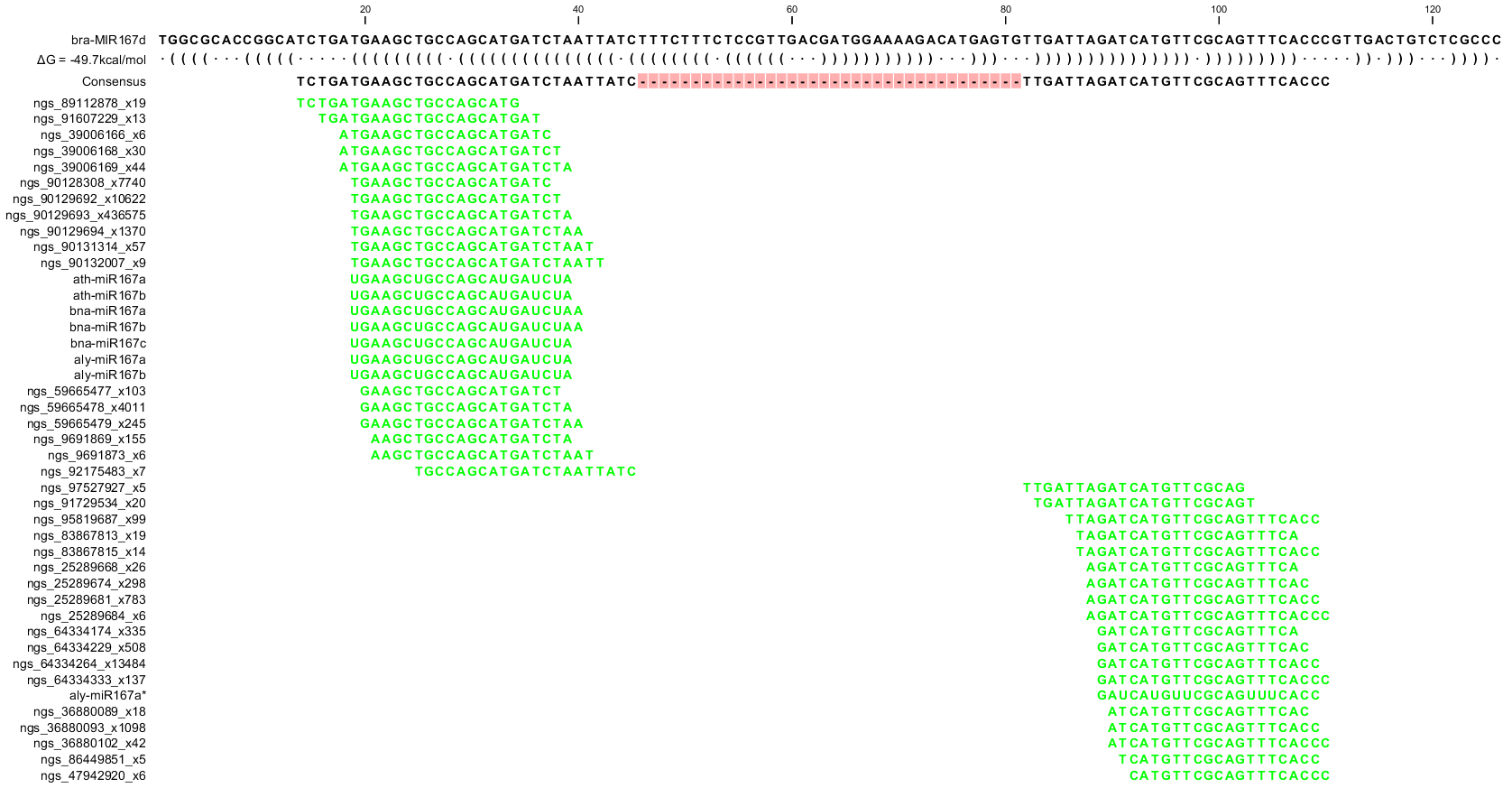


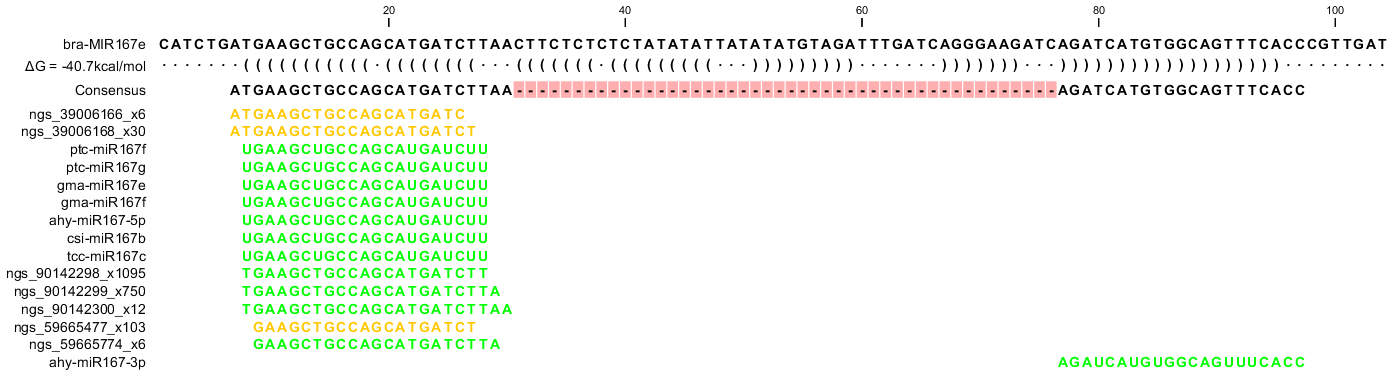


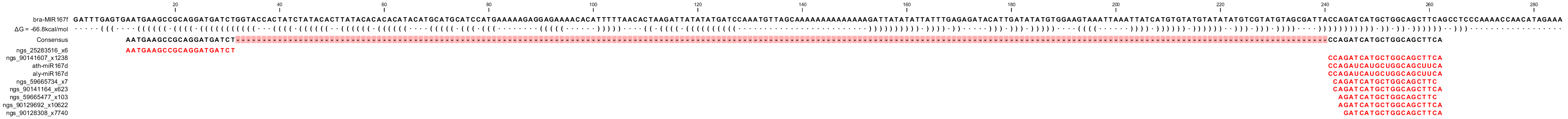


**MIR168**


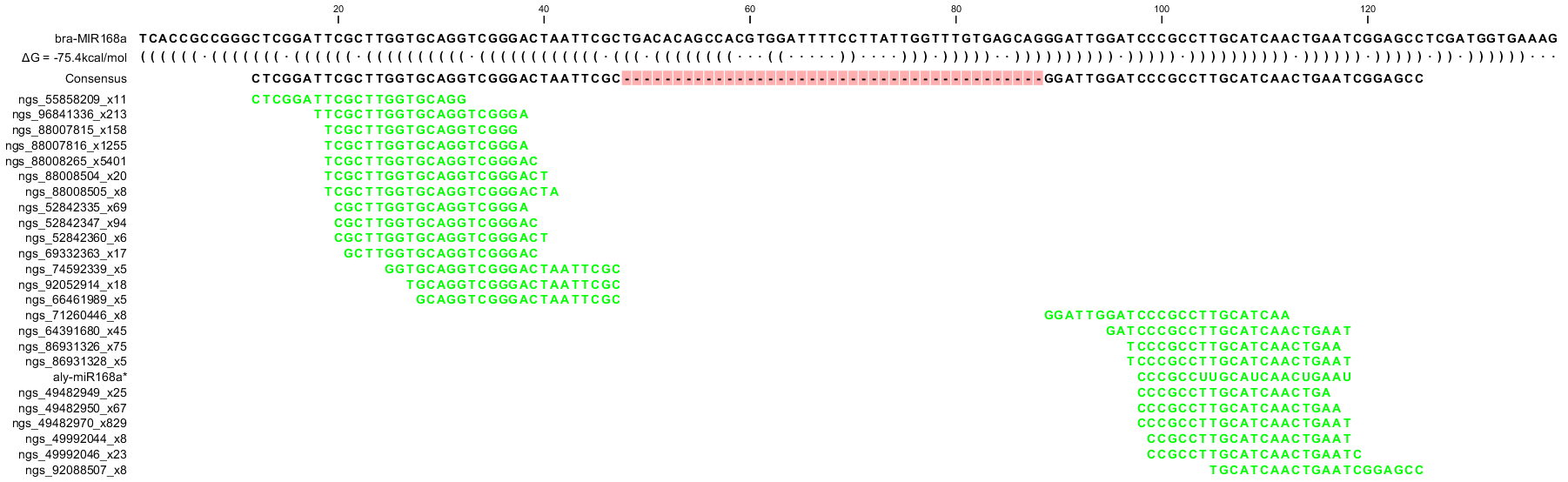

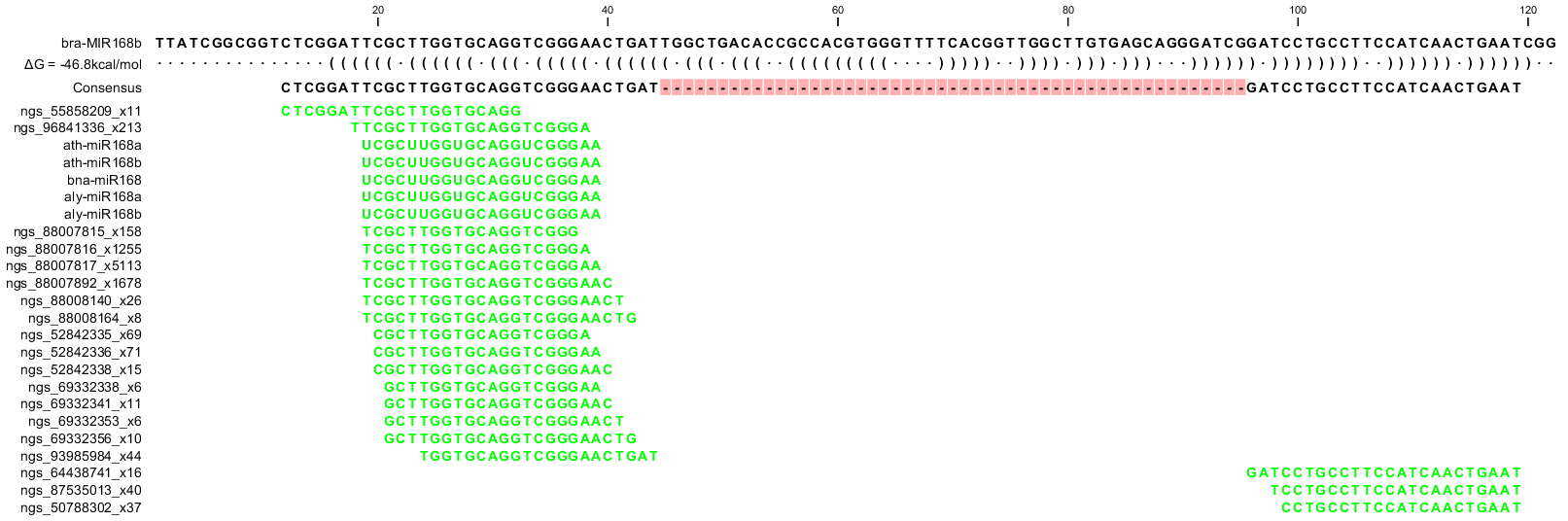

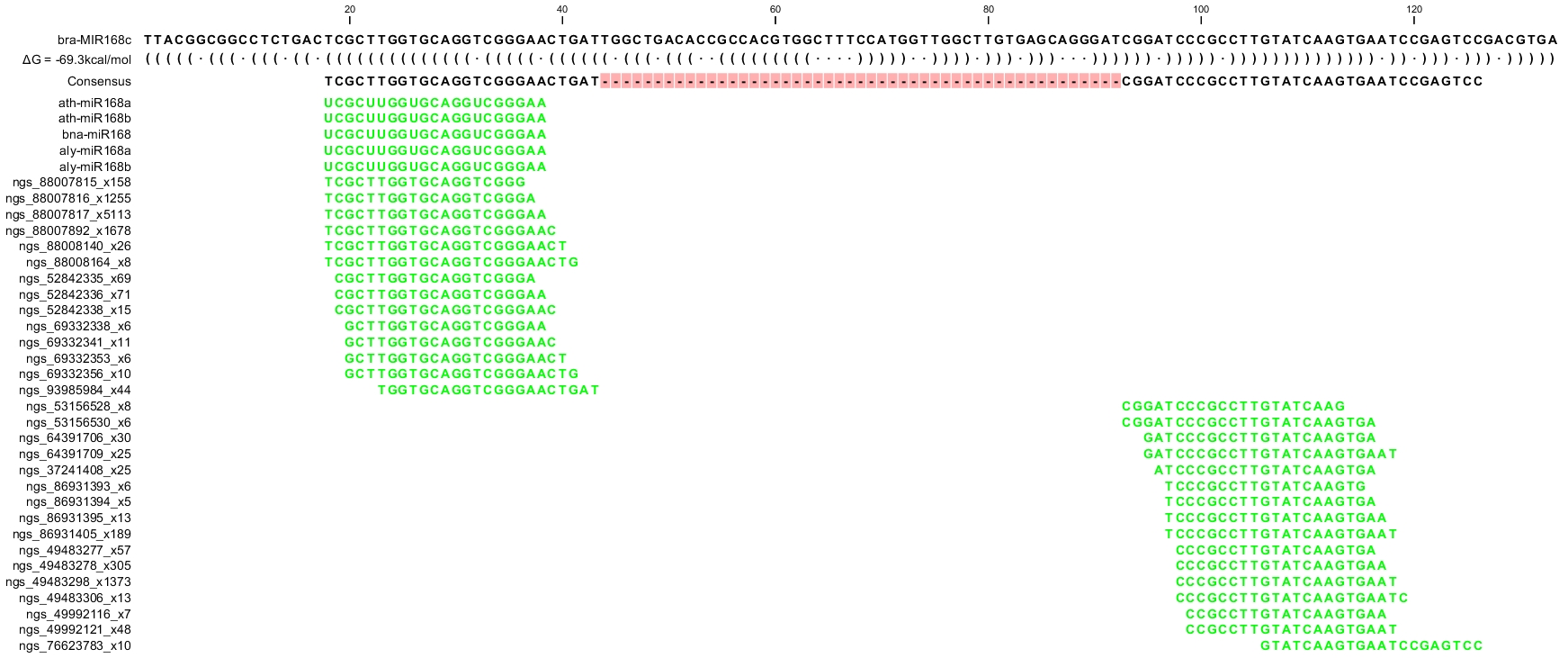

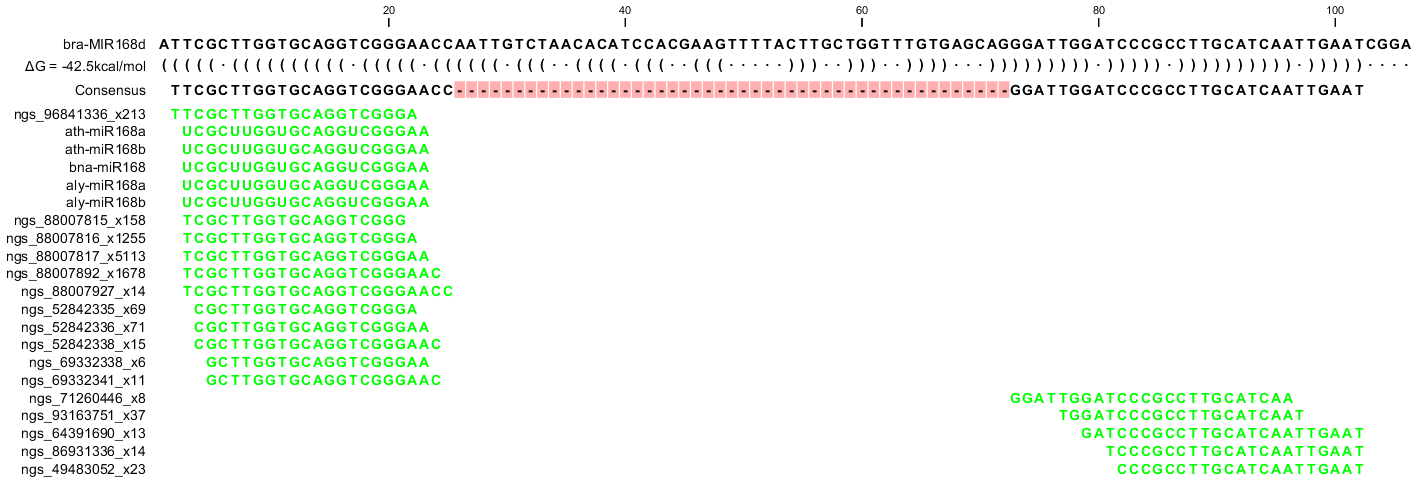


**MIR169**


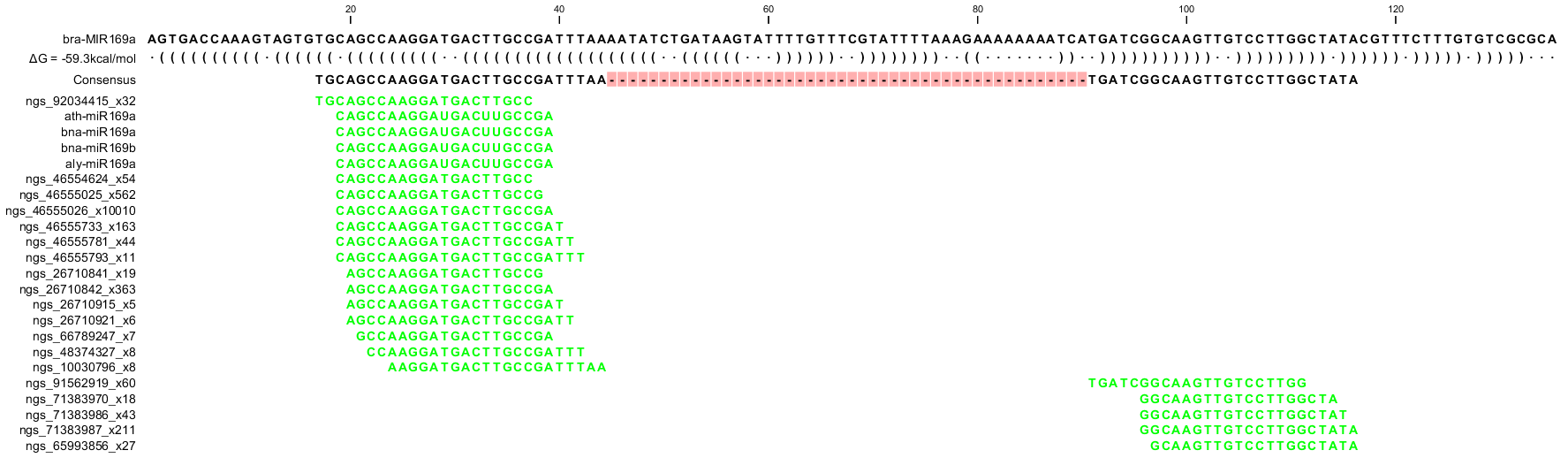

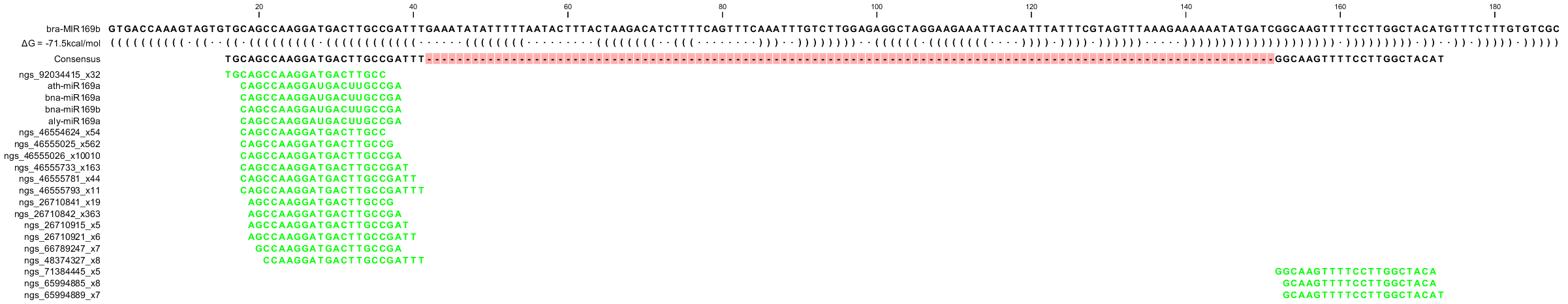

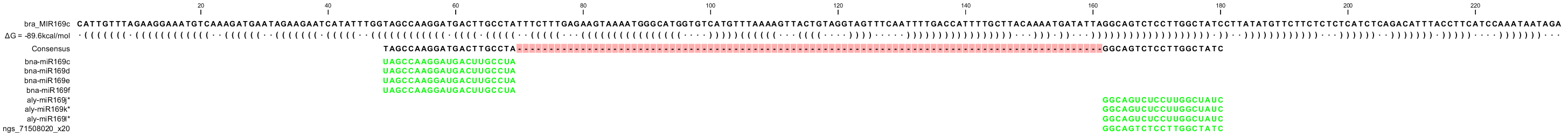


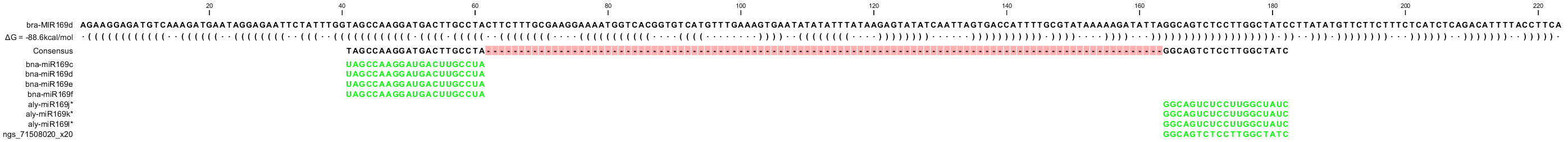

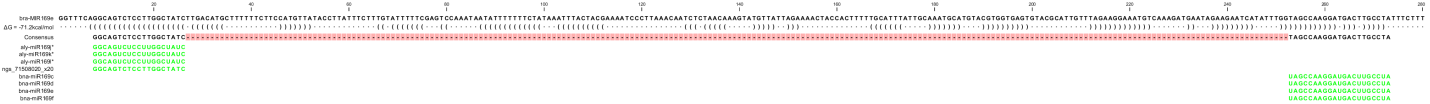

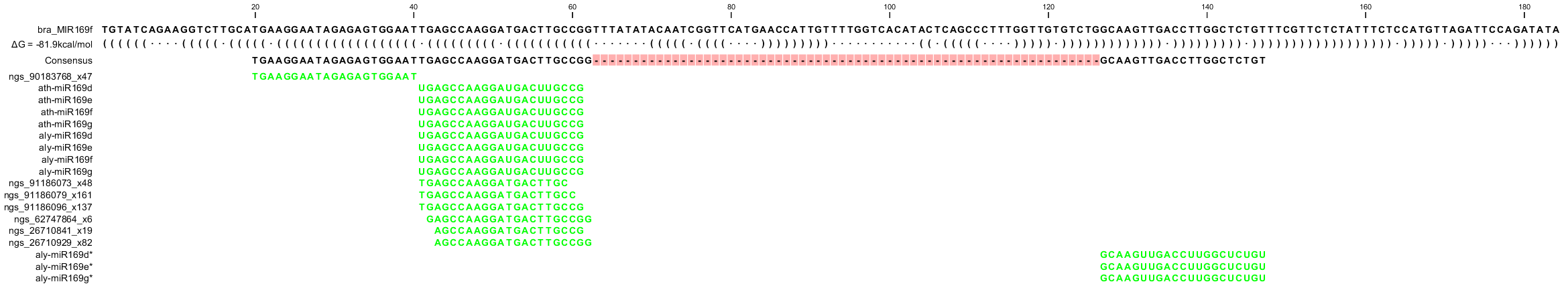

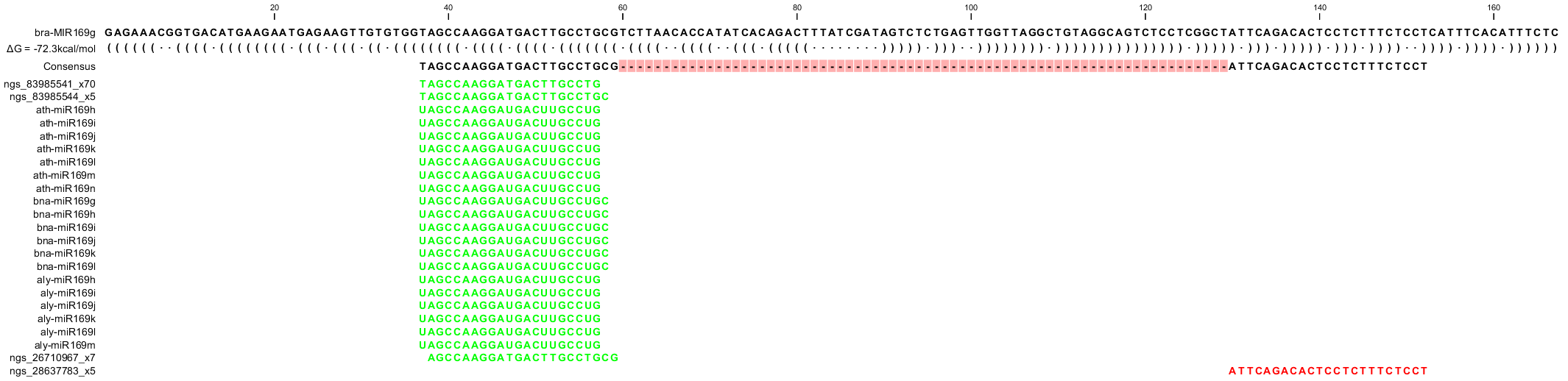

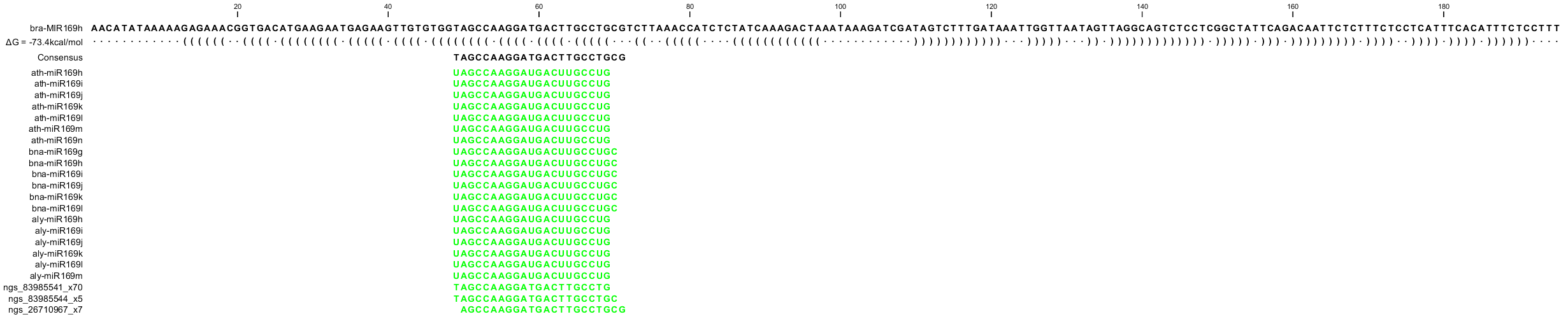

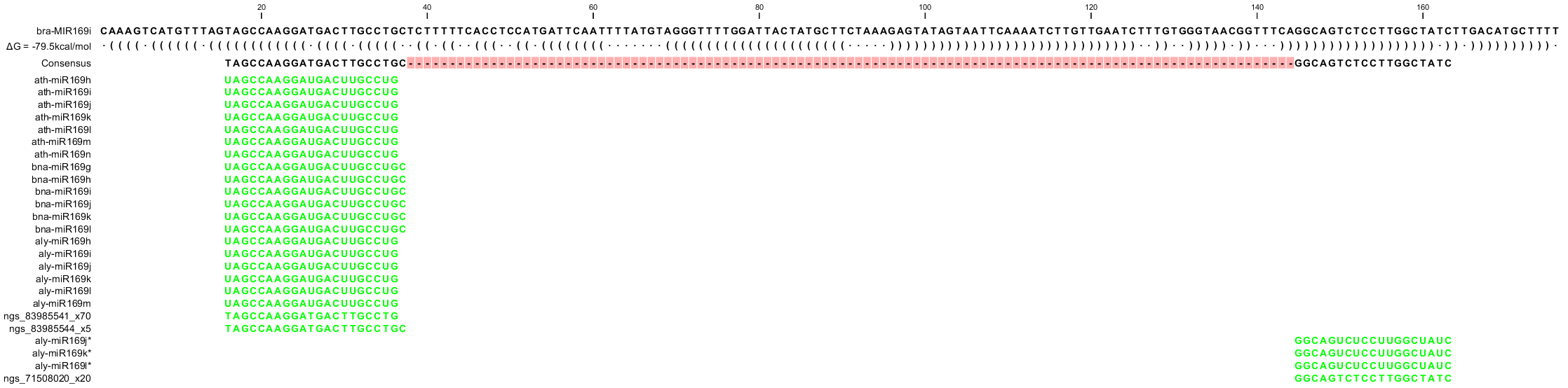


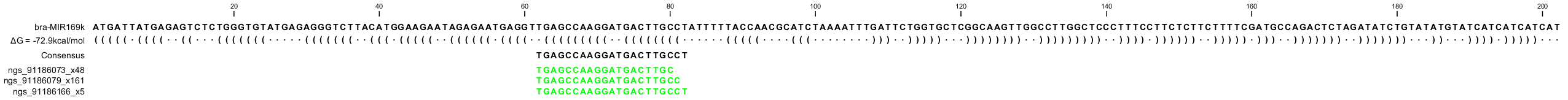


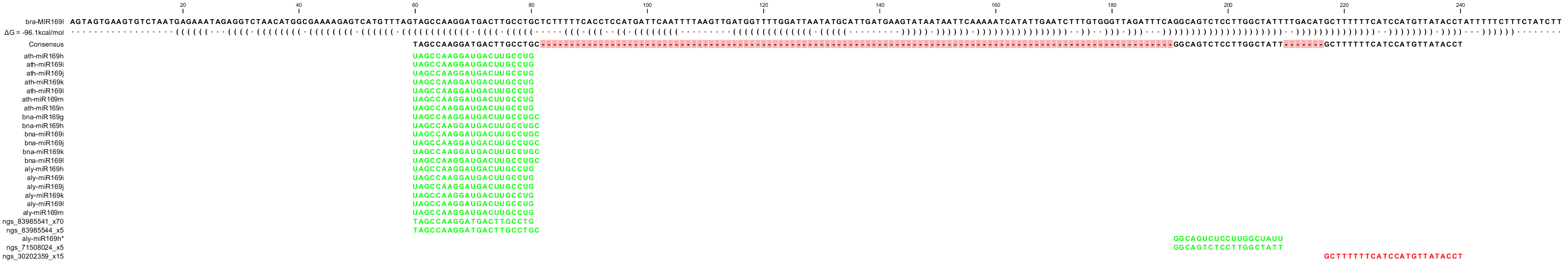


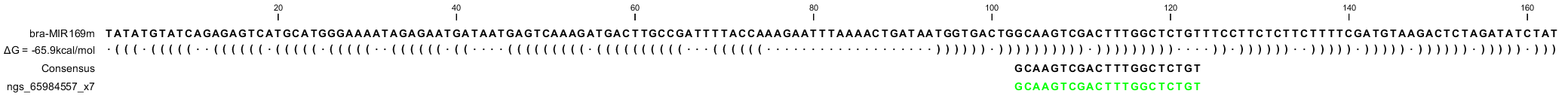


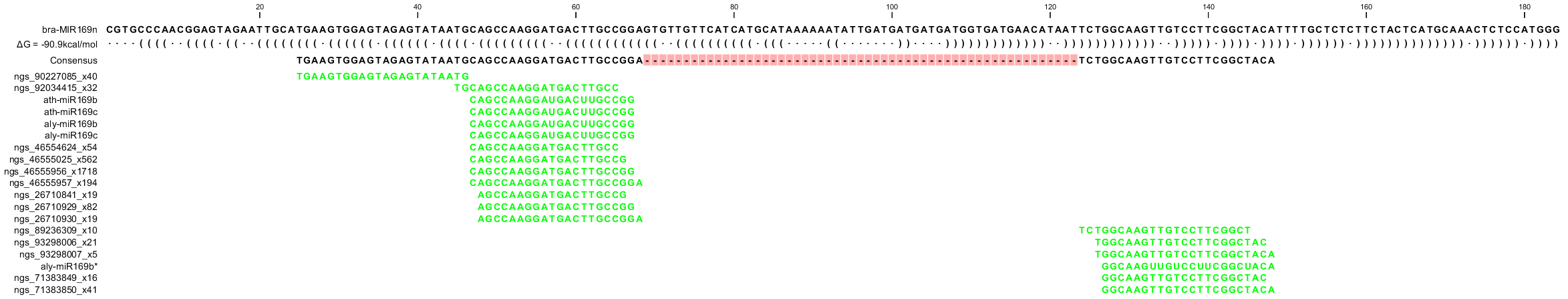

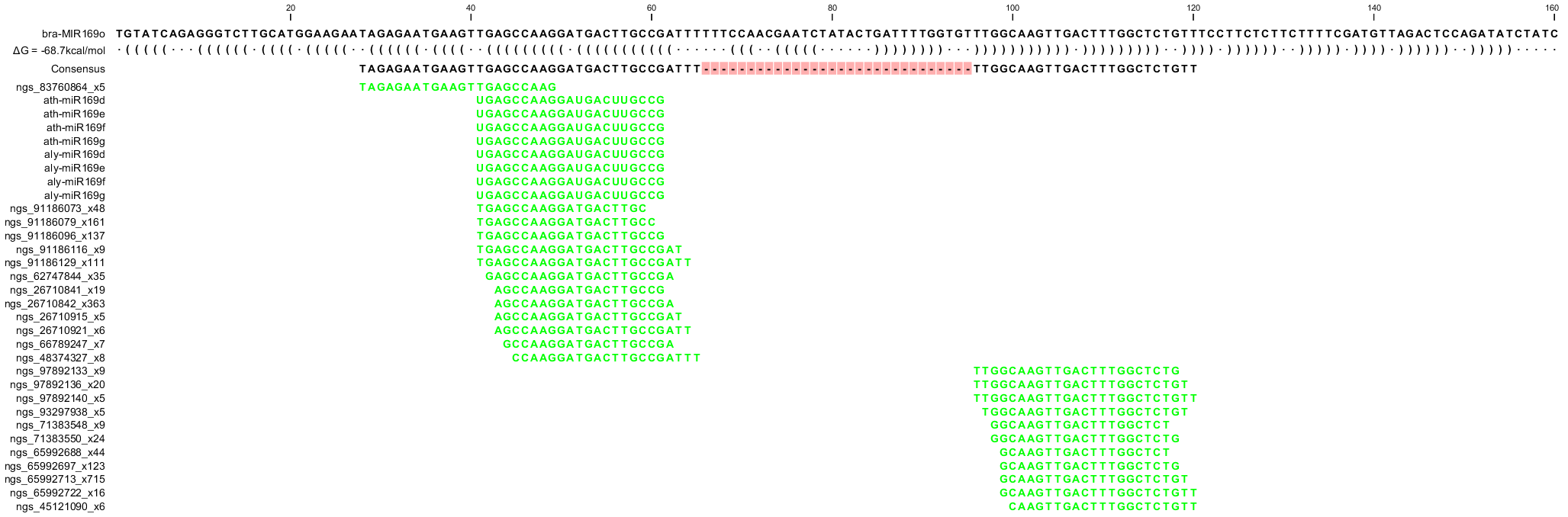

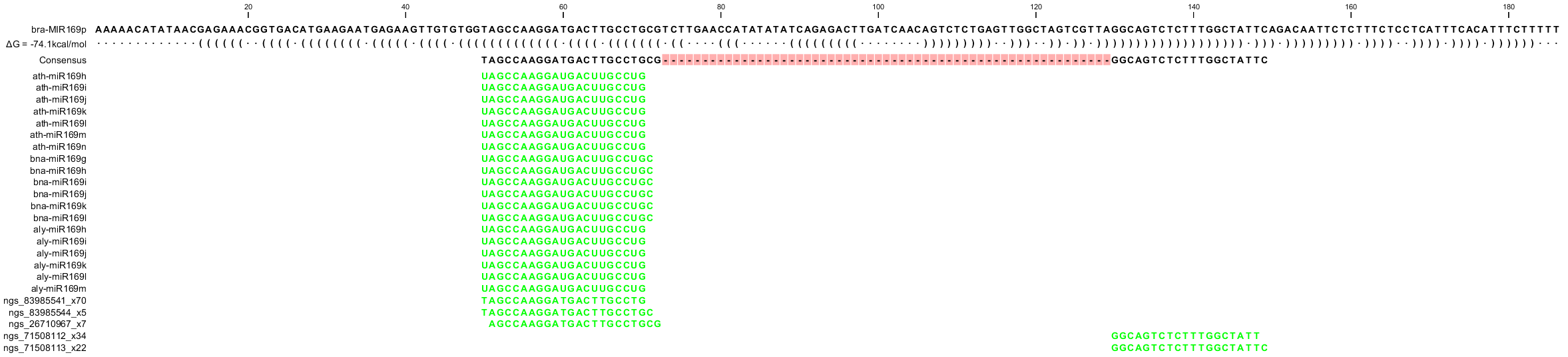

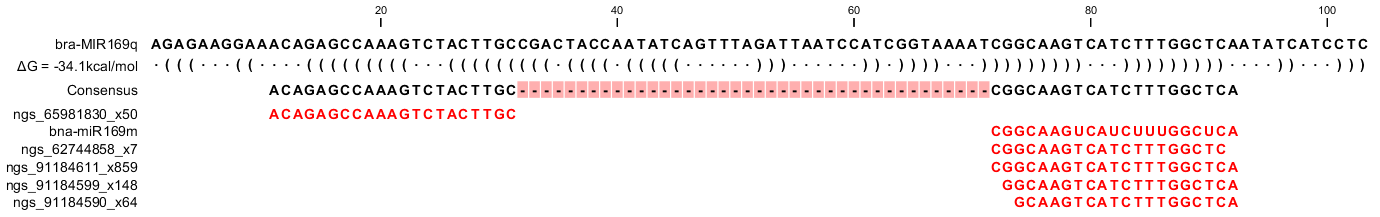


**
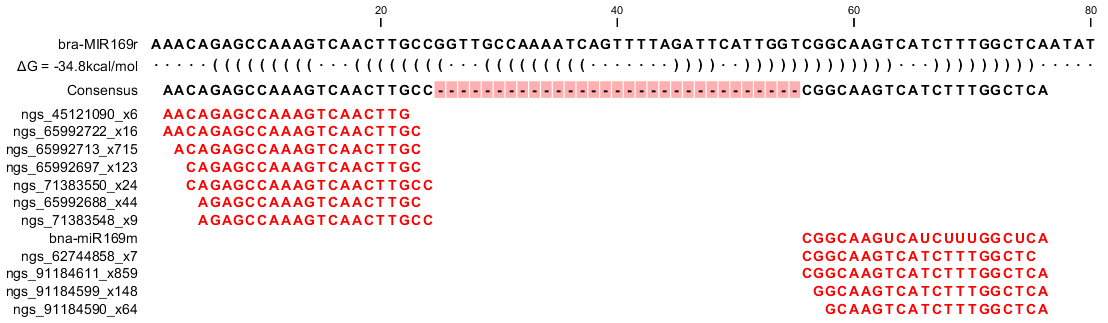
**

**
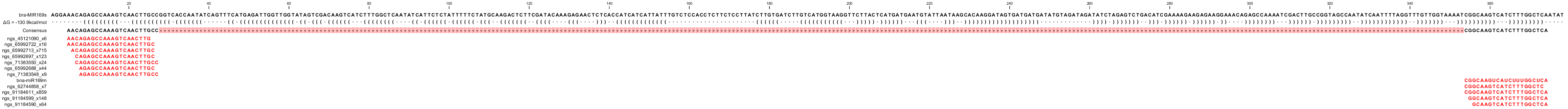
**

**MIR171**


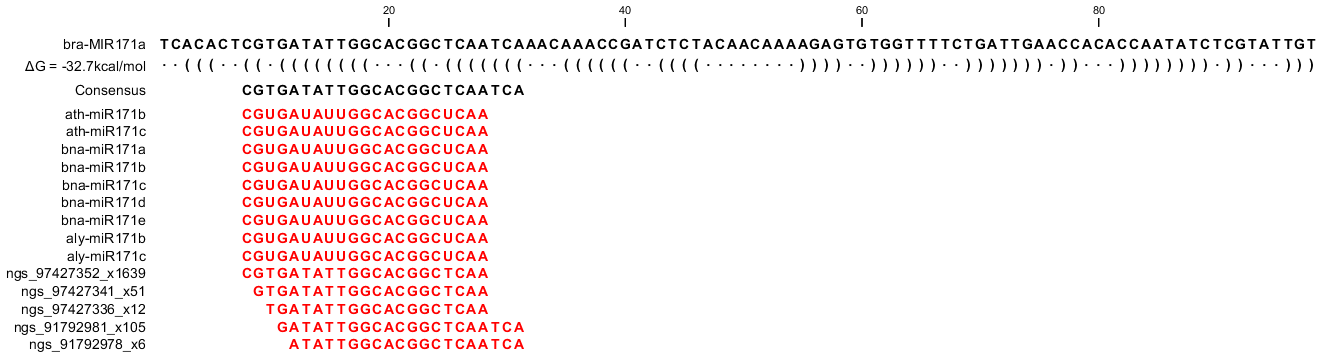

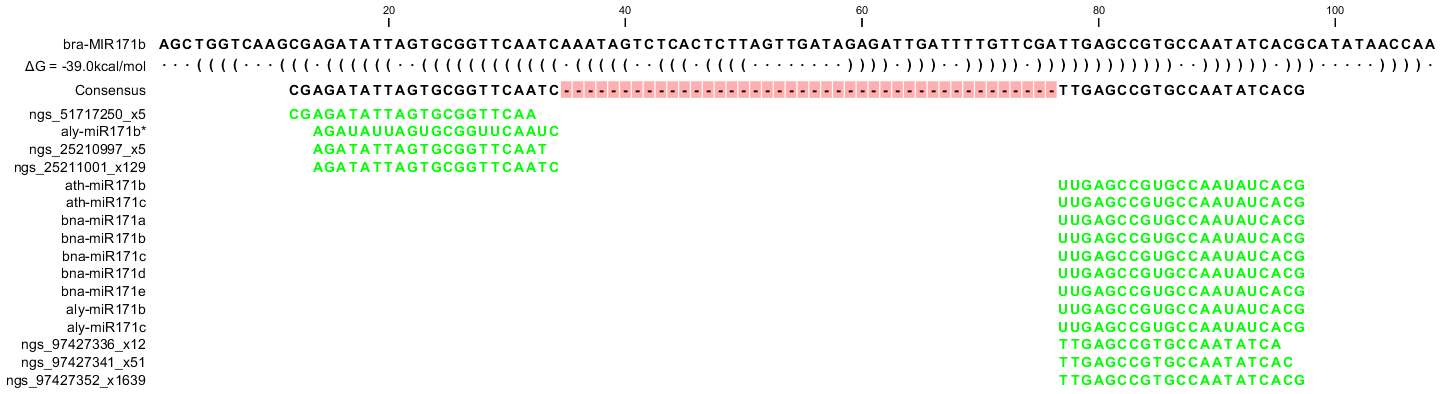

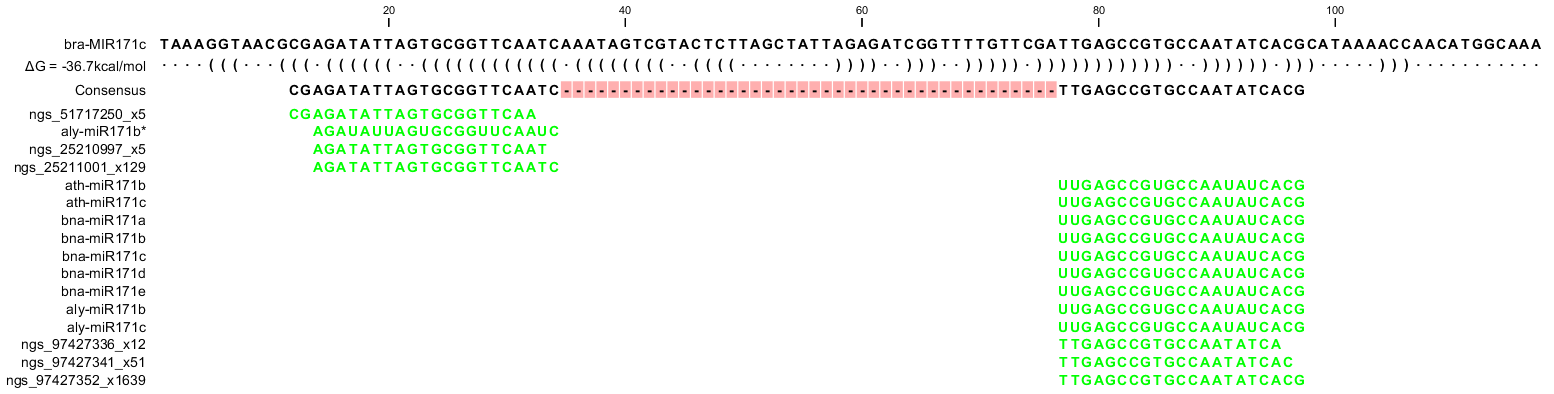

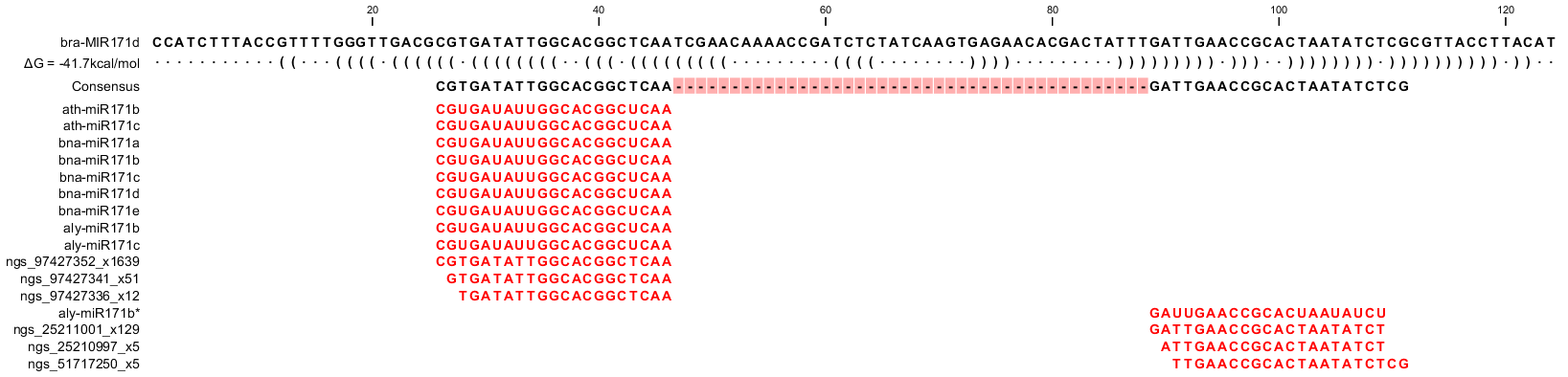

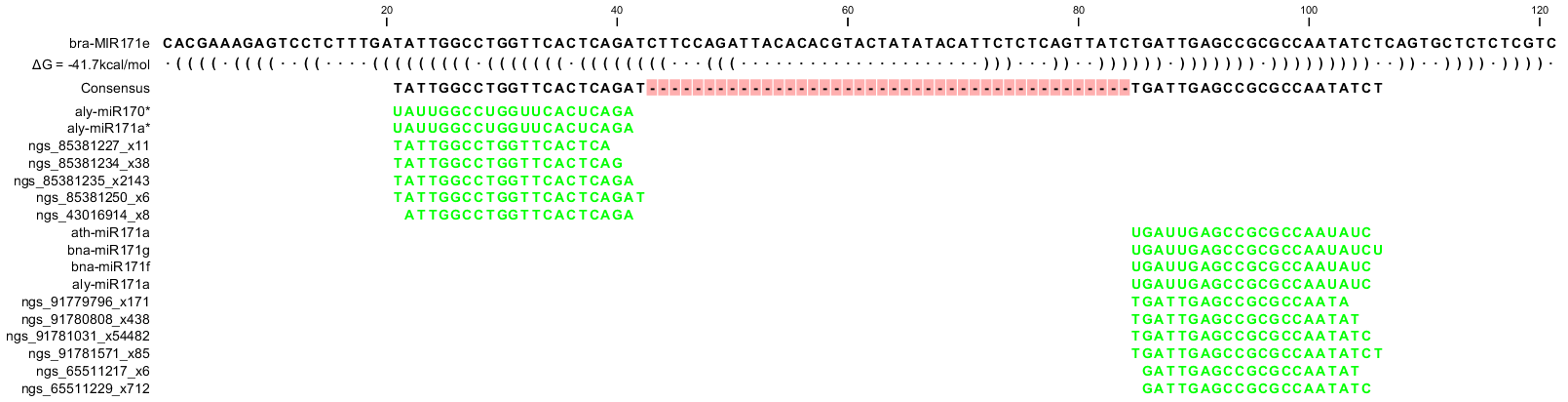

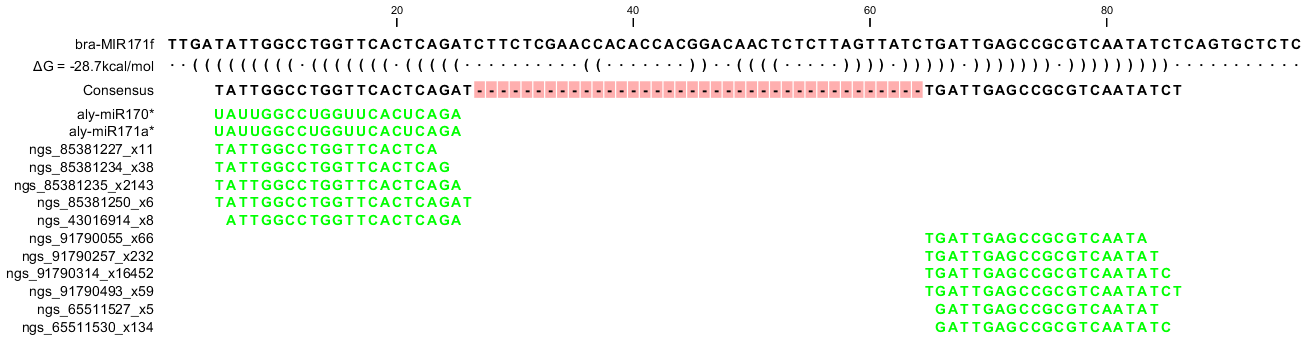

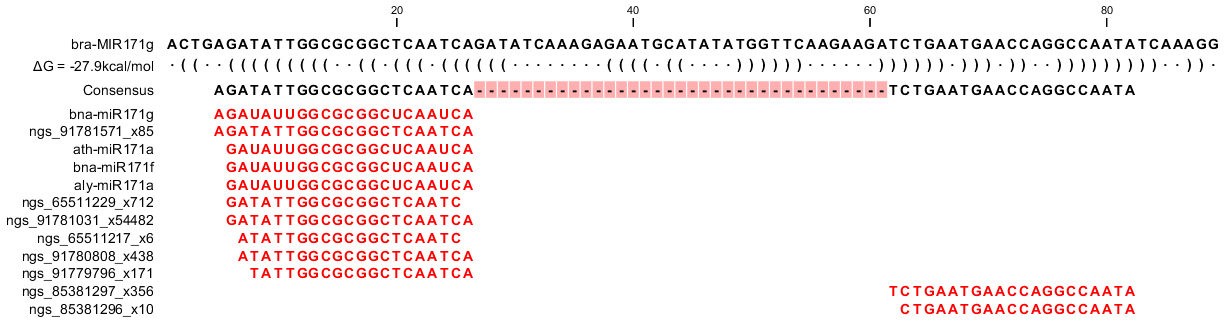


**MIR172**


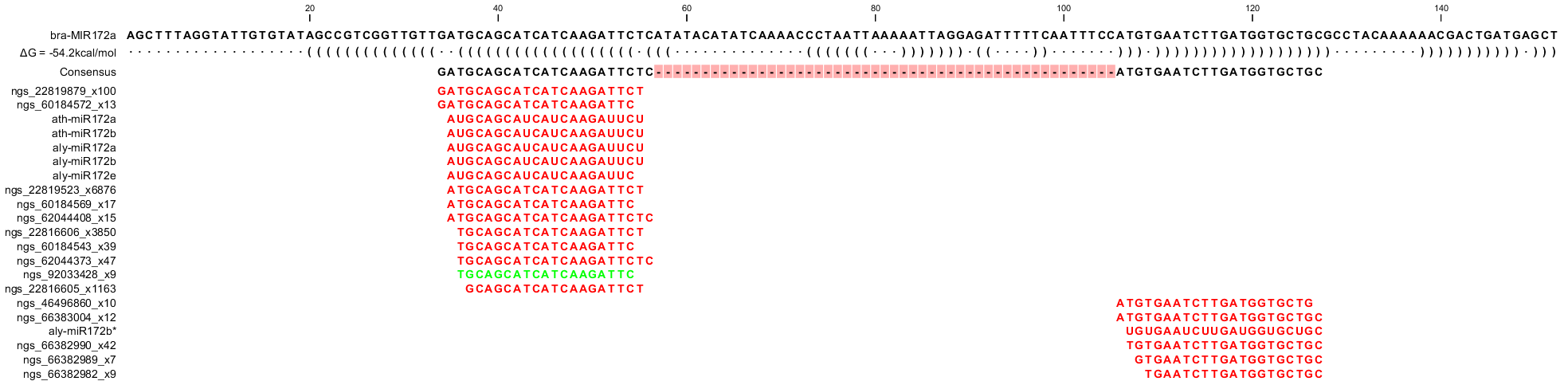

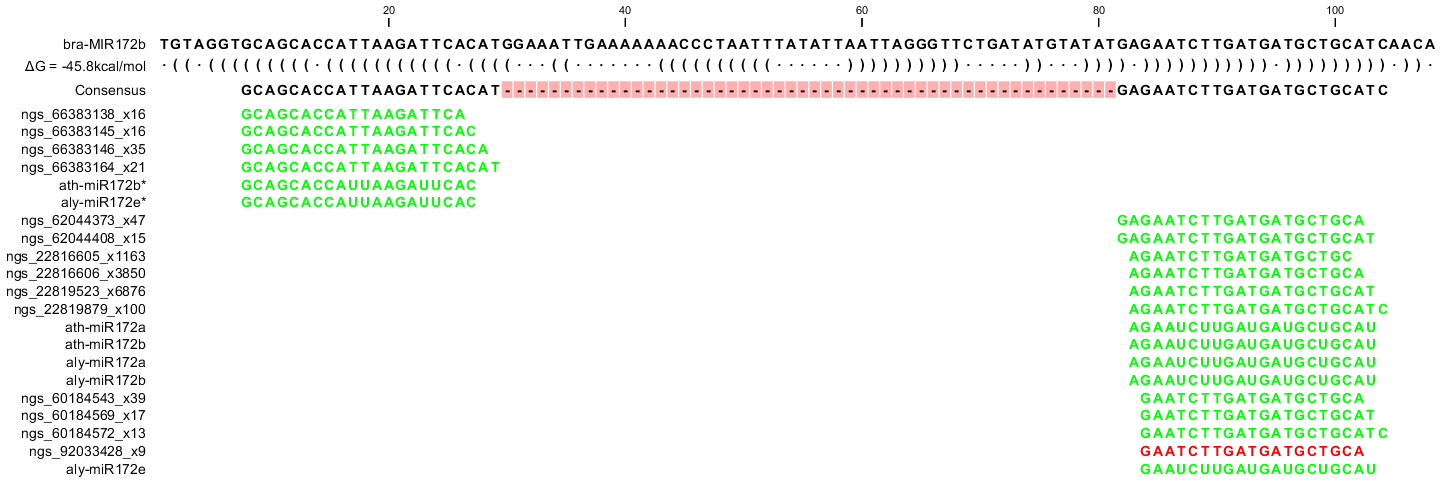

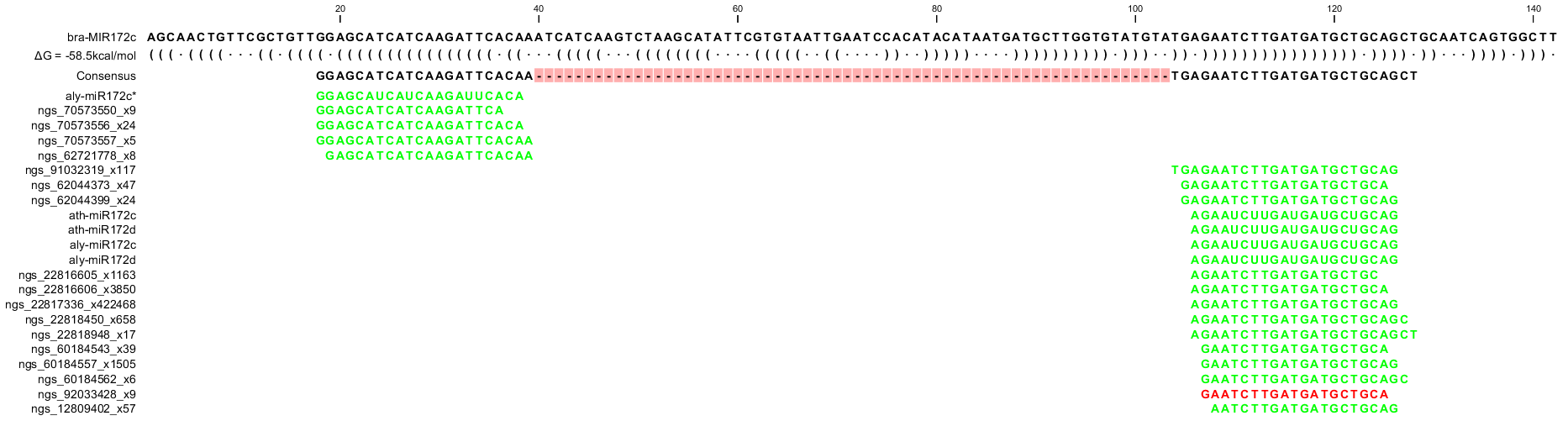

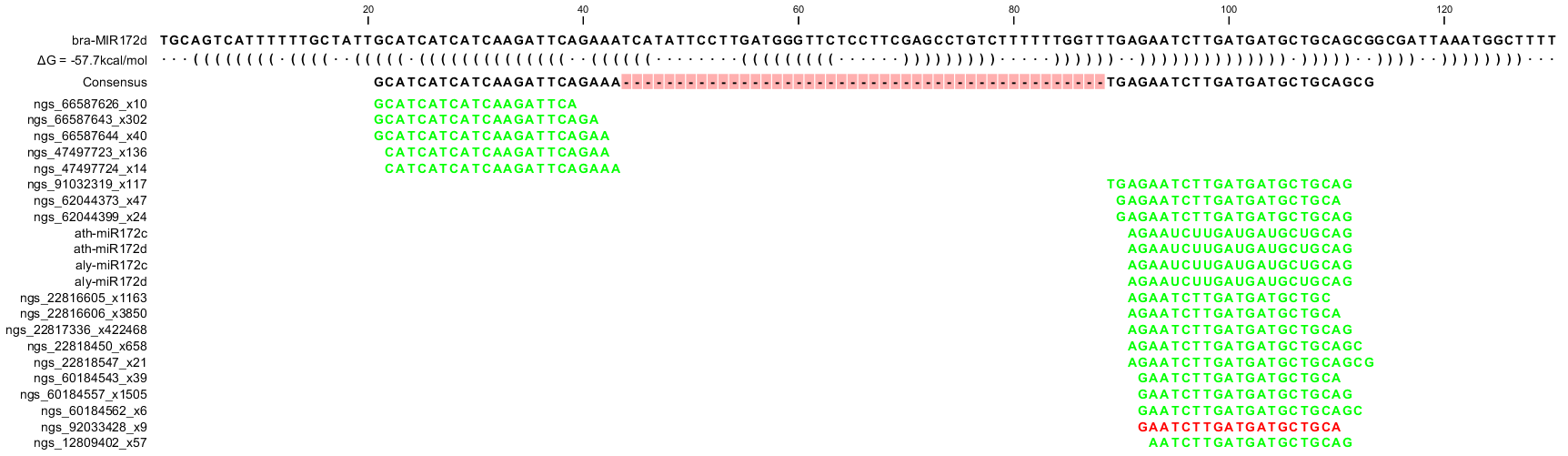

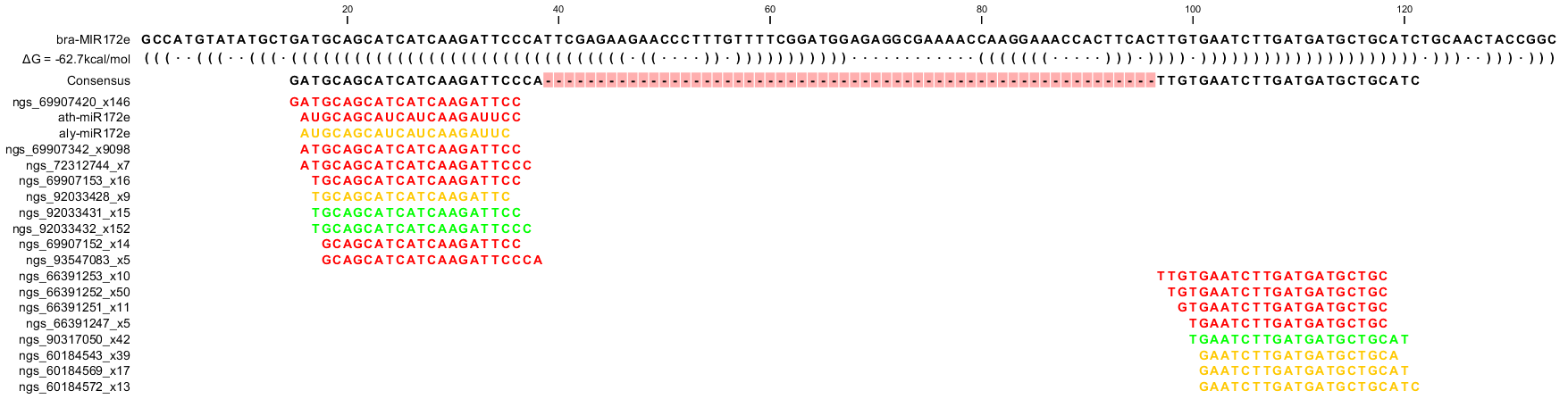

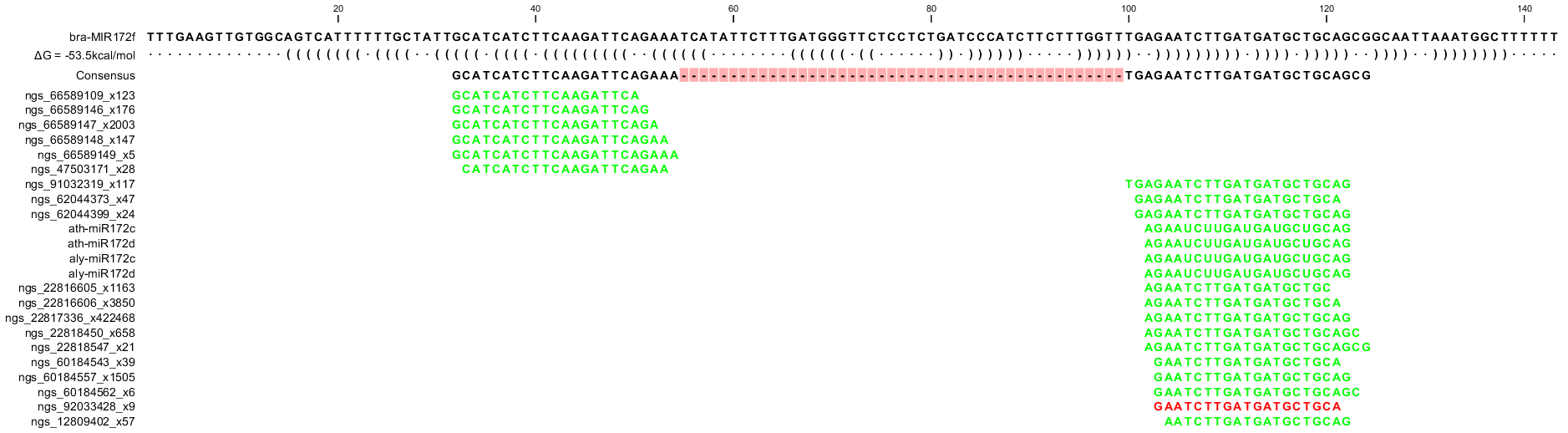

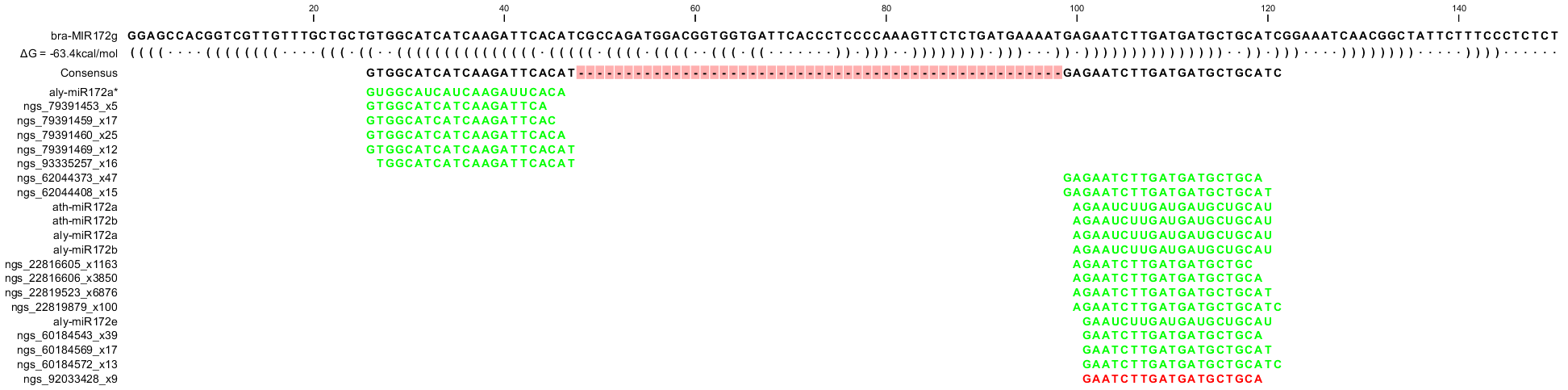

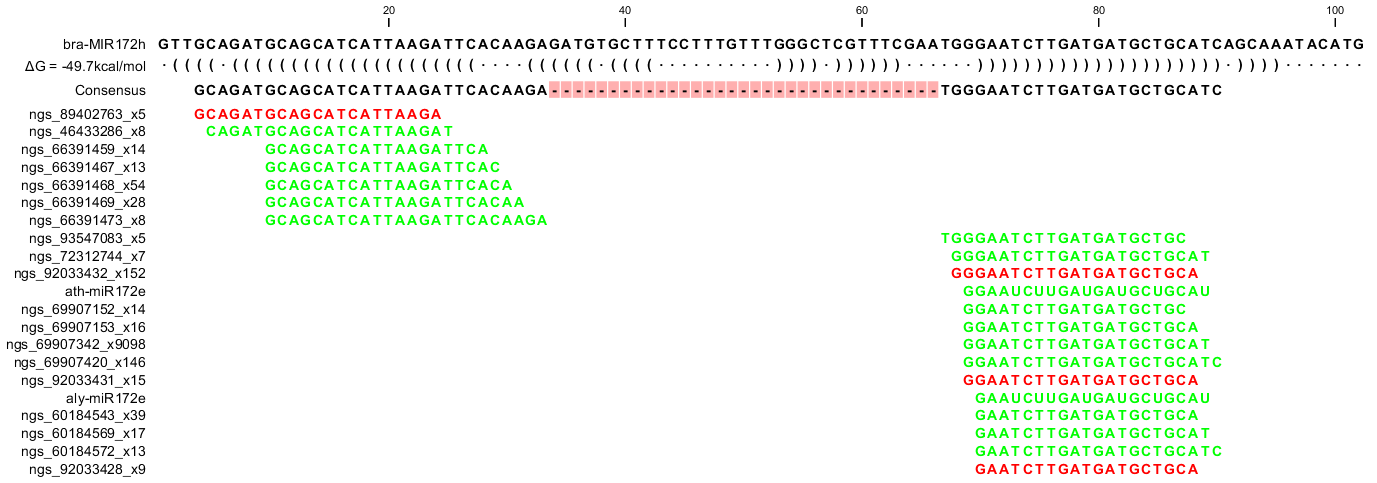

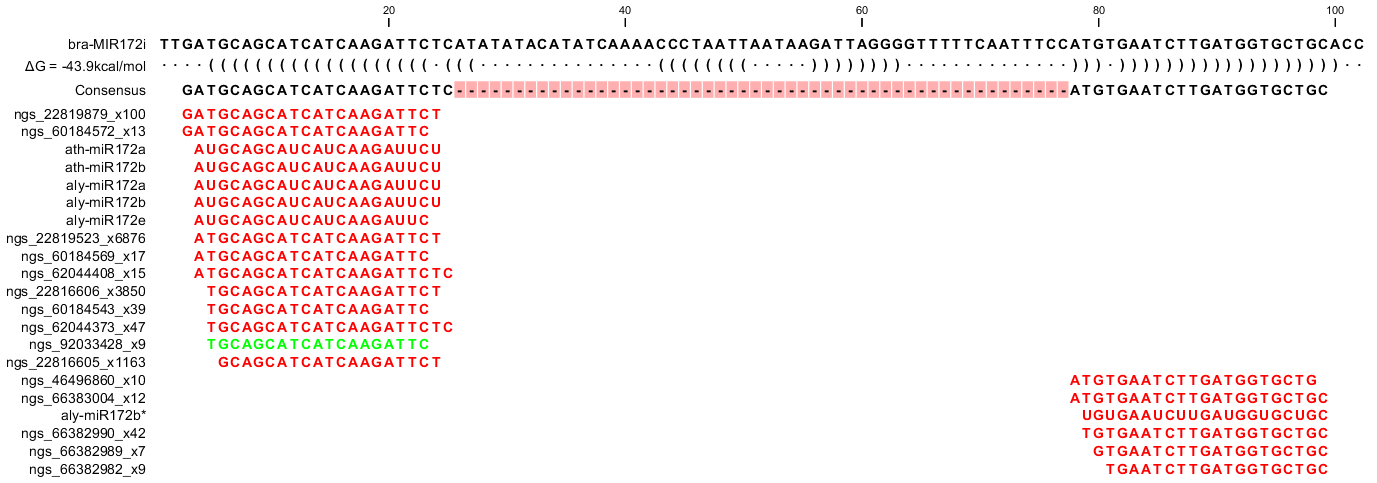


**MIR319**


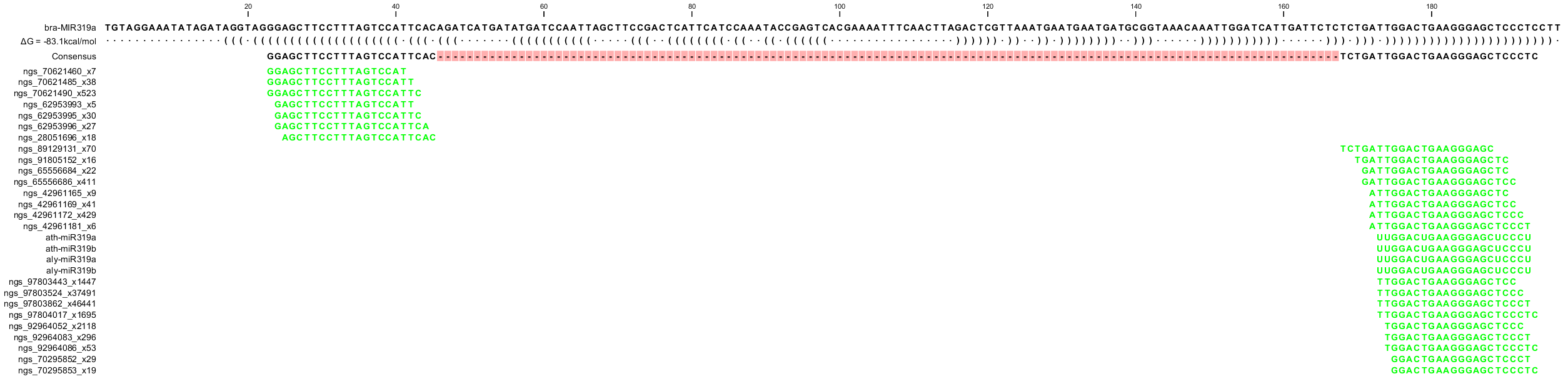

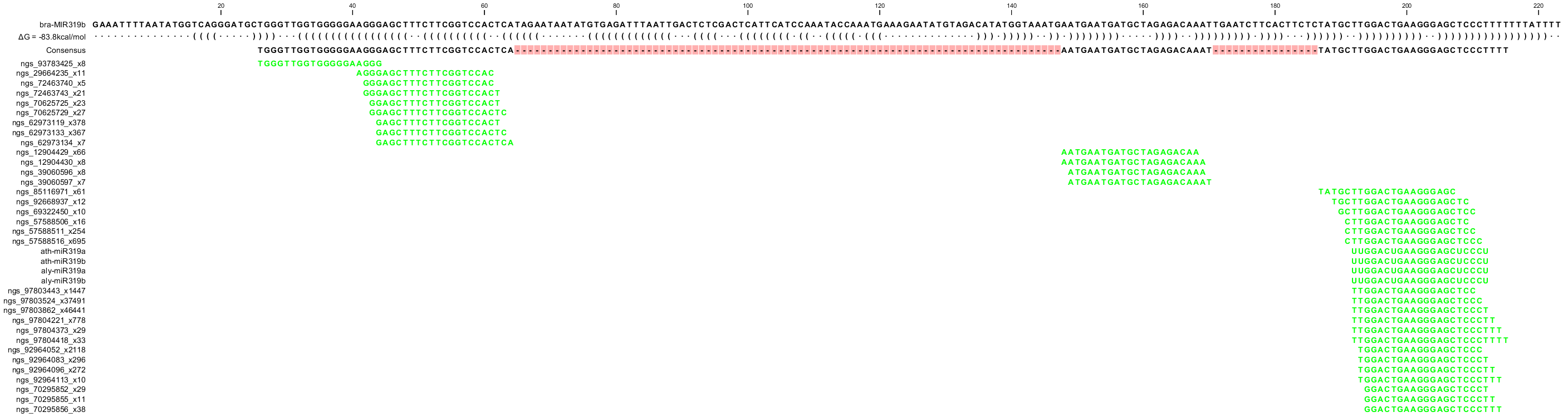

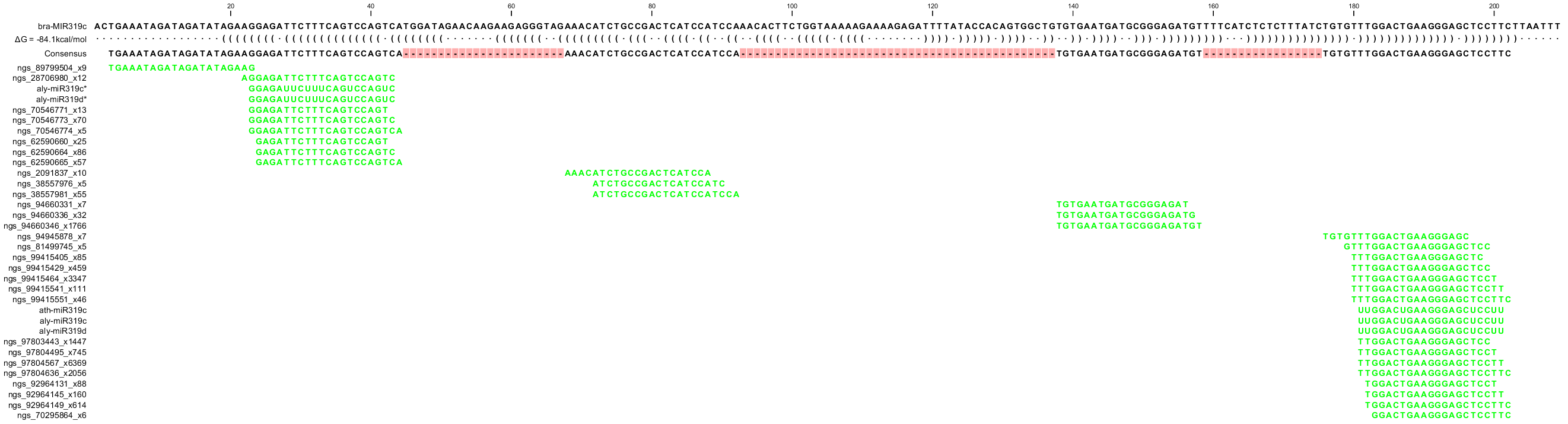

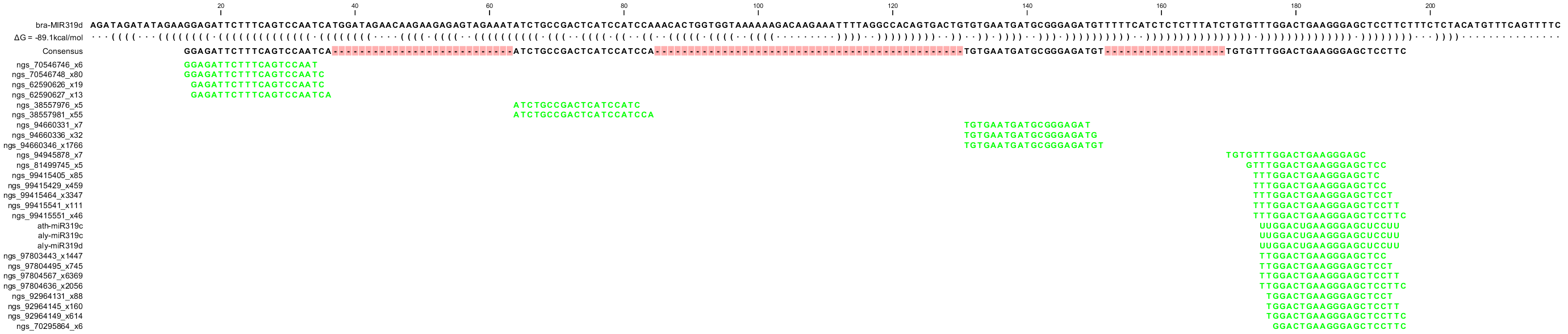


**MIR390**

**MIR391**

**bna-miR391a**

**bna-miR391b**

**MIR393**

**bna-miR393c**

**bna-miR393b**

**MIR394**

**MIR395**

**MIR396**

**MIR397**

**MIR398**

**MIR399**

**MIR400**

**MIR403**

**MIR408**

**bna-miR408**

**MIR824**

**bra-MIR824a**

**bra-MIR824b**

**bra-MIR824c**

**MIR827**

**MIR828**

**MIR838**

**bna-miR838**

**MIR845**

**MIR857**

**MIR858**

**MIR860**

**MIR1140**

**MIR1885**

**MIR2111**

**MIR5083**
