# Supplementary material for: MicroRNAs and their putative targets in Brassica napus seed maturation
Source: BMC Genomics. 2013 Feb 28;14:140. doi: 10.1186/1471-2164-14-140 (PMC3602245; doi:10.1186/1471-2164-14-140)

Figure S2. Putative novel *Brassica napus* MIRNAs based on the *Brassica* A genome (*Brassica rapa*). The read mapping patterns are displayed and the putative novel miRNA mature sequences underlined in red. Only reads with perfect matches are shown. Green represents the forward reads, red color represents reverse reads. The read count for each specific sequence was integrated into the sequence name as _x [read count of each unique sequence]. The secondary structures of each locus with their folding energy are shown below the genomic sequence.

MIR5801


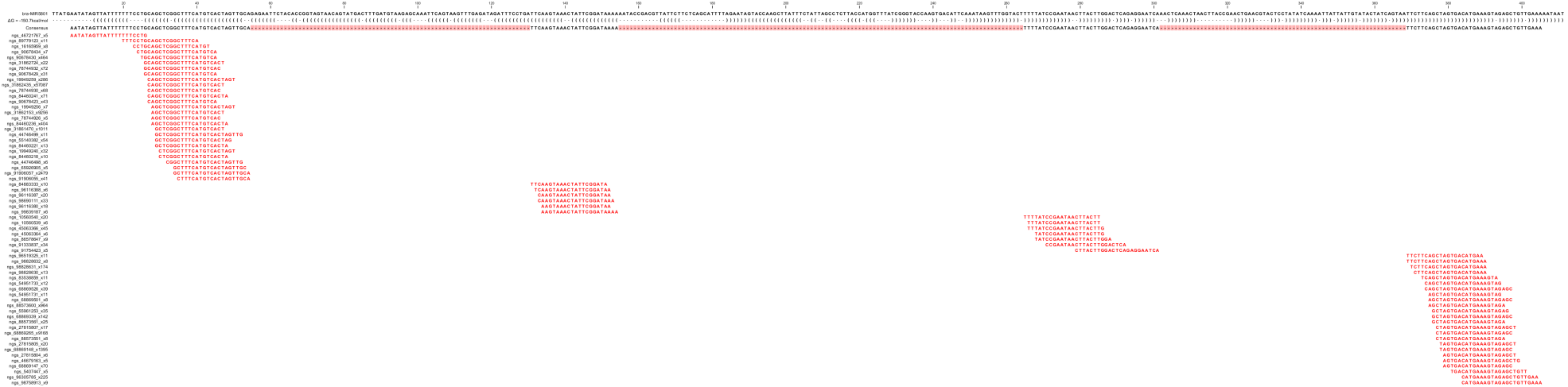


MIR5802


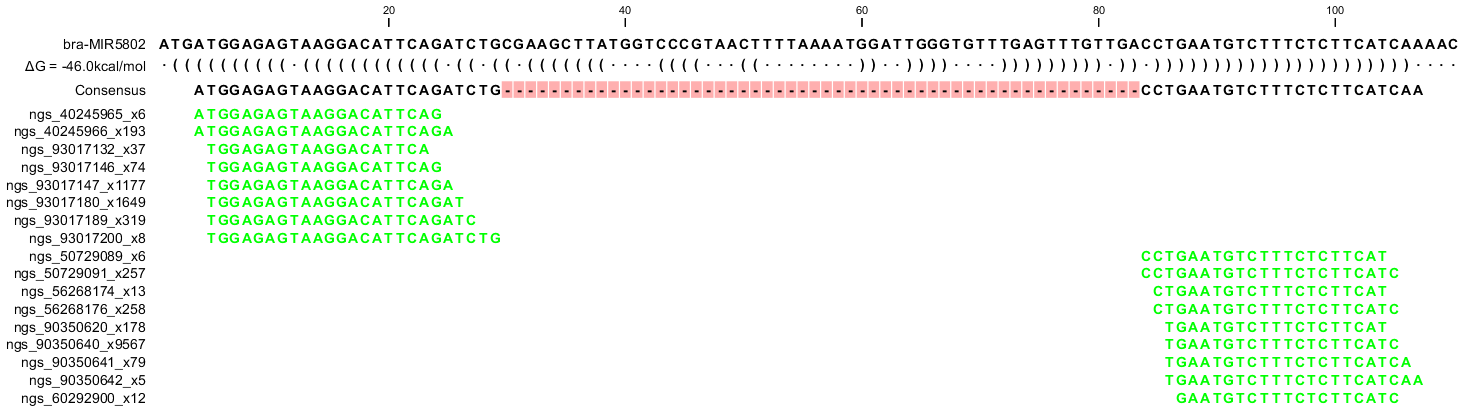


MIR5803


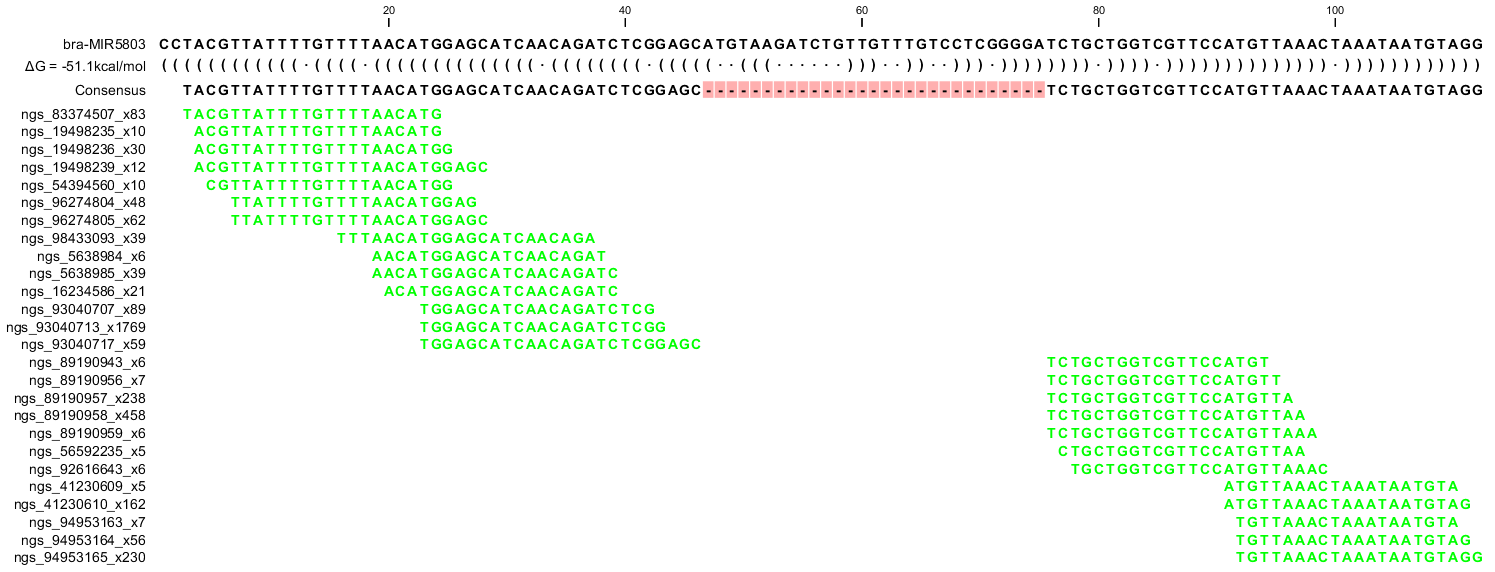


MIR5804


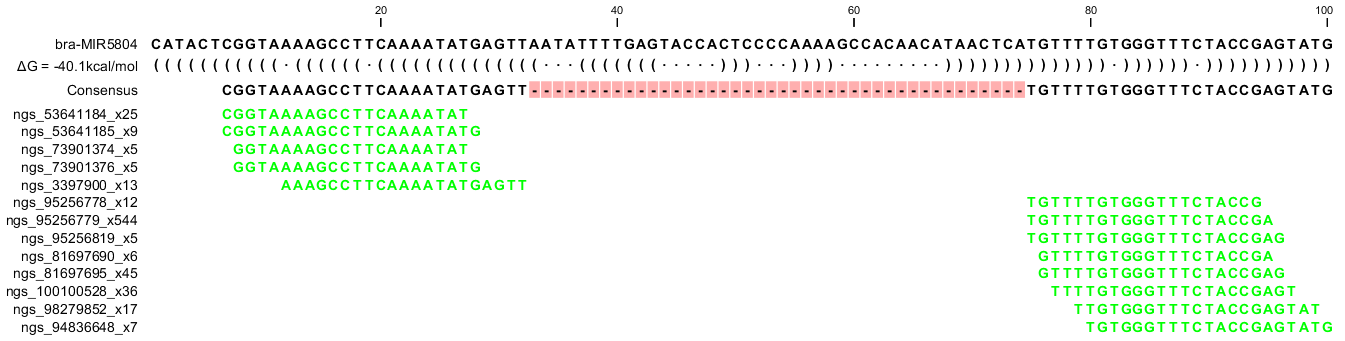


MIR5805


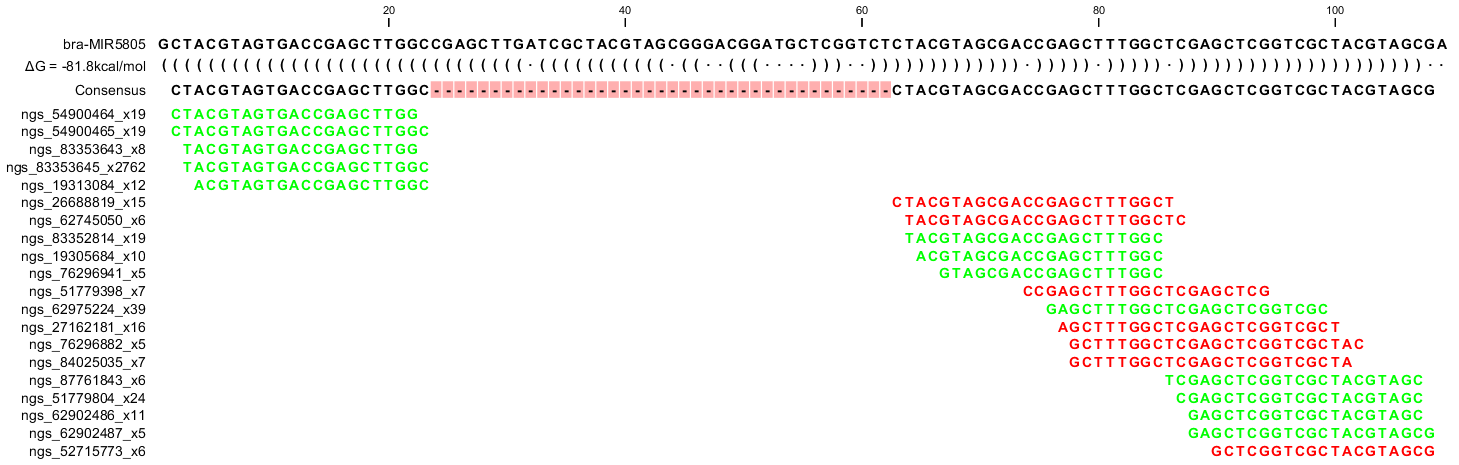


MIR5806


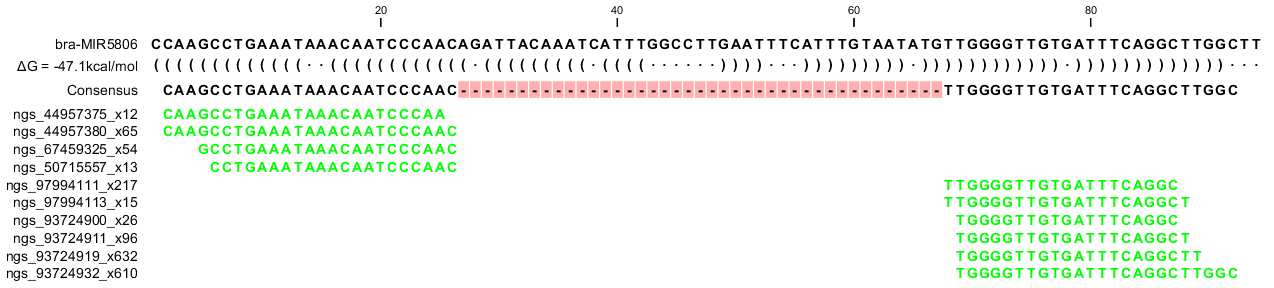


MIR5807a


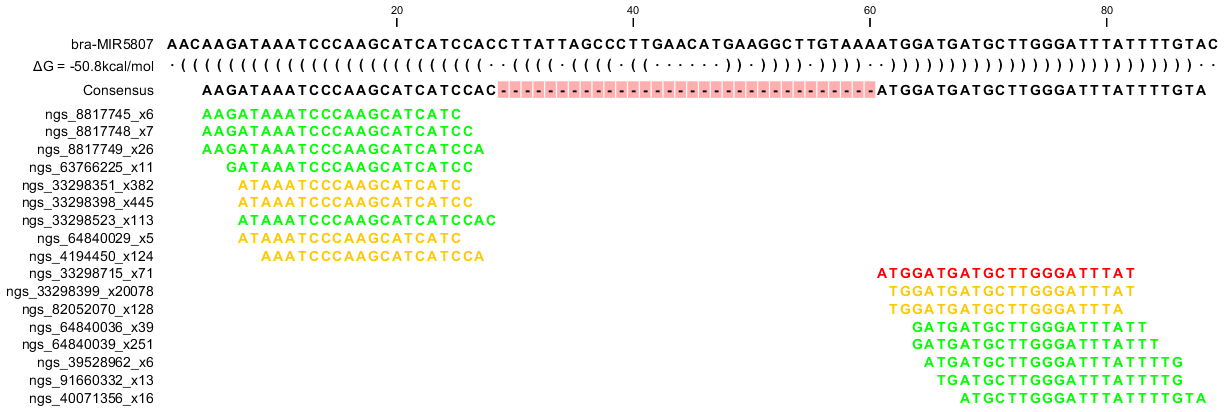


MIR5807b


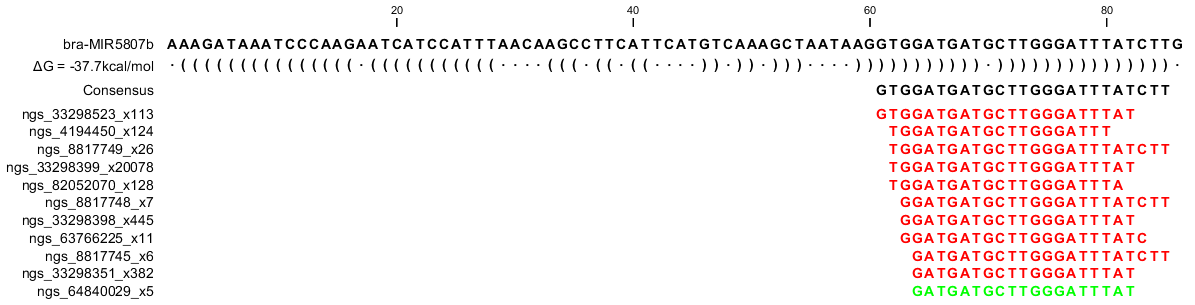


MIR5808


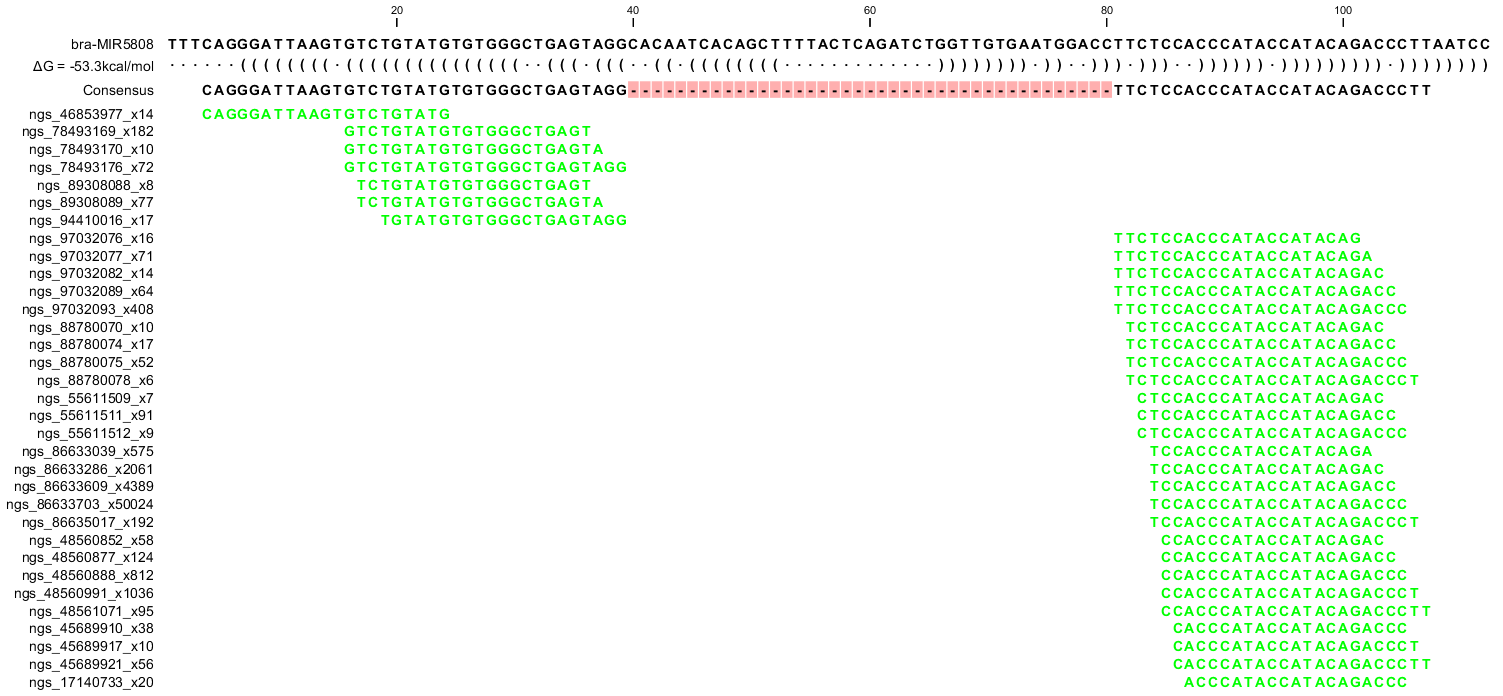


MIR5809


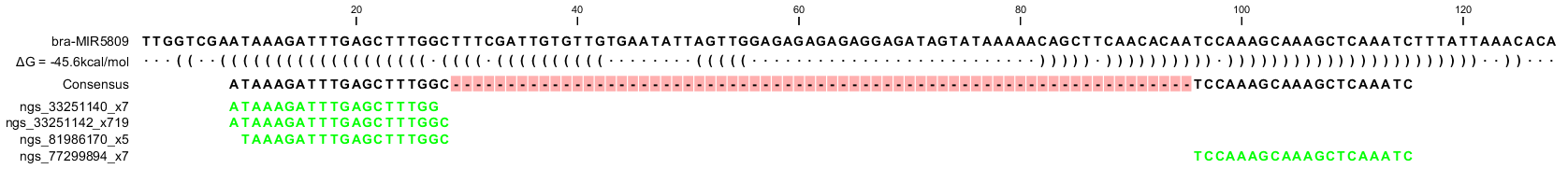


MIR5810


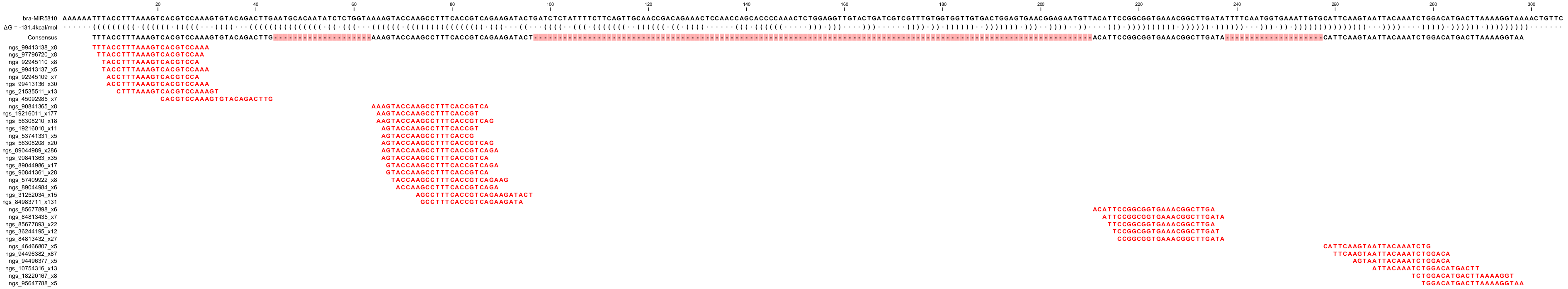

Supplement: Additional file 6: Figure S2 — Putative novel Brassica napus MIRNAs based on the Brassica A genome (Brassica rapa). The read mapping patterns are displayed and the putative novel miRNA mature sequences underlined in red. Only reads with perfect matches are shown. Green represents the forward reads, red color represents reverse reads. The read count for each specific sequence was integrated into the sequence name as _x [read count of each unique sequence]. The secondary structures of each locus with their folding energy are shown below the genomic sequence. [file 1471-2164-14-140-S6.docx]
