# Supplementary material for: MicroRNAs and their putative targets in Brassica napus seed maturation
Source: BMC Genomics. 2013 Feb 28;14:140. doi: 10.1186/1471-2164-14-140 (PMC3602245; doi:10.1186/1471-2164-14-140)

Supplemental Figure 3. Integration of putative miRNAs identified from Zhao et al. (2012) into mapping results reported in this paper. The mapping patterns of sequence reads to 10 putative miRNA loci are displayed. Three sequences from Zhao et al were also predicted as novel miRNAs in this study. They are miR2203, miR2204, miR2207, corresponding to our miR5802, miR5806, miR5803. miR2202 is identical to our miR5802*. Because the Zhao et al designations miR2202-miR2225 were assigned to other species in miRBase (Release 18), we retained our assigned numbers (miR5801-miR5810) for these novel putative miRNAs. Mapping patterns suggest that Zhao et al miRNAs 2205, 2206 and 2225 are actually siRNAs.


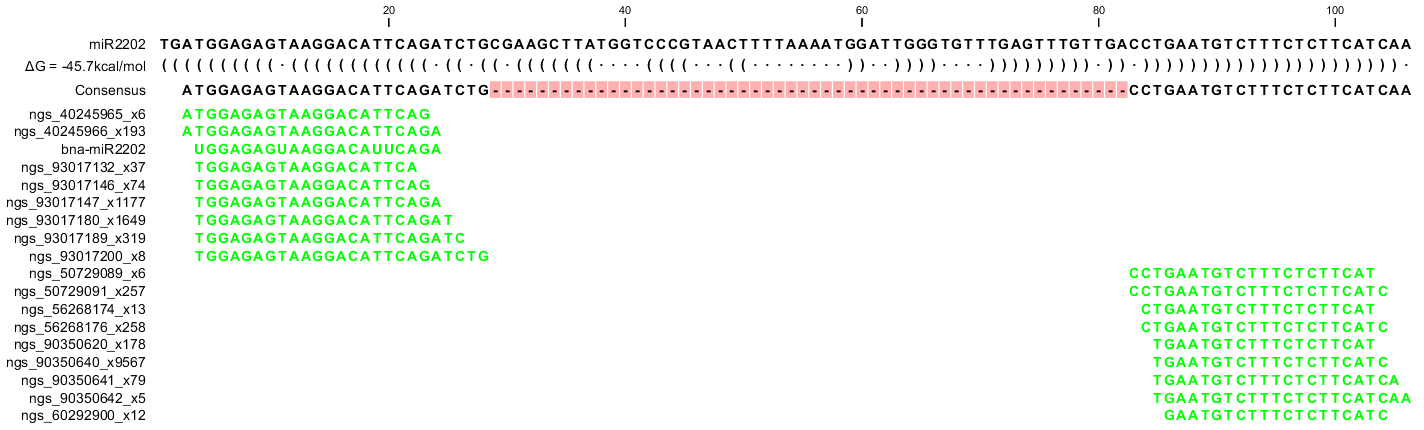

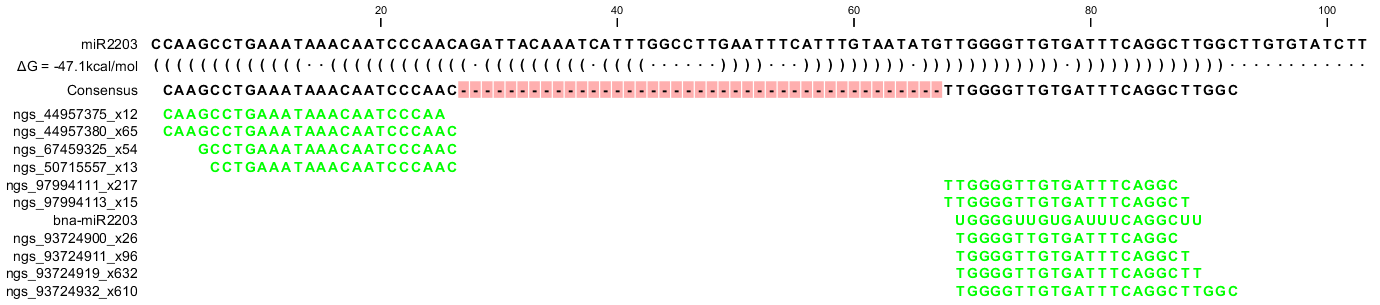

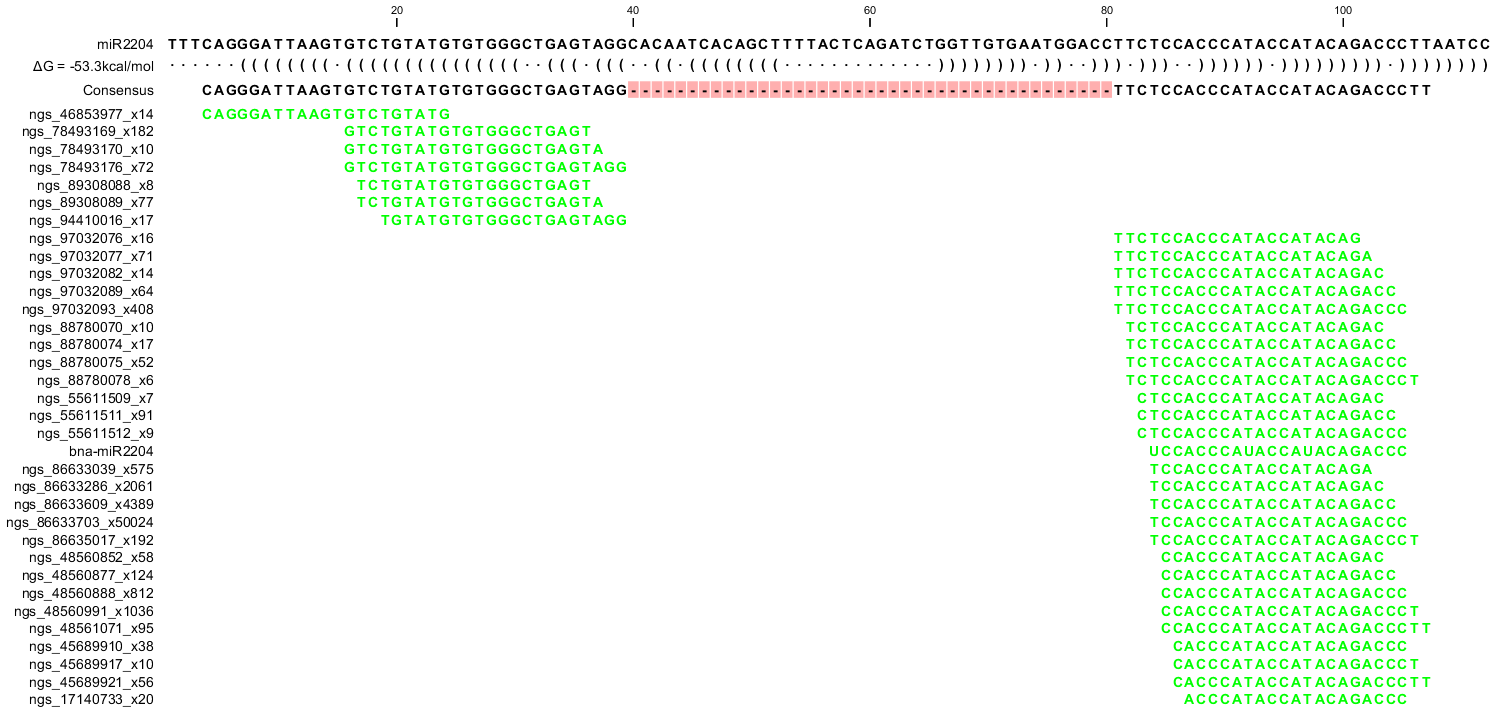


miR5808

miR5806

miR5802*

miR5802


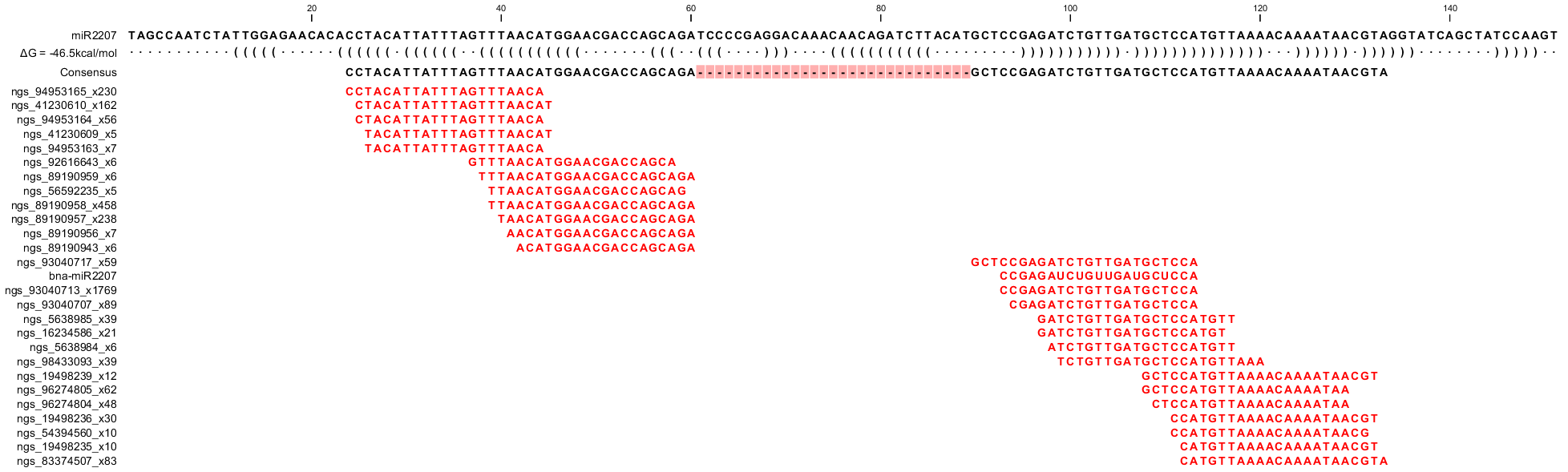

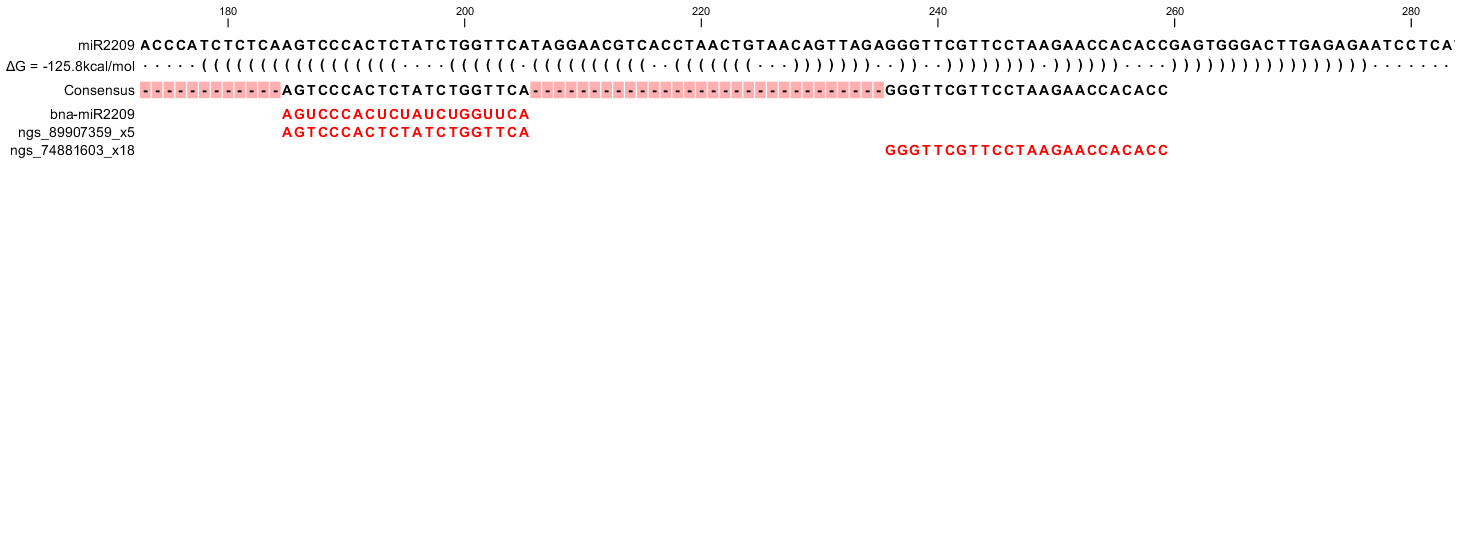

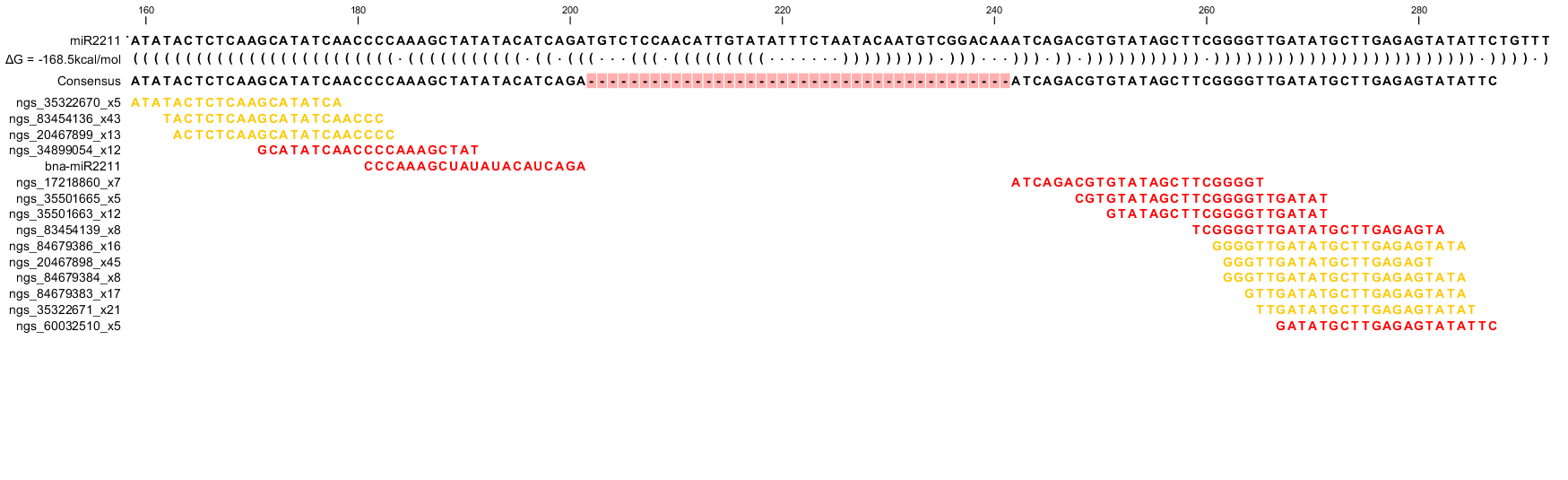

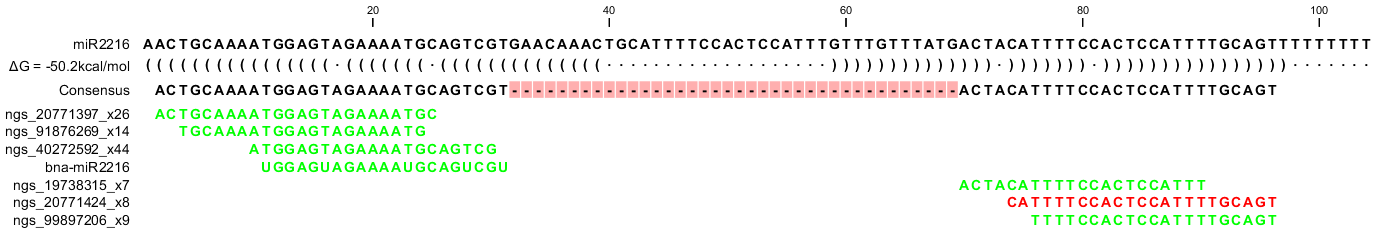

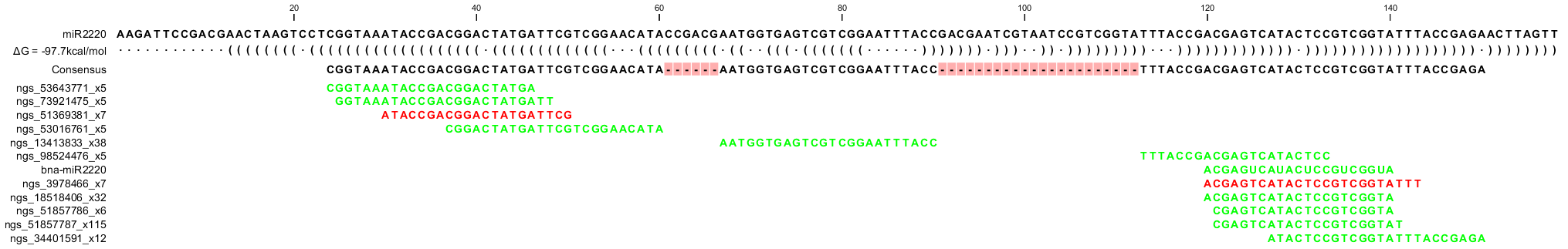


miR5803

miR2205


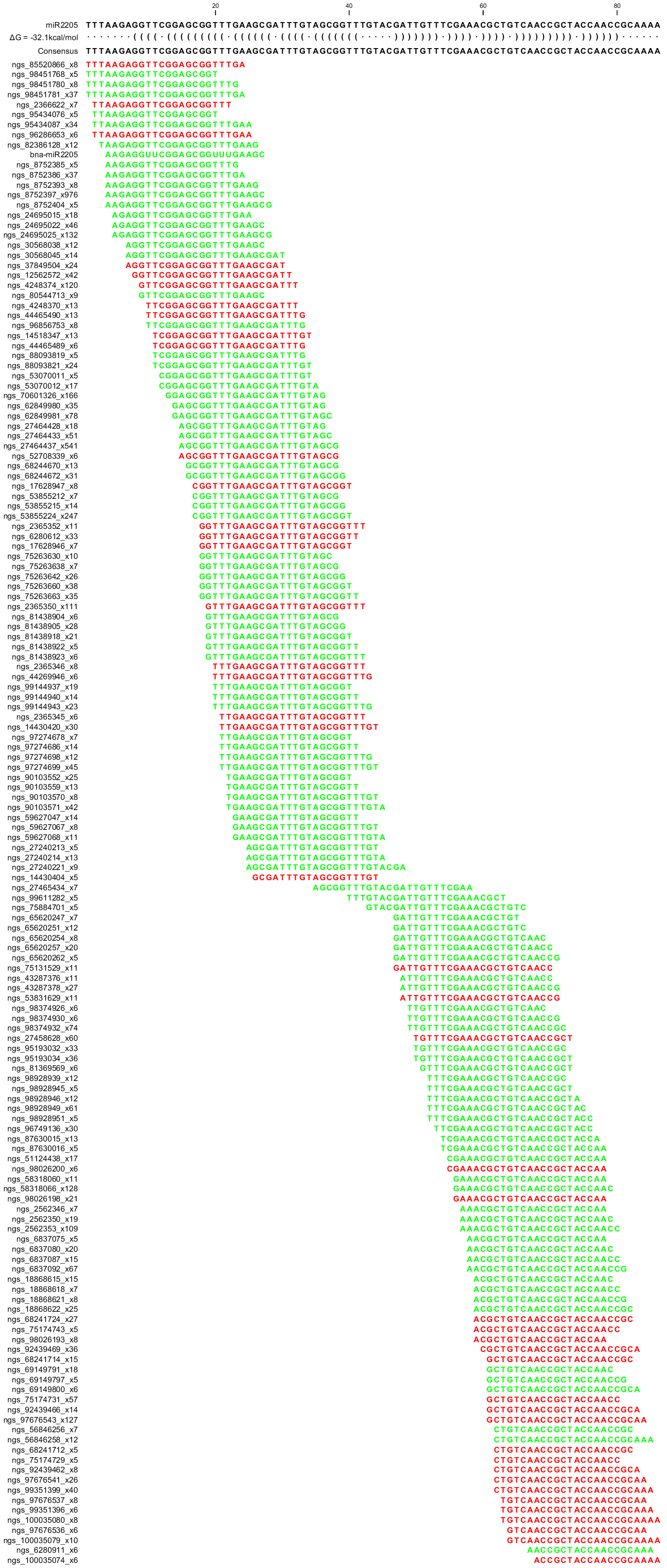


miR2206


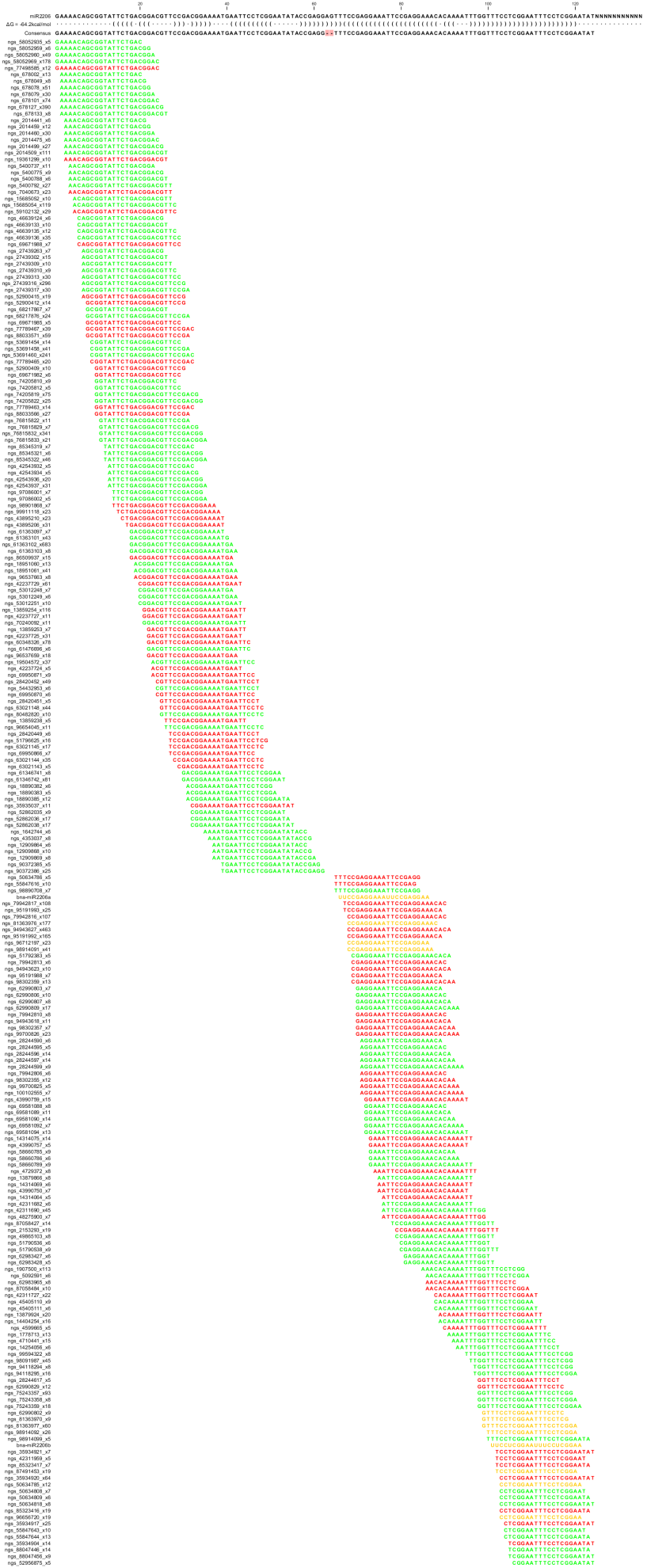


miR2225


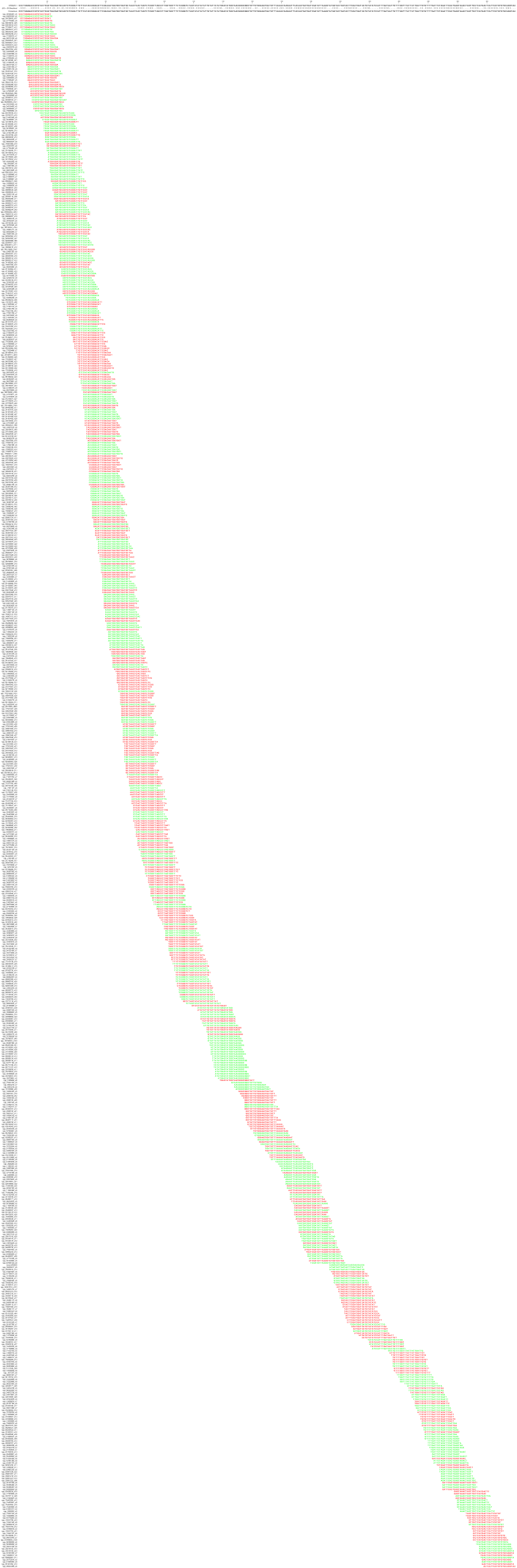

Supplement: Additional file 12: Figure S3 — Integration of putative miRNAs identified from Zhao et al. [15] into mapping results reported in this paper. The mapping patterns of sequence reads to 10 putative miRNA loci are displayed. Three sequences from Zhao et al. were also predicted as novel miRNAs in this study. They are miR2203, miR2204, miR2207, corresponding to our miR5802, miR5806, miR5803. miR2202 is identical to our miR5802*. Because the Zhao et al. designations miR2202-miR2225 were assigned to other species in miRBase (Release 18), we retained our assigned numbers (miR5801-miR5810) for these novel putative miRNAs. Mapping patterns suggest that Zhao et al. miRNAs 2205, 2206 and 2225 are actually siRNAs. [file 1471-2164-14-140-S12.docx]
