# Supplementary figures and images for: MicroRNAs and their putative targets in Brassica napus seed maturation
Source: BMC Genomics. 2013 Feb 28;14:140. doi: 10.1186/1471-2164-14-140 (PMC3602245; doi:10.1186/1471-2164-14-140)

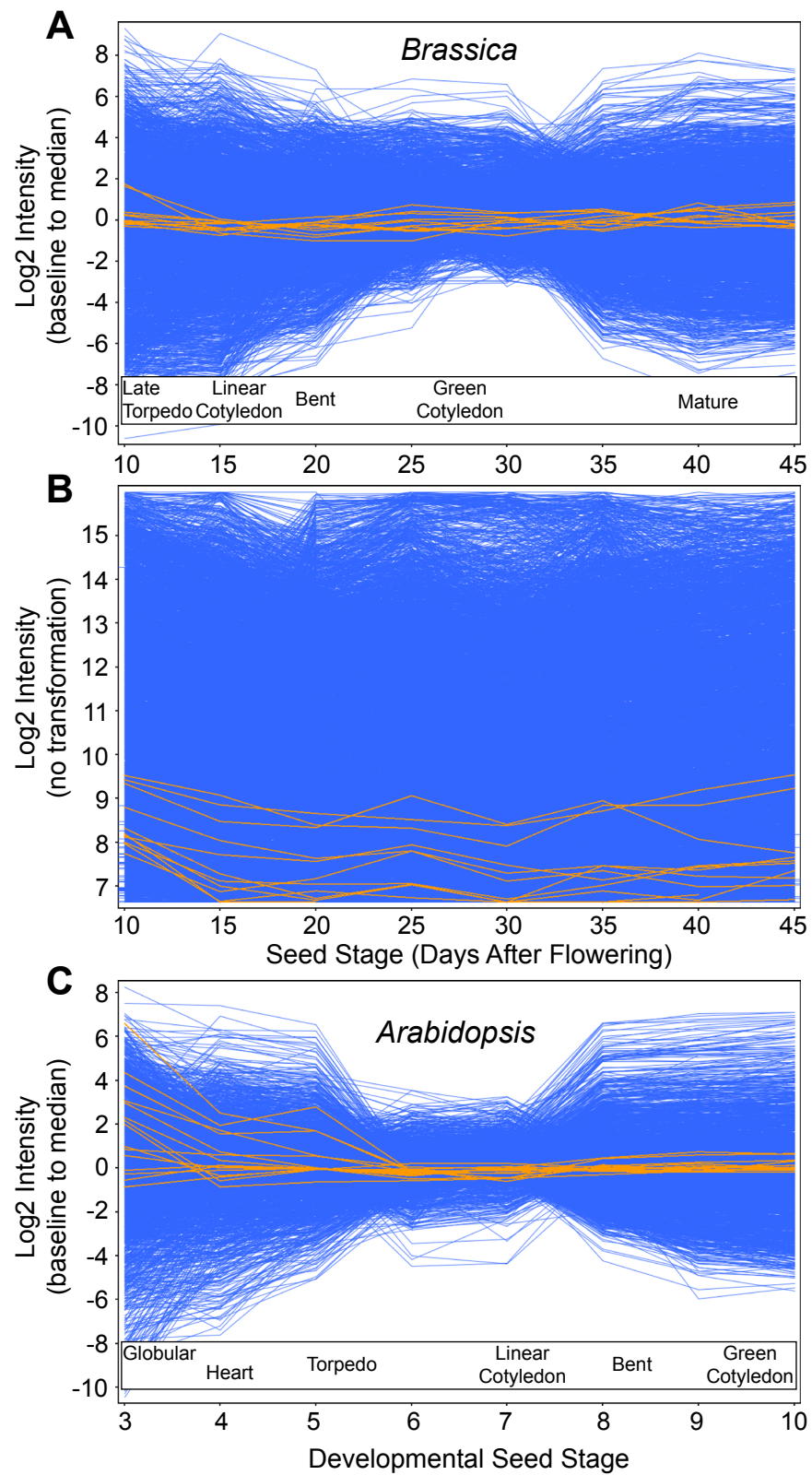

Supplemental Figure 4.

Supplement: Additional file 16: Figure S4 — Gene expression profiles during seed development and maturation. Each line represents one gene. A. Expression profiles of 90K Brassica EST contigs (Baseline transformed to median), miR156 targets -- SPLs are highlighted in orange. B. Expression profiles of 90K Brassica EST contigs (no transformation), SPLs are highlighted in orange. C. Expression profiles of 22K Arabidopsis genes (baseline transformed to median) during seed development (Schmid et al., 2005), SPLs are highlighted in orange. [file 1471-2164-14-140-S16.pdf]
