# Supplementary material for: MicroRNAs and their putative targets in Brassica napus seed maturation
Source: BMC Genomics. 2013 Feb 28;14:140. doi: 10.1186/1471-2164-14-140 (PMC3602245; doi:10.1186/1471-2164-14-140)
Supplement: Additional file 8: Figure S5 — BiNGO graph of enriched GO terms associated with miRNA targets (based on Arabidopsis homologs). The size of node indicates the number of genes in a GO category. The yellow and orange nodes represent terms with significant enrichment, with darker orange representing a higher degree of significance, as shown by the legend. White nodes are terms with no significant enrichment, but are included because they have a significant child term. Branches of GO with no significant terms are not shown. [file 1471-2164-14-140-S8.pdf]

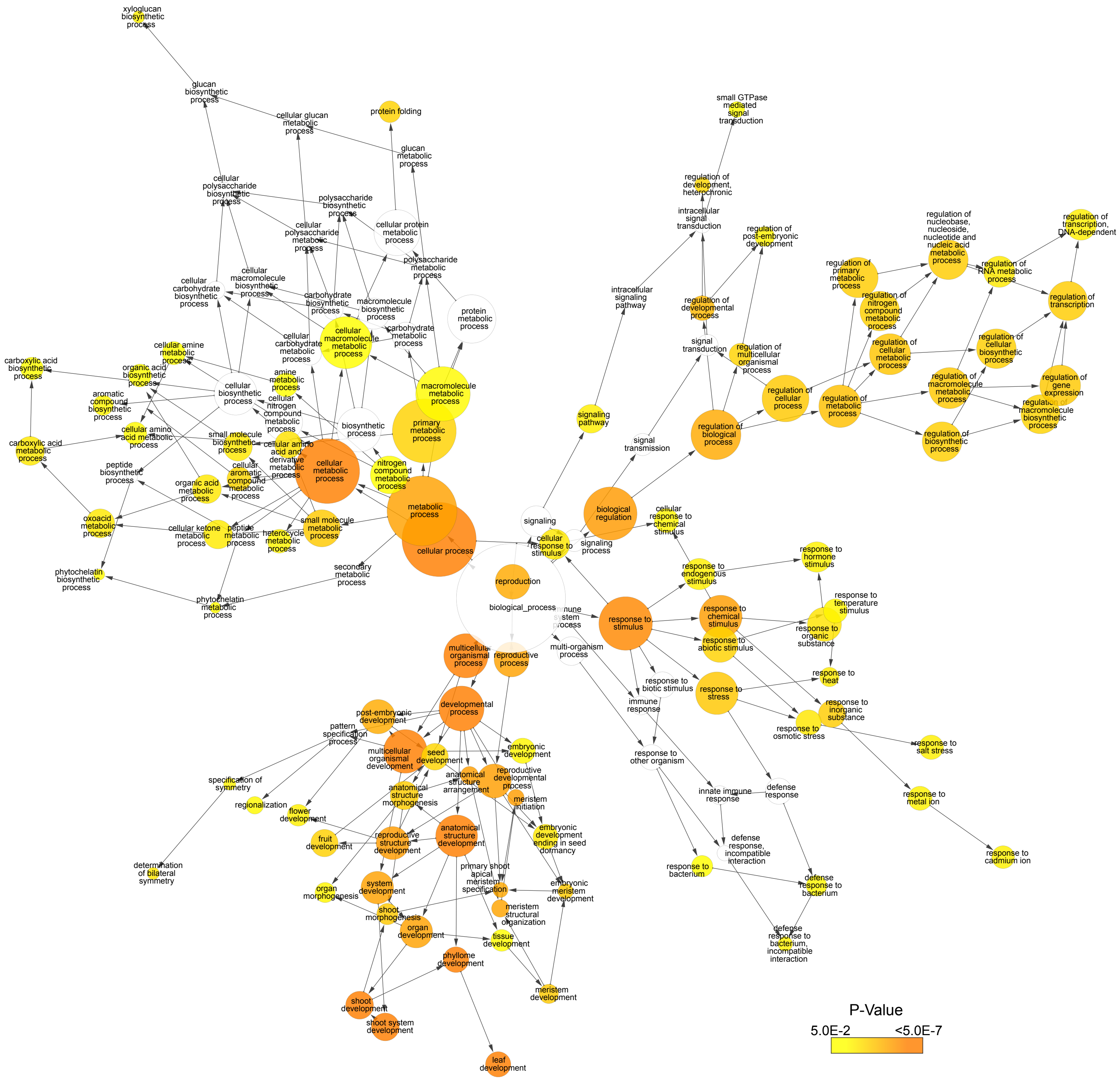

Supplemental Figure 5. BiNGO graph of enriched GO terms associated with miRNA targets (based on Arabidopsis homologs). The size of node indicates the number of genes in a GO category. The yellow and orange nodes represent terms with significant enrichment, with darker orange representing a higher degree of significance, as shown by the legend. White nodes are terms with no significant enrichment, but are included because they have a significant child term. Branches of GO with no significant terms are not shown.
